# Exonic trinucleotide repeat expansions in *ZFHX3* cause spinocerebellar ataxia type 4: A poly-glycine disease

**DOI:** 10.1016/j.ajhg.2023.11.008

**Published:** 2023-11-29

**Authors:** Joel Wallenius, Efthymia Kafantari, Emma Jhaveri, Sorina Gorcenco, Adam Ameur, Christin Karremo, Sigurd Dobloug, Kristina Karrman, Tom de Koning, Andreea Ilinca, Maria Landqvist Waldö, Andreas Arvidsson, Staffan Persson, Elisabet Englund, Hans Ehrencrona, Andreas Puschmann

**Affiliations:** 1 Neurology, Department of Clinical Sciences Lund, Lund University, Skåne University Hospital, 222 42 Lund, Sweden; 2 Department of Immunology, Genetics and Pathology, Science for Life Laboratory, Uppsala University, 751 23 Uppsala, Sweden; 3 Department of Neurology, Helsingborg General Hospital, 252 23 Helsingborg, Sweden; 4 Division of Clinical Genetics, Department of Laboratory Medicine, Lund University, 222 42 Lund, Sweden; 5 Department of Clinical Genetics, Pathology and Molecular Diagnostics, Office for Medical Services, Region Skåne, 221 85 Lund, Sweden; 6 Pediatrics, Department of Clinical Sciences Lund, Lund University, 221 84 Lund, Sweden; 7 Division of Clinical Sciences Helsingborg, Department of Clinical Sciences Lund, Lund University, 221 84 Lund, Sweden; 8 Pathology, Department of Clinical Sciences Lund, Lund University, Skåne University Hospital, 222 42 Lund, Sweden; 9 SciLifeLab National Research Infrastructure, Lund University, 221 84 Lund, Sweden

**Keywords:** spinocerebellar ataxia 4, SCA4, ZFHX3, trinucelotide repeats, poly-glycine, autosomal dominant, anticipation, interruptions, GGC repeats, p62-positive inclusions

## Abstract

Autosomal-dominant ataxia with sensory and autonomic neuropathy is a highly specific combined phenotype that we described in two Swedish kindreds in 2014; its genetic cause had remained unknown. Here, we report the discovery of exonic GGC trinucleotide repeat expansions, encoding poly-glycine, in zinc finger homeobox 3 (*ZFHX3*) in these families. The expansions were identified in whole-genome datasets within genomic segments that all affected family members shared. Non-expanded alleles carried one or more interruptions within the repeat. We also found *ZFHX3* repeat expansions in three additional families, all from the region of Skåne in southern Sweden. Individuals with expanded repeats developed balance and gait disturbances at 15 to 60 years of age and had sensory neuropathy and slow saccades. Anticipation was observed in all families and correlated with different repeat lengths determined through long-read sequencing in two family members. The most severely affected individuals had marked autonomic dysfunction, with severe orthostatism as the most disabling clinical feature. Neuropathology revealed p62-positive intracytoplasmic and intranuclear inclusions in neurons of the central and enteric nervous system, as well as alpha-synuclein positivity. *ZFHX3* is located within the 16q22 locus, to which spinocerebellar ataxia type 4 (SCA4) repeatedly had been mapped; the clinical phenotype in our families corresponded well with the unique phenotype described in SCA4, and the original SCA4 kindred originated from Sweden. ZFHX3 has known functions in neuronal development and differentiation n both the central and peripheral nervous system. Our findings demonstrate that SCA4 is caused by repeat expansions in *ZFHX3*.

## Graphical abstract

**Figure:**
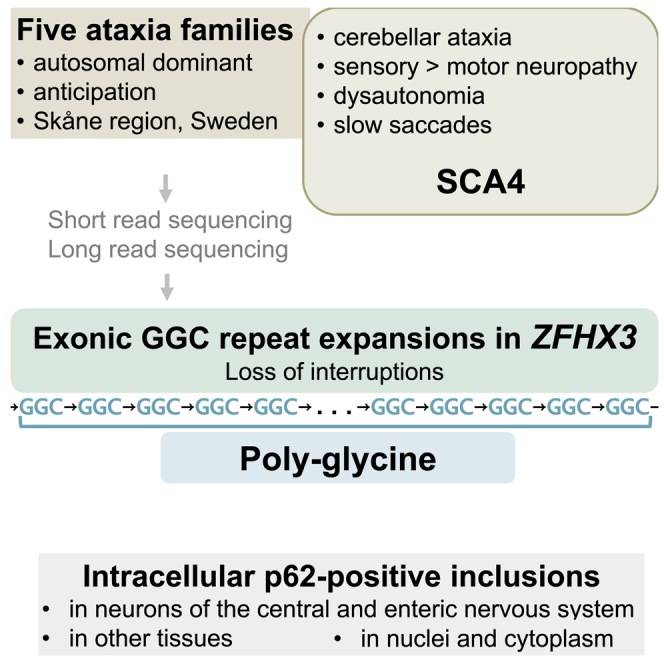

## Teaser

In five families from Skåne region in Sweden, expanded repeat expansions in *ZFHX3* were identified as the cause for spinocerebellar ataxia type 4, SCA4. SCA4 is an autosomal-dominant disorder with progressive gait and balance disturbance and dyscoordination of movements. SCA4 might compromise nerves controlling automatic bodily functions.

## Introduction

An increasing number of genes associated with hereditary ataxias are being discovered, as a result of the increasing availability and constant improvements in next-generation sequencing (NGS) technology.^1^^,^^2^ Hereditary ataxias form a broad spectrum of different entities, and cerebellar ataxia with neuropathy defines a certain subgroup within this spectrum.^3^ In 2014, we described two kindreds with autosomal-dominant cerebellar ataxia and sensory and autonomic neuropathy.^4^ The affected family members also had a characteristic slowness of horizontal saccades, and their ancestors could genealogically be traced to the same village in southern Sweden. We now report the discovery of exonic trinucleotide repeats in *ZFHX3* (MIM: 104155) and find that these repeats co-segregate with the disease phenotype in these two families. We have expanded the previously reported family’s pedigree, provide clinical follow-up data 8–9 years after our first description, and report on the neuropathology of one affected individual who died at the age of 28 years. We identified four additional persons with ataxia from three independent Swedish families with the same disease phenotype and *ZFHX3* repeat expansions from our ataxia series, but the repeat expansions were absent from large in-house and national datasets from individuals with other diseases and from population controls. The gene’s location within the previously described locus for spinocerebellar ataxia type 4 (SCA4 [MIM: 600223]) and the clinical overlap with SCA4 suggest that *ZFHX3* repeat expansions cause SCA4. This disorder might be a relatively common cause of autosomal-dominant ataxias, at least in the southern Swedish population.

## Subjects and methods

### Genealogical data and clinical examination

**Figure 1:**
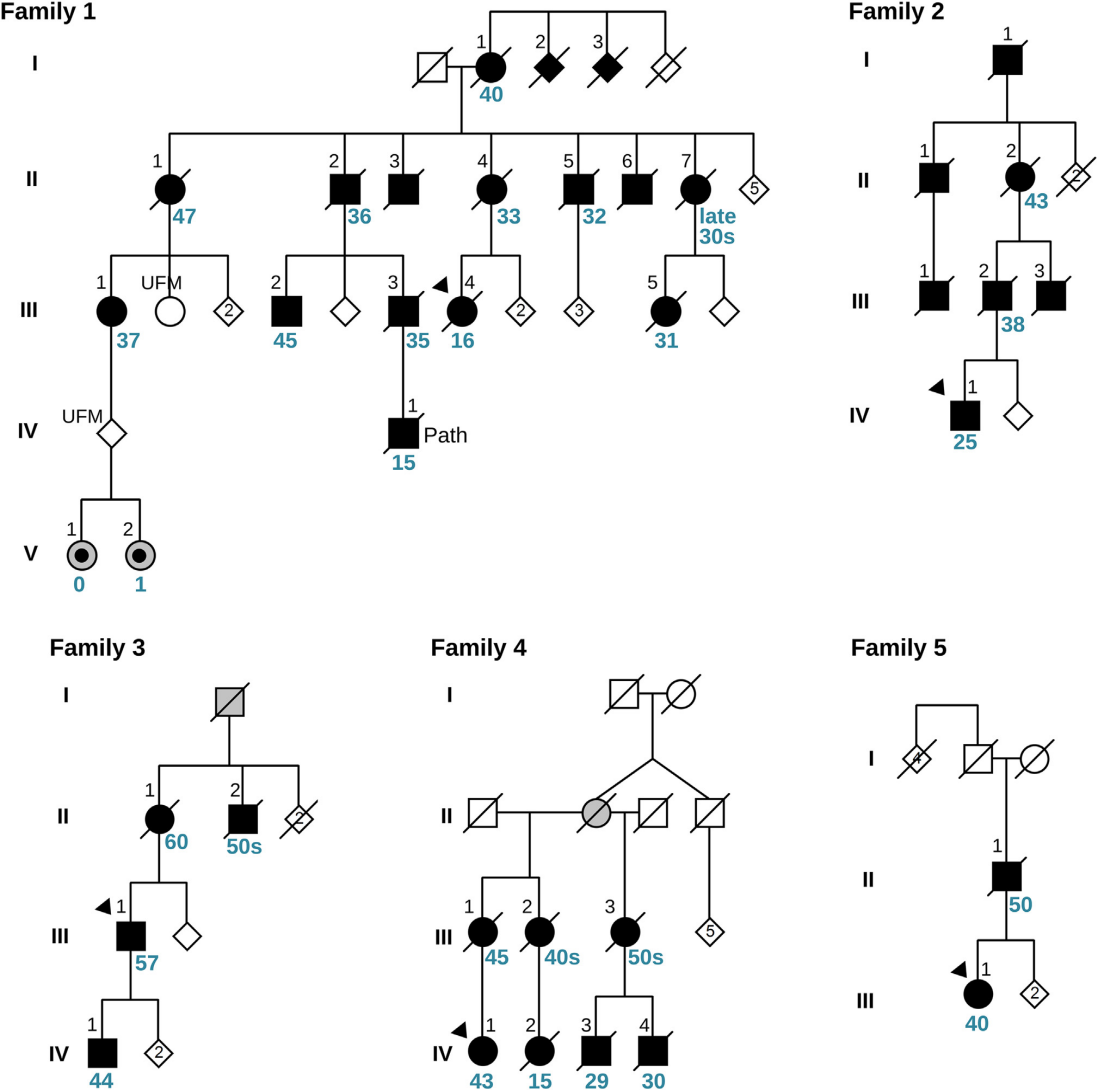
Family pedigrees Standard symbols were used. Black symbols represent individuals with cerebellar ataxia with sensory and autonomic neuropathy. Probands are indicated with black triangles. Gray symbols show family members who had gait and balance problems according to family history. Gray symbols with a central black dot represent two siblings who were evaluated within this study but were considered to have a neurodevelopmental disorder that is different from cerebellar ataxia with sensory and autonomic neuropathy, and who do not have *ZFHX3* repeat expansions. The black numbers above the symbols are individual identifiers. For those family members for whom age at symptom onset was available, it is shown below the symbols in blue. Sex and gender of non-affected individuals was disguised, and sibling order partly altered to protect confidentiality. For the same reason, a few unaffected family members are not shown. UFM, unaffected family member who was analyzed genetically within this study.

Family 1 and family 2 were described in 2014 by some of the authors,^4^ and the authors had since remained in contact with the families and continuously cared for some of their affected members at their clinics. Family 2 had been described in 1978.^5^ Additional affected members were identified via the families and/or from among persons with ataxia in the authors’ clinics. Updated pedigree drawings are shown in Figure 1. All surviving affected family members were re-contacted in 2022, and all were interviewed and re-examined in a standardized manner in the context of the present study. As for the examinations for our 2014 publication, the international cooperative ataxia rating scale (ICARS) was used for examining and comparing specific ataxia symptoms^6^; affected members from the newly identified SCA4-affected families had been examined according to the scale for the assessment and rating of ataxia (SARA).^7^ After the identification of three additional families carrying *ZFHX3* repeat expansions from our ongoing ataxia research study,^8^ their members were also re-approached for this study. Medical records of deceased and alive affected members were reviewed. Our study was approved by the Regional Ethics Review Board in Lund and the Swedish Ethical Review Authority. All adult participants provided informed consent. Two children were examined by a pediatrician in the context of their clinical evaluation; written informed consent was provided by their parents.

### Genetic analysis

Blood was drawn from affected and unaffected individuals. Short-read whole-genome sequencing was performed at the Department of Clinical Genetics, Region Skåne, the Center for Translational Genomics, Lund University or at Centogene, Rostock, Germany. Clinical reports from whole-genome examinations filtered for ataxia and neuropathy genes that include known clinically relevant repeat expansions were obtained but failed to elucidate the cause of the disease. We continued to analyze WGS datasets from families 1 and 2 to search for variants in genes not previously related to neurological diseases or ataxias. We explored different approaches depending on whom we assumed to be affected by the same disease; we analyzed only family 1 or both families together, and we either included or excluded family 1’s two young children (V:1 and V:2), who had a different clinical phenotype that included infantile onset and cognitive and behavioral symptoms, albeit with ataxia. Family 1 III:UFM and other in-house genomes from unrelated individuals without ataxia were used as references where needed.

Genomic segments shared by affected but not by unaffected family members were detected by identity-by-descent (IBD) analysis. We predicted IBD segments by first phasing variant calling format (VCF) files from families 1 and 2 with Beagle v5.4^9^ and using the 1000 Genomes reference panel, then running hap-ibd.^10^ Non-default settings for hap-ibd are provided in Table S1, and a full description of each setting is available in the hap-ibd documentation (https://github.com/browning-lab/hap-ibd).

Single-nucleotide variants (SNVs) were either supplied in VCF files by the sequencing cores or produced from supplied binary alignment map (BAM) files via the Genome Analysis Toolkit v.4.2.6.1.^11^ VCF files were annotated and filtered with VEP v.109^12^ so that variants with minor-allele frequencies above 0.01 were excluded. Copy-number variants (CNVs) were called with the Genome Analysis ToolKitv.4.2.6.1 according to the Broad Institute’s CNV calling tutorial (https://gatk.broadinstitute.org/hc/en-us/articles/360035531152), with default settings. The input-intervals file spanned the entire GRCh37 (hg19) genome in 1,000 bp intervals. SNV and CNV calls were cross-correlated with the genomic segments shared by affected members and curated manually with the Integrative Genomics Viewer v.2.12.2 for CNVs.^13^ CNVs that fit the co-segregation patterns were manually screened with DECIPHER^14^ so that common CNVs would be excluded.

Known short tandem repeat (STR) expansion loci were examined with ExpansionHunter v.5.0.0.^15^ The bundled variant catalog was extended to include the more recently described *FGF14*^2^^,^^16^ and *NOTCH2NLC*^17^ pathogenic repeats that cause similar clinical or pathological phenotypes. No expansions were found in either of these loci. STR expansion loci not previously associated with disease were assessed with ExpansionHunter Denovo v.0.9.0 from 2020,^18^ which is still the most recent version. Results were cross-correlated with the shared genomic segments and manually curated. From this emerged a trinucleotide repeat expansion in *ZFHX3* as a single finding. This expansion was confirmed by its addition to the ExpansionHunter variant catalog, the re-running of ExpansionHunter, and finally visual examination of the locus with graphics produced by REViewer v.0.2.7.^19^

### Screening of larger datasets

After the identification of expanded *ZFHX3* repeats associated with the disease phenotype in families 1 and 2, we screened additional available datasets for this repeat expansion. We queried 25 WES and 64 WGS datasets from 89 persons with ataxia of unresolved cause from our ataxia series, including those previously published.^8^ We also analyzed *ZFHX3* repeats in 90 WES and 60 WGS datasets from individuals with other neurological diagnoses in our research database and queried the SweGen dataset with Illumina short-read WGS data from 1,000 unrelated Swedish individuals, most of whom (942) were probands selected from more than 85,000 twins in the Swedish Twin Registry, as previously described.^20^ We analyzed the architecture of normal repeats in our 90 WES and 60 WGS in-house datasets (300 alleles) and in all 2,000 alleles from SweGen with ExpansionHunter and REViewer.

### Analysis of shared haplotype of the five families

We performed three additional IBD predictions, for which we added WGS data from members of families 3, 4, and 5, to confirm a shared haplotype and to investigate a possible relation between all five families. We searched for SNVs with the lowest frequency in population databases (gnomAD NFE and SweGen) within the IBD genomic area that included *ZFHX3* and compared their occurrence among affected and unaffected members of all five families.

### Long-read sequencing

Long-read genome sequencing was performed on DNA from individuals III:1 and IV:1 from family 1. These individuals were selected because they displayed a large difference in disease severity and age at onset (37 and 15 years). The samples were run on the PacBio Revio instrument, generating 77.2 Gb and 56.5 Gb of high-quality (>QV20) long-read data for individuals III:1 and IV:1, respectively. PacBio long-read data from 27 individuals from the Human PanGenome Consortium^21^ were used for comparison. We analyzed the data manually to determine the exact number and architecture of repeats in *ZFHX3* and to exclude structural changes within the 16q22 SCA4 locus.

### Neuropathology

Individual IV:1 from family 1 died unexpectedly at the age of 28 years, and a clinical postmortem examination was performed to elucidate the cause of his death. An extensive neuropathological examination of the central and peripheral nervous system was performed according to established procedures at the Department of Pathology, Region Skåne. It included conventional and immunohistochemical stains of brain regions and of autonomous nerves in the skin, gastrointestinal plexus, and epicardium. Antibodies against hyperphosphorylated tau, alpha-synuclein, and TDP-43 were used as described previously.^22^ For evaluation of p62 protein pathology, anti-nucleoporin/p62 LCK ligand manufactured by BD Transduction Laboratories (clone 3, catalog no. 610832) was used in 1:100 dilution.

## Results

### Genetic analysis of families 1 and 2

**Figure 2:**
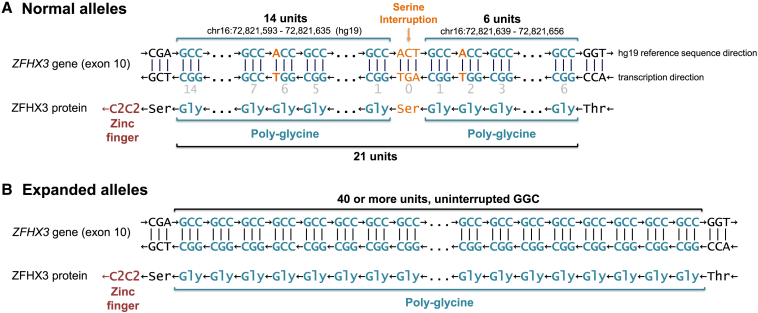
*ZFHX3* locus harboring the repeat expansion encoding poly-glycine *ZFHX3* is encoded on the negative strand in the hg19 reference genome. For clarity, the negative strand sequence is included in the figure. Unless otherwise specified, we discuss the repeat region from the perspective of the negative strand. (A) In the vast majority of non-expanded alleles, the repeat region consists of exactly 18 GGC units, two GGT units, and a single AGT interruption; we report the total repeat length as 21 for these alleles. The locations of the GGT units (which also code for glycine but interrupt the repetitive GGC pattern on the DNA level) are given relative to the AGT interruption (light gray numbers). Such a normal allele results in a protein with 20 glycine residues interrupted by a single serine at position 7. This poly-glycine is followed by a single serine residue and a C2C2-type zinc finger motif. Generally, zinc finger motifs are considered elements that can bind to DNA in a sequence-specific manner. See main text for the exact composition of this locus in the minority of non-expanded alleles. (B) There were no detectable interruptions in any of the expanded alleles from the affected members of the five families described here—the repeat region was entirely composed of GGC units.

No deleterious single-nucleotide variant, indel, copy-number variant, or previously described short tandem repeat co-segregating with the disease was identified in families 1 or 2. We did not identify a common genetic cause when including the two individuals with a severe neurodevelopmental phenotype (family 1, V:1 and V:2) and excluded these from subsequent analyses. Our IBD analyses revealed 199 genomic segments shared by all analyzed affected individuals in family 1, except V:1 and V:2. These segments ranged in length from 1 kbp to 2.7 Mbp, with a median of 108 kbp; one of the longest included a 1.6 Mbp segment on chromosome 16. When the proband of family 2 (IV:1) was included, the shared segment on chromosome 16 was narrowed down to 394 kbp (Table S1). Within this genomic segment, a GGC (poly-glycine) repeat expansion in the final exon of *ZFHX3* (MANE Select transcript, GenBank: NM_006885.4) co-segregated perfectly with disease status in both families (Figure 2; Tables 1 and S2). The two children (family 1, V:1 and 2) and their unaffected parent had normal *ZFHX3* repeat lengths, as did the unaffected family members in family 1 (III:UFM and IV:UFM). Long-read sequencing revealed the exact length of the repeat expansion to be 57 uninterrupted GCC repeats in family 1 III:1 (age of onset 37 years) and 74 uninterrupted GCC repeats in IV:1 (age of onset 15 years).

**Table 1:** Clinical phenotype and genetic results of affected members of family 1

	**Family 1**											
**Individual**	**II:1**	**II:2**	**II:4**	**II:5**	**III:1**	**III:2**	**III:3**	**III:4**	**III:5**	**IV:1**	**V:1**	**V:2**
Age at onset (years)	47	36	33	32	37	45	35	16	31	15	0.5	1.5
Age at most recent evaluation or age at death	78	70	75	60	66	57	47	42	44	28	8	4
Initial symptom(s)	BD, numbness in feet	BD, intention tremor	BD	BD	BD	BD	gait and limb ataxia	BD	BD	BD	delayed psycho-motor development	abnormal gait, limb ataxia
Walking aid (most recent documented)	wheelchair	wheelchair	wheelchair	wheelchair	wheelchair	wheelchair	wheelchair	wheelchair	wheelchair	walker at home, wheelchair outside	walker	none
Dysarthria	++	++	yes	+++	+++	+	+++	+++	+++	yes	+	+
Dysphagia	NA	NA	yes	yes	++	no	+++	+	+	+++	+	no
Slow ocular saccades	NA	NA	yes	NA	yes	yes	NA	yes	yes	yes	no	no
Paresthesia	NA	NA	no	NA	no	tingling sensation in feet	no	NA	burning sensation in hands and feet	NA	Pain in legs	tingling sensation in feet and pain in legs
Sensory impairment in extremities	yes	yes	yes	yes	yes	yes	NA	yes	yes	yes	no	no
Deep tendon reflexes	areflexia	areflexia	areflexia	NA	areflexia	areflexia	hyporeflexia	hyporeflexia	areflexia	areflexia	normal	hyporeflexia
Other symptoms/diagnoses	head tremor; dysphagia	transient fasciculations	painful cramps in left thigh	pronounced muscle wasting, paraparesis, reduced muscle strength and increased tone in arms	involuntary leg jerks and facial twitching	fasciculations	torticollis, hallucinations, absence-like episodes	mirror movements in hands^^4^^	inverted foot posture, involuntary facial twitching	profuse sweating, cough, excessive airway mucus production, involuntary facial twitching, atypical autism	hypotonic infant, hyperlaxity, myoclonic jerks, behavioral problems, everted foot posture	hyperlaxity, myoclonic jerks, attention deficit
Most recent ICARS score, max 100 (age)	NA	NA	43 (67)	NA	62.2 (66)	56.5 (57)	NA	37 (38)	57.5 (43)	21 (20)	NA	NA
Cerebellar atrophy (imaging modality, age)	+++ (CT, 76)	++ (CT, 72)	NA	+++ (MR, 60)	+++ (MR, 57)	++ (MR, 43)	++ (MR, 39)	+++ (MR, 32)	+ (MR, 33)	0 (MR, 20)	+++ (MR, 2)	++ (MR, 2)
Electroneurography	NA	NA	SN	NA	SN	S-MN	NA	SN	S-MN	S-MN	normal	NA
Orthostatic hypotension	NA	NA	NA	NA	fluctuating blood pressure	no	yes	yes	yes	yes	no	no
Bowel symptoms	no	no	NA	NA	constipation	bowel urgency	no	postprandial diarrhea	constipation	constipation	incontinence and constipation	constipation
Urinary symptoms, sexual dysfunction	urge incontinence		NA	erectile dysfunction	recurring UTI’s	no	UR	no	UR	UR	incontinence	incontinence
Unintended weight loss and BMI <18	NA	NA	NA	yes	yes	no	yes	yes	yes	yes	no	no
DNA-analysis	NA	NA	NA	NA	WGS	WGS	NA	NA	NA	WGS	WGS	WGS
Nr of repeat units Short-read, allele 1	NA	NA	NA	NA	52	42	NA	NA	NA	56	21	21
Nr of repeat units Short-read, allele 2	NA	NA	NA	NA	21	21	NA	NA	NA	18	21	21
Nr of repeat units Long-read, allele 1	NA	NA	NA	NA	57	NA	NA	NA	NA	74	–	–
Result: *ZFHX3* repeats	NA	NA	NA	NA	expanded	expanded	NA	NA	NA	expanded	normal	normal

More detailed clinical descriptions and information on additional family members are found in the supplemental note. Repeat lengths are provided as outlined in Figure 2. In addition, the following members of family 1 were analyzed genetically but are not shown in the Table: III:UFM carried 21 and 21 repeats. IV:UFM carried 18 and 21 repeats. The second unaffected (married-in) parent of V:I and V:II had 21 and 22 repeats. BD = balance disturbance; BMI = body mass index; CT = computed tomography; FBGC = familial basal ganglia calcification (see supplemental note); ICARS = international cooperative ataxia rating scale; ID = intellectual dysfunction; MRI = magnetic resonance imaging; NA = not assessed; SARA = scale for the assessment and rating of ataxia; SN = sensory neuropathy; S-MN = sensorimotor neuropathy; UR = urinary retention; UTI = urinary-tract infection; WES = whole exome sequencing; WGS = whole genome sequencing; ‘+’ = mild; ‘++’ = moderate; ‘+++’ = severe. Portions of the information in Tables 1 and 2 are reprinted, in modified and updated form, from [4] with permission from Elsevier.

### Screening of larger ataxia datasets and identification of additional families

Re-analyzing WES and WGS data from our ataxia series identified four additional individuals with ataxia from three additional families with expanded *ZFHX3* repeats, the probands of families 3–5, and individual family 3 IV:1 (Tables 1 and 2; Figure 1). When including the additional families, the genomic segment shared by all affected individuals shortened to 111 kbp; within this segment three very rare intronic SNVs were found exclusively in repeat expansion carriers, further defining a shared haplotype (Table S4). We did not find expanded alleles among persons with ataxia or controls in our in-house research database with individuals with other neurological diagnoses from southern Sweden, nor among 2,000 alleles in the SweGen database.

**Table 2:** Clinical phenotype and genetic results of affected members from families 2–5

	**Family 2**			**Family 3**		**Family 4**		**Family 5**	
**Individual**	**II:2**	**III:2**	**IV:1**	**III:1**	**IV:1**	**III:1**	**IV:1**	**II:1**	**III:1**
Age of onset (years)	43	38	25	57	44	45	43	50	40
Age at most recent evaluation/age at death	75	53	46	80	50	52	51	79	50
Initial symptom(s)	BD	BD, worsened fine motor skills	BD	BD	BD	BD	Gait and limb ataxia	BD	BD
Walking aid (most recent documented)	requires support	wheelchair	wheelchair	wheelchair	none	wheelchair	requires support	wheelchair	walking sticks
Dysarthria	yes	yes	+++	yes	yes	yes	++	yes	no
Dysphagia	no	no	no	yes	no	yes	++	yes	yes
Slow ocular saccades	NA	pathological saccades	yes	yes	yes	NA	yes	NA	NA
Paresthesia	no	no	NA	no	no	NA	no	no	yes
Sensory impairment in extremities	yes	yes	yes	yes	no	NA	yes	yes	yes
Deep tendon reflexes	areflexia	areflexia	areflexia	areflexia	areflexia	NA	hyporeflexia	areflexia	areflexia
Other symptoms/diagnoses	upgaze palsy, lower-back pain	increased muscle tone in legs at night	involuntary facial twitching, FBGC	restless legs	neuralgia, subjective cranial sensation	painful leg cramps	anxiety, painful leg cramps	–	flushes, restless legs
Most recent ICARS score, max 100 (age)	NA	NA	54,5 (46)	NA	23 (50)	NA	30 (50)	NA	NA
Most recent SARA score (age)	NA	NA	NA	NA	12 (50)	NA	NA	NA	NA
Cerebellar atrophy (imaging modality, age)	NA	++ (CT, 44)	++^a^ (MR, 37)	NA	++ (MRI, 49)	NA	++ (MRI, 43)	++ (CT, 51)	NA
Electroneurography	S N	NA	NA	S-MN	NA	NA	NA	S-M N	S N
Orthostatic hypotension	NA	no	yes	NA	no	NA	yes	yes	yes
Bowel symptoms	NA	bowel urgency and diarrhea	bowel urgency and diarrhea	constipation	no	NA	alternating constipation and diarrhea	–	constipation
Urinary symptoms	NA	UR and enuresis nocturna	no	yes	no	NA	urgency	UR	urgency
Unintended weight loss and BMI <18	NA	NA	yes	no	no	NA	yes	–	no
DNA-analysis	NA	NA	WGS	WGS	WGS	NA	WGS	NA	WGS
No. of repeat units Short-read sequencing allele 1	NA	NA	72	46	56	NA	56	NA	59
No. of repeat units Short-read sequencing allele 2	NA	NA	21	21	18	NA	21	NA	21
Result: *ZFHX3* repeats	NA	NA	expanded	expanded	expanded	NA	expanded	NA	expanded

a = CT showed calcifications in internal and external globus pallidus and in subcortical white matter; Please see also the legend to Table 1.

### Normal repeat length and locus architecture

**Figure 3:**
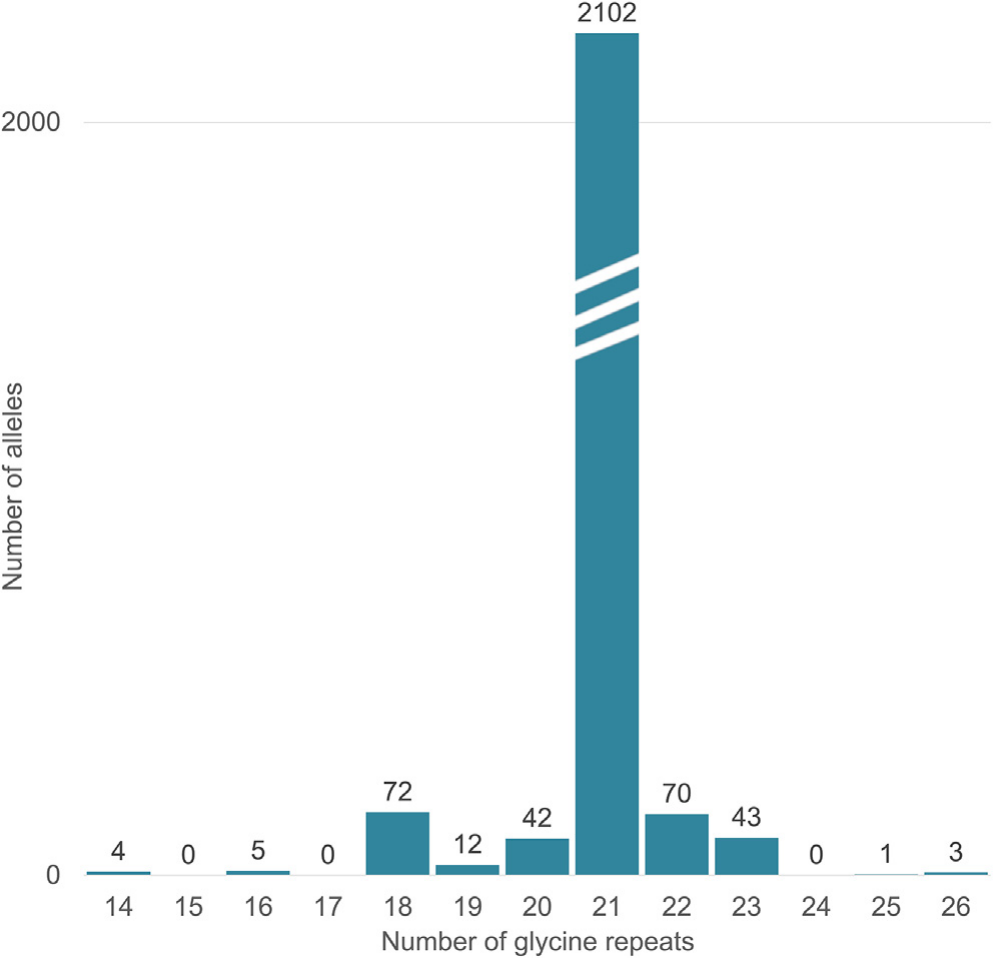
*ZFHX3* repeat lengths in non-expanded alleles Distribution of the total number of STR repeat units, including potential interruptions, as determined from the DNA sequence of 1,000 unaffected individuals from the Swedish SweGen WGS database,^20^ 150 in-house WES or WGS datasets without the *ZFHX3* repeat expansion, and 27 PacBio datasets from the Human PanGenome Reference Consortium.^21^ Numbers above the bars indicate the number of alleles. By contrast, expanded alleles in affected individuals (not shown above) were 42–72 GGC repeats in length, as determined from short-read WGS data, and 57 or 74 GGC repeats as determined by long-read sequencing of two affected individuals (see Table 1 and text), offering clear delineation between non-expanded and expanded alleles.

We used 300 non-expanded alleles from our in-house WES and WGS datasets, as well as 2,000 non-expanded alleles from the SweGen WGS dataset, to determine the normal *ZFHX3* repeat structure. All non-expanded alleles had interruptions within the GGC repeat; these interruptions were predominantly synonymous GGT and a non-synonymous AGT encoding serine (Figure 2; Table S5). The vast majority of alleles had the exact structure depicted in Figure 2. The remaining alleles deviated in multiple ways, as detailed in Table S5. The distribution of total repeat lengths is presented in Figure 3, both for the control datasets mentioned above as well as for 27 additional controls with long-read sequencing. In expanded alleles, there were no visible interruptions of any kind in short-read WGS data, but short reads might not suffice to entirely exclude interruptions because the repeat region is longer than the short read length of 150 bp. Long-read sequencing of two affected individuals confirmed a complete lack of interruptions: GGC was the only repeat unit.

### Genealogical data and clinical examination

We have collected clinical information on forty members affected by neurological disease in the five families (Figure 1). All five families originate from Skåne (approximately 1.4 million inhabitants), the southernmost region of Sweden, where our center is located. Fifteen affected members were examined by the authors of the previous^4^ and/or the present study. Tables 1 and 2 summarize the family members’ clinical phenotypes. Clinical descriptions are provided below and as a Supplemental Note. We also refer to our previous work^4^ for additional detailed clinical descriptions of families 1 and 2; these descriptions include videos of several affected family members.

Our genetic studies showed that family 1’s individuals V:1 and V2 did not share the repeat expansion that we found in all other genetically examined affected individuals from families 1 and 2. These two siblings, V:1 and V2, had signs and symptoms (see Supplemental Note) of a more severe neurodevelopmental disorder of hitherto undetermined genetic cause.

The remaining 38 affected members of families 1 to 5 shared clinical features; all had autosomal-dominant cerebellar ataxia with sensory and autonomic neuropathy. They developed gait or balance disturbance at a mean age at disease onset of 37.6 years, but with a range from 60 to 15 years in a pattern compatible with anticipation (Tables 1 and 2; Figure 1). Electroneurography showed signs of sensory or sensory-motor neuropathy; frequently, the sensory neuropathy signs were pronounced but motor findings relatively moderate. In all but two instances in our families, the affected child of an affected individual manifested disease symptoms earlier than the parent (Figure 1). The initial symptom was balance disturbance, commonly perceived as a tendency to stumble while walking. Symptoms progressed slowly but relentlessly, and all affected individuals had both gait ataxia and limb ataxia. Most of the examined adult family members with ataxia had lost the ability to write their name legibly with a pen or to stand without support. Slow horizontal saccades were seen in all individuals with *ZFHX3* repeat expansions, there was no nystagmus, and smooth pursuit eye movements were frequently unimpaired. When directing their gaze sideways, some of the affected individuals involuntarily turned their head in this direction and/or showed simultaneous involuntary perioral muscle activation. Mirror movements had been noted in one individual.^4^ Individuals who were younger at the onset of ataxia developed more severe additional symptomatology. Dysautonomia was a common sign. It mostly manifested with symptomatic orthostatic hypotension, which became very severe in members of the younger generations, and as difficulties with bowel or bladder control. Seven affected individuals with earlier onset had involuntary weight loss and/or muscle wasting accompanied by documented underweight body mass index (Tables 1 and 2) that in three of them became severe and was considered to have contributed to their deaths at ages 28–47 years. These individuals had not received feeding tubes and had dysphagia, and two of them died in the hospital after cardiac arrest. Other affected individuals had less severe swallowing problems and were able to maintain a higher BMI. Family members with severe symptomatic orthostatism received blood-pressure-elevating medication (fludrocortisone, etilefrine, droxidopa, midodrine) in combinations and dosages that changed over the years; one person was treated with erythropoietin with the aim of increasing blood volume and pressure; the effect of these measures varied. Individuals with diarrhea reported a benefit from loperamide tablets.

### Postmortem examination and neuropathology

**Figure 4:**
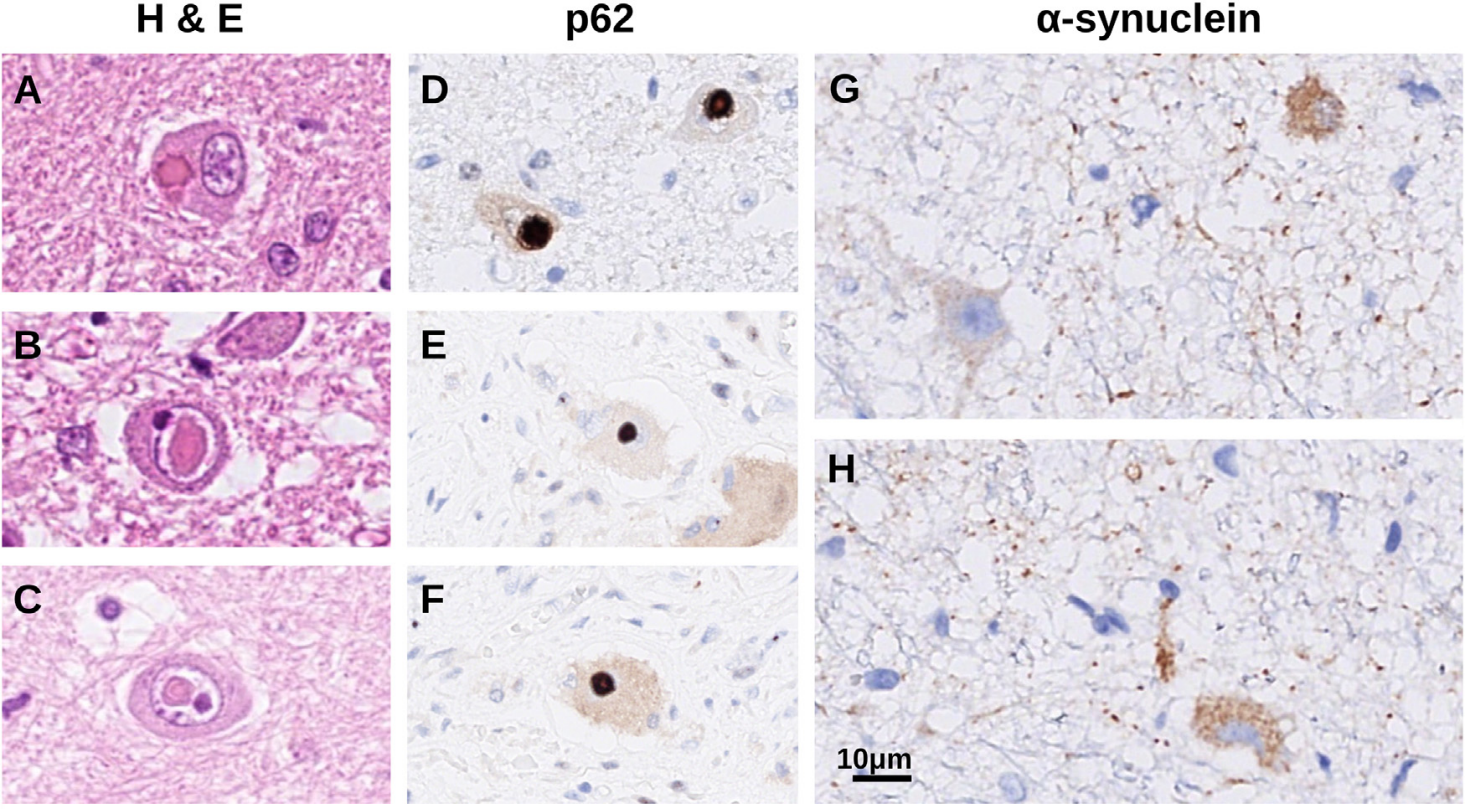
Neuropathology (A–C) Hematoxylin and eosin (H & E)-stained tissue from the medulla oblongata with eosinophilic, intracytoplasmic (A), and intranuclear (B and C) inclusions in neurons. (D) These inclusions stained positively with antibodies against p62. (E and F) Similar immunoreactivity against p62 was seen in inclusions in neurons of the esophageal myenteric plexus. (G and H) Alpha-synuclein-positive neurites and fine granular intracytoplasmic immunoreactivity in the medulla oblongata.

Individual IV:1 from family 1 had been treated with cefotaxime for urinary-tract infection for 11 days until 5 days prior to his death. He developed peritonitis after surgical PEG insertion, had a cardiac arrest, and was resuscitated but died the following day. Postmortem examination showed bilateral pneumonia with pulmonary edema and acute peritonitis. There was widespread invasive mycosis in most examined tissues, including the myocardium, lungs, gastrointestinal walls, skin, skeletal muscles, and brain. *Candida dublinensis* was identified in blood cultures after his death. Microscopic examination revealed intracellular inclusions in hepatocytes. The cerebrum exhibited mild atrophy, and there was mild cerebellar atrophy (see supplemental note). The substantia nigra appeared pale. Microscopic examination showed widespread foci of selective eosinophilic neuronal death attributed to the cardiac arrest and subsequent resuscitation.^22^ Signs of chronic disease included neuronal loss and gliosis in the cerebellum, brain stem, and spinal cord. A marked loss of pigmented cells was seen in the substantia nigra, and a moderate cell loss was seen in the locus ceruleus. The number of Purkinje cells was markedly reduced in the cerebellar hemispheres and moderately reduced in the vermis, and there was Bergmann gliosis. The dentate nucleus showed gliosis and a reduced number of cells; the remaining cells were atrophic and had perinuclear halos. There were abundant intranuclear and less abundant intracytoplasmic inclusions in brainstem neurons (Figures 4A–4C) and singular inclusions of the same types in the cerebral cortex. The inclusions were sharply delineated, and their aspect and distribution differed from the pathology of viral infections (CMV, HSV, rabies) that are known to be associated with intracellular inclusions; immunostaining against HSV was negative. There were no inclusions in cerebellar sections. Peripheral nerve fibers in the lower extremities showed marked thinning but with relatively preserved myelin, as in axonal death. Epicardial nerve fibers appeared normal in appearance. Skeletal muscle sections had normal appearance. Nerve cells of the myenteric plexus in the esophagus contained inclusions (Figures 4E and 4F). The inclusions stained for p62 (Figures 4D–4F). Alpha-synuclein immunoreactivity was seen in brainstem and medulla oblongata neurons (Figures 4G and 4H), in the hippocampus, and in gastrointestinal-tract myenteric ganglion cells, which also were atrophic. Mild alpha-synuclein immunoreactivity was seen in thin intradermal nerve fibers of the abdominal wall. Alpha synuclein immunopositivity was seen in neurites and in fine granular depositions within the cytoplasm of neurons, in different patterns than the p62-positive inclusions. There were no Lewy bodies.

## Discussion

We report the association of exonic GGC trinucleotide repeat expansions, encoding poly-glycine, in *ZFHX3* with autosomal-dominant cerebellar ataxia with sensory and autonomic neuropathy in five Swedish families, including two whose clinical phenotype we previously described in detail.^4^ Affected individuals developed gait and balance disturbances at variable ages, and the families displayed clear evidence for anticipation, which is compatible with a repeat expansion as its genetic cause. Key clinical symptoms include ataxia, neuropathy with predominantly sensory findings, slow saccades, and cerebellar atrophy on MRI and neuropathology. Orthostatic hypotension, loss of bladder or bowel control, or other signs and symptoms of dysautonomia occurred and severely impaired some of the affected individuals, especially those with earlier onset and more severe disease.

In data from large national and in-house cohorts, we have been able to define the normal length of the identified repeat to 14–26 trinucleotides encoding poly-glycine; the most common length by far was 21 repeat units, including interruptions. In short-read data, expanded repeats were estimated to be between 42 and 72 units of pure GGC. Short-read data are less accurate when a repeat expansion approaches the read length (approximately 150 base pairs or 50 trinucleotide repeat units), but non-expanded alleles could clearly be delineated in short-read data. A clear separation between the distribution of normal alleles (14–26 repeats) and pathogenic expansions (42–74 repeats) was seen. Two individuals were analyzed by both short-read and long-read sequencing; their repeat length was estimated to be 52 and 56 by short-read technology, but long-read sequencing revealed 57 and 74, respectively; this suggests that estimations based on short-read data do not accurately represent the true lengths of the expanded repeats in these two individuals. Although the anticipation observed in our families correlates well with the lengths observed in the long-read data, further research is needed to firmly establish a correlation between *ZFHX3* repeat length and age of onset or the severity of clinical symptoms. Long-read sequencing confirmed pure GCC repeats in these two expanded alleles. By contrast, all 2,300 non-expanded alleles that we assessed had one or more interruptions. Repeat interruptions are known to have a stabilizing effect on repeat length.^23^ We hypothesize that the interruptions stabilize the repeat length in unaffected individuals and that disappearance of interruptions might have been the first step toward gradual expansion of the repeat from generation to generation.^24^

All five families originated from the limited geographic area of Skåne, the southernmost region of Sweden, but the family trees could not be linked on the basis of existing genealogical information. Genetic data revealed that in all examined affected members of the five families, the *ZFHX3* repeat expansion was located within a shared genomic segment of at least 111 kbp in length, indicating a common founder event many generations ago. The oldest members of family 4 and 5 were not known to have neurological symptoms. This is compatible with the possibility that the actual founder event was spontaneous mutations that eliminated or reduced the number of repeat interruptions to an allele that then was prone to expand in length from generation to generation. However, we have not been able to identify any alleles without repeat interruptions but with normal repeat length.

Recent additions to the list of genetically defined hereditary ataxias include two repeat-expansion disorders that were detected through NGS: intronic biallelic complex pentanucleotide *RFC1* expansions causing cerebellar ataxia, neuropathy, and vestibular areflexia syndrome (CANVAS [MIM: 614575])^1^ and deep intronic monoallelic trinucleotide *FGF14* expansions causing late-onset spinocerebellar ataxia 27B (MIM: 620174).^2^ Both disorders were soon shown to be relatively common causes for previously undiagnosed hereditary ataxias.^1^^,^^25^ The fact that we identified five families with ZFHX3 repeat expansions from our center suggests a relatively high prevalence of this disorder, at least among persons with ataxia from southern Sweden. Future studies will, we hope, describe the prevalence of this disorder in other populations.^26^

Neuropathology revealed p62-positive intraneuronal inclusions, most of which were seen in the cells’ nuclei. A minority of nerve cells showed similar inclusions in the cytoplasm. Inclusions were seen in neurons of the central and peripheral nervous system, and in extraneuronal tissue. Pathological proteinaceous intranuclear or cytoplasmic depositions characterize many forms of spinocerebellar ataxia with poly-glutamine expansions; such forms include SCA1, 2, 3, 6, and 7.^27^^,^^28^ Exonic repeat expansions that encode poly-glycine have so far not been associated with human disease. However, expansions of CGG trinucleotide repeats in the 5′-untranslated regions of several genes have been postulated to cause disease through specific mechanisms that result in the translation of poly-glycine.^29^ These include fragile X tremor/ataxia syndrome (MIM: 300623),^30^ an adult-onset disorder involving ataxia, and neuronal intranuclear inclusion disease (MIM: 603472).^31^^,^^32^ In a large series, 39% of individuals with the latter disease had ataxia, and 92% had autonomic dysfunction.^33^

*ZFHX3* is located between the markers D16S3019 and D16S512 on the long arm of chromosome 16, to which spinocerebellar ataxia type 4 (SCA4) had been mapped by conventional linkage analyses in a German and in two Swedish kindreds reported from Stockholm, Eastern Sweden.^34^^,^^35^ The gene is in relative proximity to the locus reported in the original SCA4 kindred from Utah.^36^ The relatively unique clinical phenotype in the families in the present study corresponds well with the phenotype described in SCA4, although we assume that examination and reporting methods have changed since the earlier descriptions of SCA4. Furthermore, the original Utah SCA4 kindred originated from Sweden.^36^^,^^37^ We therefore suggest *ZFHX3* expansions to be the cause for SCA4.

There is one previous neuropathological report of a member of the large German SCA4 kindred. This man had disease signs since age 55, developed sensory loss, areflexia, and neurophysiological signs of axonal neuropathy; became wheelchair dependent; and died at age 70 years. No intracellular inclusions were reported, but staining for poly-glutamine aggregates was performed and was negative, and there was cell loss in the brain stem nuclei, cerebellum, and spinal cord.^38^ The individual who was autopsied within our study had much younger onset and clinically more severe disease, and we hypothesize that poly-glycine inclusions might only be formed, or be formed more abundantly, in individuals who have early onset and who may have longer repeat expansions in *ZFHX3*.

Involving data from two large kindreds and three additional families, our study provides strong evidence on the genetic association of repeat expansions in *ZFHX3* with this specific clinical phenotype. Analyses of more than 2,300 non-expanded alleles revealed that normal alleles are considerably shorter than the read length of short-read NGS sequencing methods, and we have been able to correctly identify four additional individuals from three families through analyses of short-read data. Our study remains limited by the fact that we so far have been unable to determine the repeat expansions’ exact lengths in all affected family members or to screen additional unaffected members to confirm that they have normal repeat lengths, for example by an orthogonal method such as repeat-primed PCR. Such analyses from all family members with this disorder would also be necessary to validate the presumed inverse association of repeat length with age at symptom onset, as we have observed in long-read sequencing data from only two individuals.

We also have not been able to proceed to experiments that elucidate the functional effect of the expanded *ZFHX3* trinucleotide repeat on transcription or translation, or the effect of an expanded poly-glycine repeat on protein function. It is possible that the eosinophilic inclusions seen in the affected individual we report on here are composed of mutant ZFHX3 with longer-than-normal stretches of poly-glycine and without a serine interruption and that such mutant ZFHX3 proteins exert toxic effects. Other possible pathomechanisms include RNA toxicity, whereby transcripts with expanded uninterrupted repeats sequester RNA-binding proteins and form RNA foci.^29^ Previous work has shown that ZFHX3 plays a role in neuronal differentiation^39^^,^^40^^,^^41^^,^^42^ and that ZFHX3 is implied in cerebellar neurons’ responses to oxidative stress,^43^ plausibly linking variation in this gene to a cerebellar disorder. Furthermore, *ZFHX3* was identified as one of a few candidate genes for sporadic Hirschsprung disease,^44^ which might connect *ZFHX3* to the pathology observed in the enteric nervous system and the clinical symptoms of diarrhea and fecal incontinence.

Unveiling the genetic cause of more and more cerebellar ataxias has made it possible to start developing targeted treatments.^45^ We hope that our clinical and genetic observations might lay the ground for research on pathogenesis and pathomechanisms and ultimately help to develop treatment for individuals with this severely disabling disorder.

## Data and code availability

Catalogs used for querying ExpansionHunter for the pathogenic *ZFHX3* repeat expansions, generated during this study, are provided in the supplement. There are restrictions to the availability of individual NGS datasets because of human subjects’ confidentiality, national laws, regulations, and institutional practices; access to individual NGS datasets usually requires approval from the Swedish Ethical Review Authority.

## Supplemental information

Document S1. Figures S1–S15 and Tables S1–S5

Document S2. Article plus supplemental information
