# Supplementary material for: Exonic trinucleotide repeat expansions in ZFHX3 cause spinocerebellar ataxia type 4: A poly-glycine disease
Source: Am J Hum Genet. 2023 Nov 29;111(1):82–95. doi: 10.1016/j.ajhg.2023.11.008 (PMC10806739; doi:10.1016/j.ajhg.2023.11.008)
Supplement: Document S1. Figures S1–S15 and Tables S1–S5 [file mmc1.pdf]

The American Journal of Human Genetics, Volume *111*

Supplemental information

Exonic trinucleotide repeat expansions in *ZFHX3* cause spinocerebellar ataxia type 4: A poly-glycine disease

Joel Wallenius, Efthymia Kafantari, Emma Jhaveri, Sorina Gorcenco, Adam Ameur, Christin Karremo, Sigurd Dobloug, Kristina Karrman, Tom de Koning, Andreea Ilinca, Maria Landqvist Waldö, Andreas Arvidsson, Staffan Persson, Elisabet Englund, Hans Ehrencrona, and Andreas Puschmann

Supplemental Note: Case Reports

Our previous publication[1] contained detailed information on these and additional members of both families as they were available at the time of that work, that were presented in the main text table and in the supplement esupp2.[1] The below pedigree illustrates which family members were described or examined in which of our two publications and indicates changes in numbering:

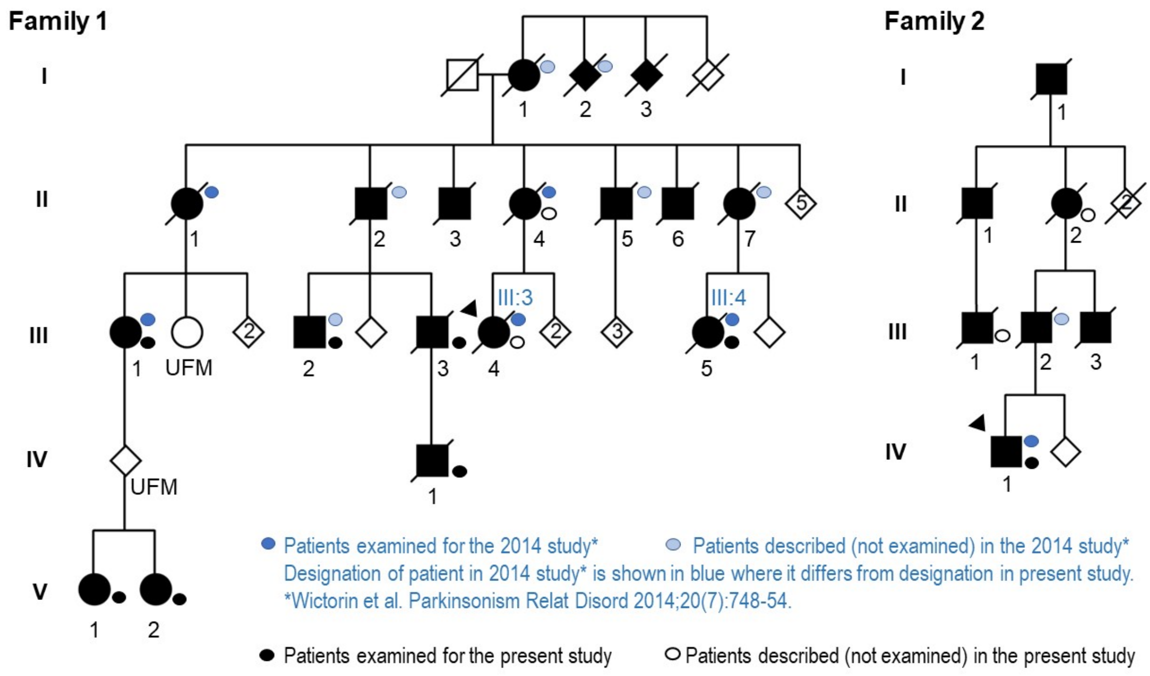

Case reports on the following pages summarize the clinical picture of affected individuals not previously published, those who were followed up in 2022-2023 and/or those for whom relevant new information had emerged since the original publication.

Family 1

II:4 Since the previous study, this individual had developed dysphagia and painful cramps in her left thigh. She also had several hospital admissions due to infections. She died 75 years old.

III:1 This family member was examined at the age of 66 years. Since the previous study this individual had started using a wheelchair and required turning devices to move between bed and wheelchair. BMI had fluctuated between 13.9 and 18.8 during the previous years. During the past year the individual had begun to choke while drinking and was referred to a speech therapist who confirmed dysphagia. She medicated for chronic constipation and hypertension and required a urinary catheter. The individual described episodes of involuntary jerky movements in the legs which had begun at age 63.

III:2 The existence of this affected family member was known to the research group in 2014, but he had previously not been available for physical examination. When examined for the first time in this study, he was 57 years old and had been using a wheelchair for five years. He had sensory impairment of all modalities in mainly the lower extremities with distal to proximal gradient, areflexia, intention tremor and fasciculations. Fasciculations were especially noticeable above his left knee. He experienced tingling sensations in his feet at night. The individual reported problems with bowel urgency but denied urinary symptoms and dizziness. The individual had medically treated hypertension.

III:3 This family member was not described in the previous study. He was had evaluated and followed at our neurology clinic but we had been unaware of his relationship to this kindred until after 2014. He was diagnosed with torticollis at the age of 25 which improved by age 45. At the age of 35, he was described as having an atactic gait and atactic limb movements along with diminished reflexes and positive Romberg's test. Reportedly he also had urinary retention. From the onset of ataxic symptoms, BMI varied between 12.2 to 17.4. At age 45, he refrained from using a walking aid despite incidents of falling each week. The following year he had hallucinations and several absence-like episodes with unclear etiology. He also started using a wheelchair and had developed severe ataxia, dysphagia, dysarthria and orthostatic hypotension. He had several hospital admissions due to repeated infections and died from sepsis and the age of 47 years.

III:4 ***(designated III:3 in previous publication[1])*** Since the previous study, this affected family member had received intensive therapy for her severe symptomatic hypotension/syncope, including midodrine, droxidopa and intermittent erythropoietin infusions. Despite these medications, she first used an office chair on rolls to move around in her home, as she would frequently faint when trying to stand up or walk. Later she resorted to a life near the floors, moving on all fours as her blood pressure also had become too low when sitting on a chair. She lost weight but declined a feeding tube. She died 42 years old.

III:5 ***(designated III:4 in previous publication[1])*** When this family member was examined at the age of 43, she could no longer stand without strong support of both arms and required support from another person when moving from wheelchair to bed. This was due to the progression of both ataxia and muscle wasting. A slightly inverted foot posture was noted at rest. She had developed mild dysphagia and a lack of appetite. BMI had varied between 11.7 and 14.4 during the previous year. She described a burning sensation in both hands and feet

both day and night, relieved slightly by gabapentin, an analgesic for neuropathic pain. There was sensory impairment of all tested modalities not only in her legs but also arms, with distal to proximal gradient. Family members described she had begun having episodes of sleep terror each night. She had also developed an inability to close her eyes properly for which she had received titanium weight implants in her eyelids. She lost more and more weight, declined a feeding tube, and died at age 44 years, weighing only 30 kg (BMI 11,7 kg/m^2), from marasmus and cardiac arrest.

IV:1 This individual was not described in the previous study because he had only just become 18 years old and since we were unaware of his connection to the family. He was diagnosed with atypical autism and reportedly had temper tantrums, panic attacks, and mild difficulties with verbal communication, concentration, and learning since childhood. Attention deficit hyperactivity disorder was mentioned in several medical records, but it was unclear whether this had been diagnosed on the basis of formal criteria. Balance disturbance was first noticed at the age of 15 years. By the age of 20, he had developed areflexia and problems with fine motor skills. Ataxia symptoms progressed and at the age of 23 he had mild dysarthria, wide-based and slowed gait, and reduced hand-foot coordination, but was still able to ride a bicycle. At 24, he had orthostatic hypotension and began sweating profusely during low-intensity physical activity, for example shorter walks and moving from bed to chair. The subsequent years he developed severe dysphagia and involuntary facial twitching, and had excessive airway mucus production and difficulty to expectorate. At 26, he required a walker at home and a wheelchair for longer distances. He had sensory impairment of all modalities in upper and lower extremities. He required clean intermittent urinary catheterization for several years and had recurring constipation. BMI during his last two years had decreased gradually from 20.4 to 15. He had percutaneous endoscopic gastrostomy performed at age 28 years but died the day after the gastrostomy from cardiac arrest and signs of severe infection. Postmortem examination revealed invasive mycosis with miliary spread to all examined inner organs, CNS, skin, and musculature. There was necrosis of gut, spleen, and liver.

V:1 (does not have the *ZFHX3* repeat expansion) This child was born after the data collection for the previous study. As an infant she was hypotonic and showed delayed psychomotor development, especially of her ability to walk. Her parents noticed uncoordinated movements when she was around 6 months old. According to her parents she was unable to stand on her own at 6 years of age. When examined at the age of 8 years, she was able to stand but still required the support of one hand. She used a walker, had a wide-based gait and everted feet. She preferred to move around by pushing herself forward on her knees but reportedly fell several times a day even from this position. She reportedly had a high pain threshold but occasionally experienced a painful sensation in her legs. According to her parents, she often choked while drinking. Her balance and coordination problems reportedly fluctuated over time. Myoclonic jerks were noticed in the hands during rest. Both somatic sensation and reflexes appeared normal. A general lack of subcutaneous fat, prominent superficial veins and joint hypermobility were observed. Several cognitive symptoms were described in her medical records and by her parents. The girl had an underdeveloped use of language, a mild intellectual disability, a strong need for daily routines, and was very selective about food, only eating certain food items. Her parents also reported severe behavioral problems with an inability to control emotion. She had intermittent constipation and had required a diaper since birth due to fecal and urinary incontinence. It was unclear whether the incontinence was of organic origin or due to cognitive impairment. She received antidepressants and sleep medication. When the child was examined, neither of the

parents showed any signs of the disease; the parent belonging to Family 1 (**IV:UFM** in Figure 1) was 37 years old at the time and was shown to have two non-expanded *ZFHX3* alleles.

V:2 (does not carry a *ZFHX3* repeat expansions) This child was born after 2014. No abnormality of normal muscle tone was noted in her infancy and early psychomotor development was normal, except for delayed gait. Her parents noticed unusual gait pattern and motor dyscoordination at 1.5 years of age. Six months prior to our examination the girl had begun to report intermittent tingling in her feet and occasional pain in her legs. The parents described that the girl's symptoms fluctuated over time; on bad days she was unable to climb stairs and was "shakier". When examined at the age of 4 years, she was able to walk without support, but preferred to run to keep a better balance. She was able to stand with both feet together and eyes open but could not stand in tandem. She was able to walk on her toes but not on her heels. Myoclonic jerks in the hands were noticed during rest. Somatic sensation appeared normal, but reflexes were diminished. The parents described that the daughter had once had an hour-long episode of cramping while remaining conscious, and an episode of fever preceded by turning blue around the mouth, both lacking obvious explanations. Toilet training was successful for a year, but at age 4 the girl had gone back to using a diaper because of urinary incontinence. She had constipation problems since birth. According to the parents, the girl had fallen behind in preschool and showed an apparent attention deficit. Neuropsychiatric investigation was pending.

Family 2

II:2 This individual was not described in the previous study because medical records had not been available. She developed a balance disturbance at the age of 43. The same year, neurologist’s examination showed atactic gait, areflexia, and positive Babinski’s sign bilaterally. Eye examination showed up-gaze palsy. Ten years later, she occasionally needed to support herself against a wall while walking, and fine motor skills had worsened, but she remained able to carry out regular housework chores. She had a sensory impairment of all modalities and mild dysmetria. She was troubled by pain in the lower back. She was described as dissimulating and anxious. She died 75 years old. According to notes in relatives’ medical records, II:2’s father I:1 had had balance disturbance for at least 10 years prior to his death at the age of 70.

III:1 This family member was briefly described in the previous study, but more detailed information has become available from medical records. At the age of 38, he noticed a difficulty to walk straight, especially in the dark, along with worsened fine motor skills. At 44 years, he had developed wide-based gait, dysarthria, dysmetria, and areflexia except for a weak triceps reflex response on both sides. Three years later, documentation described pathological saccadic ocular movement but without further specification. At 49, he had started using a walker. He fractured his femur at age 52, and thereafter used a wheelchair, but he was still able to move between bed and chair without support. He developed sensory impairment of all modalities in upper and lower extremities, complete areflexia, and positive Babinski’s sign bilaterally. He was prescribed diazepam because of increased muscle tone in the legs at night. He also had medically treated hypertension. He had frequent bowel problems, both constipation and diarrhea. During his last years he obtained a long-term catheter due to urinary retention and nocturnal enuresis. He died 53 years old.

III:2 This individual has been described in our previous work. According to family members, this person had a brother (**III:3**) with similar neurological symptoms which began at the age of 20; **III:3** died at the age of 40 years. Medical records could not be retrieved due to missing personal data.

IV:1 Examination of this person at the age of 46 showed that ataxia and dysarthria had progressed since our previous study. He had been using a wheelchair for the past 7 years, and sensory impairment was present more proximally in the lower extremities than before. As in the previous study, he experienced no problems with dizziness but still struggled from bowel urgency and chronic diarrhea assessed as having a neurologic cause. Two years prior to this study, he had been hospitalized due to poor health status with BMI 16.6. At the time of our most recent examination, he had BMI 22.7 and muscle mass was well preserved in the upper body. He described a good appetite and no dysphagia. He was prescribed buspirone at his own request as he had heard the medication could mitigate ataxia. Despite lack of formal evidence for this indication, he felt the medication had a positive effect on overall wellbeing.
Clinical genetic testing revealed a novel truncating variant *SLC20A2* (NM_001257181.1) c.1240G>T, p.(Glu414*), interpreted as ”likely pathogenic” because of the known association of other truncating variants in this gene with primary basal ganglia calcification type 1 (MIM 213600).[2] On CT, there were mild calcifications in basal ganglia and cortical areas (below), in typical distribution for this genetic type of basal ganglia calcification *SLC20A2* gene, sparing the cerebellum. However, in a large case series, only 8% of patients with *SLC20A2*-related disease had ataxia, and penetrance regarding clinical symptoms is known to be incomplete

even in carriers of other *SLC20A2* variants who show intracerebral calcifications on CT scans.[3] No other examined member of Family 1 or 2 carried this variant. We thus considered the variant a cause of the calcifications but of minor or no relevance for this individual's clinical phenotype.

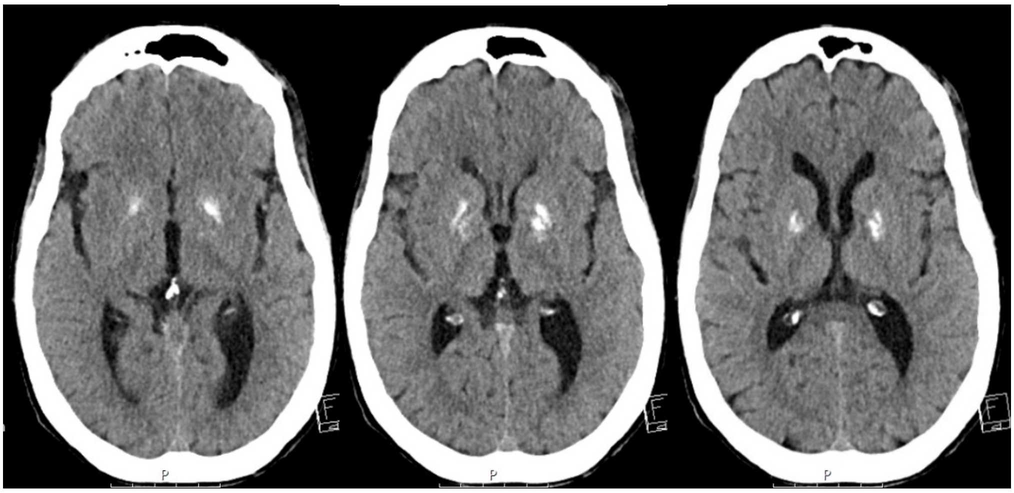

Family 3

According to information from the family, **I:1** had become unable to walk at higher age. **II:1** had balance problems starting in her 50s and died at age 68 years. **II:2** had very similar problems starting in his 50s. **III:2** had similar symptoms.

III:1 This male individual developed balance problems at age 57 years that had been progressive. He used a walker since age 63. On examination he had hearing loss, had slow and hypometric horizontal saccades, and rather turned his head than moved his eyes to look sidewards. He had mild cerebellar dysarthria. Sensibility for pain, vibration, proprioception, cold and light touch was decreased distally in legs and arms and no tendon reflexes could be elicited. Heel-knee-slide showed marked dysmetria and decomposition of movement, and there was mild to moderate ataxia in finger-nose test and dysdiadochokinesis in fast alternating hand movements. Nerve conduction studies at age 70 showed complete lack of sensory responses in median, ulnar and sural nerves bilaterally, and moderately decreased amplitudes in lower extremity motor nerves. An MRI of the brain at age 78 showed cerebellar atrophy:

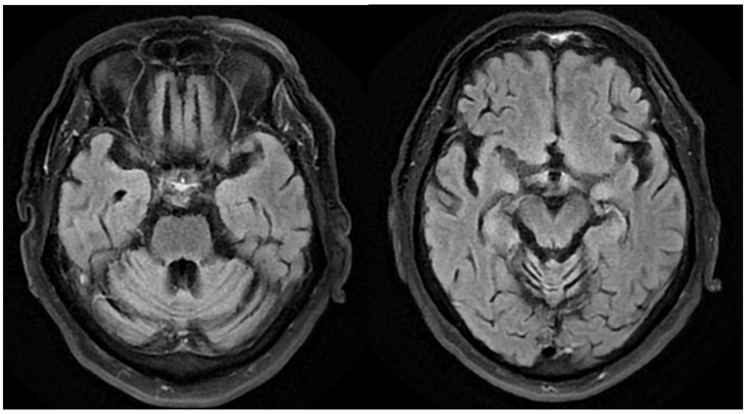

IV:1 **III:1**’s son had started to notice balance problems from age 44 years. When first seen at our department at age 47, there was ataxia of gait and in extremities and mild dystonic head movements. He was able to walk and perform physical work at 50 years of age, but by then had broad-based gait, nasal dysarthria. MRI showed mild cerebellar atrophy, especially of the anterior cerebellar lobe:

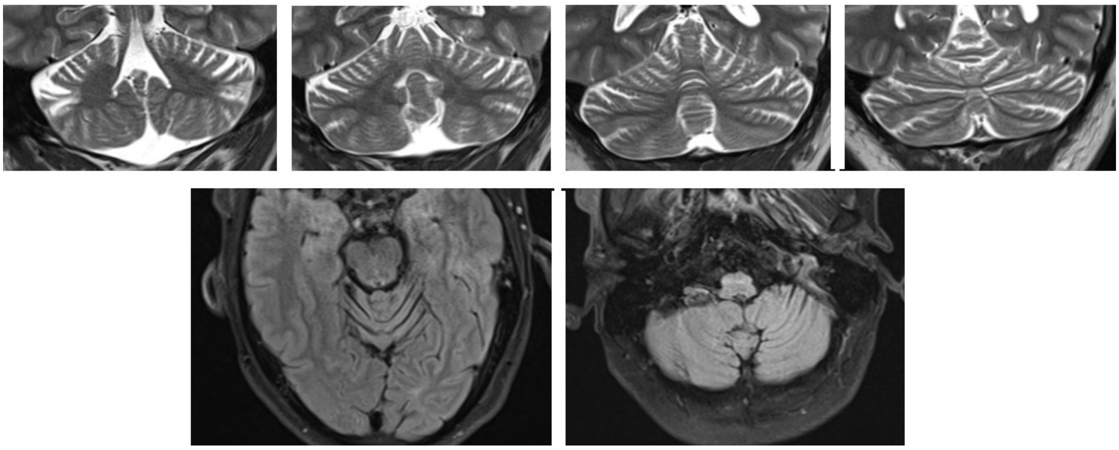

Family 4

IV:1 This female proband developed a balance disturbance at age 43. When examined within our research study, she had nasal speech, slightly impaired balance with signs of ataxia in upper and lower limbs and slight weakness distally in the lower limbs, difficulty walking on heels and elevating the front foot from the ground while standing. Reflexes were weak and vibration sense reduced in lower limbs. She reported nocturnal muscle cramps in the legs, in abdominal muscles, and bruxism. Tilt table test showed both immediate and delayed falls in blood pressure.

Seven relatives with similar symptoms were reported in the family (Figure 1).

The proband's grandmother (**II**, grey symbol in Figure 1) had gait problems, but details have been difficult to reconstruct; she died at age 64.

III:1 developed balance impairment at approximately 45 years of age. Subsequently, she had difficulties walking, at age 48 she developed severe leg pain and muscle cramps. During her last years of life, she had been unable to walk and needed much help with daily life activities such as eating, dressing and personal hygiene. She died at age 52.

Two maternal aunts of the proband, **III:2** and **III:3** had similar symptoms starting in their 40s and 50s, both eventually required a wheelchair. They died at 58 and 67 years of age.

Their three children, cousins to the proband, were all affected, with symptom onset at an earlier age:

IV:2 developed symptoms of impaired gait in adolescence and needed to use a walker early in the disease progression; she died at age 50.

IV:3 and **IV:4** developed symptoms of balance impairment at 29 and 30 years of age and died in their thirties after approximately 5 years disease duration. One of them had difficulties sitting or walking that were ascribed to severe back pain. According to information from the proband, the other male cousin preferably sat on the floor and moved around on the floor, without trying to stand up or use a wheelchair, as his disease had progressed. (This reminded us of what we saw in Family 1, **III:4**, where this was due to severe orthostatism.)

Family 5

According to family history, neither parent of **II:1** had gait or balance problems nor any other signs of neurological disease when they died at 79 and 86 years of age.

II:1 This male individual was followed at our department for over ten years; abundant medical records at our department were reviewed within this study, and additional information was received from his daughter. At about 50 years of age, he developed gait and balance disturbance that had progressed. A CT scan of the brain at 51 years of age showed moderate cerebellar atrophy, which was ascribed to a neurotoxic effect of solvents at the his workplace after evaluation at an occupational health department. He stopped working because of his disability at age 56. He needed to use a wheelchair since age 66. Neurologic examination at 67 years of age showed ataxic gait and limb ataxia. There was predominantly distal weakness in upper and lower extremities, muscle atrophy and some myokymia in these muscles. Sensation for the tested modalities pain, light touch and vibration was lost distally in upper and lower extremities. No tendon reflexes could be elicited. Nerve conduction studies at age 68 showed an axonal sensorimotor polyneuropathy, with predominant sensory findings and absence of all sensory responses. With increasing age, gait disturbance progressed, he lost ambulation, and developed dysarthria and dysphagia. Symptoms and signs of orthostatic hypotension were recorded but this was not assessed. Fasciculations in extremities and tongue were noted. A clinical diagnosis of hereditary sensorimotor neuropathy type 2 was entertained but no genetic testing had been performed. He died in the hospital at 79 years of age from respiratory failure after aspiration pneumonia that was aggravated by chronic obstructive pulmonary disease. This individual had no siblings.

III:1 This female family member had progressive gait disturbance with balance problems since about 40 years of age, which has remained the dominating symptom. She also experienced dysesthesias in lower extremities, mild subjective dysphagia and restless legs symptoms. Urinary urgency, constipation and dizziness when standing up also occurred. Nerve conduction studies showed absence of sensory responses in all nerves examined. Orthostatic test verified orthostatic hypotension on several occasions. At the time of referral, she used walking sticks. Neurological examination at our center at 50 years of age showed ataxic gait and immediate loss of balance on Romberg’s test. She had ataxia during heel-knee-shin-slide that improved when she was asked to look at her movements. These findings were interpreted as sensory ataxia. However, her MRI at age 51 years showed moderate cerebellar atrophy. She had severe loss of vibration sensation in upper and lower extremities, milder distal loss of touch and pain sensation in the lower extremities and areflexia. No dysarthria, eye movement disturbance, muscle atrophy, fasciculations, weakness or upper motor neuron signs were found.

Supplemental Figures and Legends

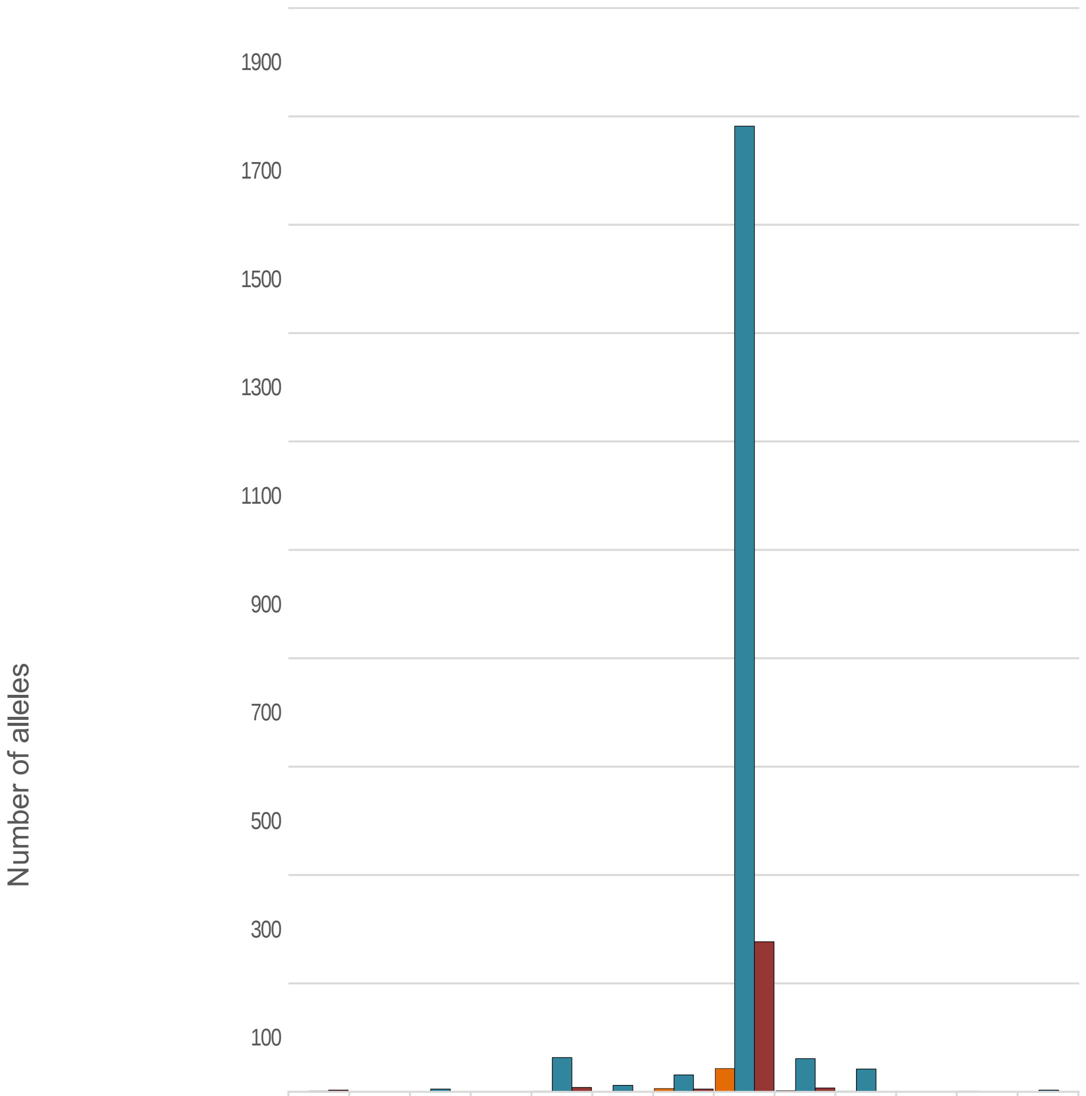

	14	15	16	17	18	19	20	21	22	23	24	25	26
27 Long Read controls	0	0	0	0	1	0	6	43	2	1	0	1	0
SweGene	1	0	5	0	63	12	31	1782	61	42	0	0	3
In-house dataset	3	0	0	0	8	0	5	277	7	0	0	0	0

Number of glycine repeats

Figure S1: Distribution of non-expanded alleles (details)
The distribution of total repeat length, including interruptions, in 2,000 non-expanded alleles from the SweGen database (blue), 300 alleles of our in-house NGS datasets (red), and 52 long-read alleles from the Human Pangenome Reference Consortium (orange). Below the diagram are the exact counts of allele lengths. It is based on short-read WGS and WES data, as well as long-read WGS data.

Figures S2-S15: Sequencing images

The images in this section were all produced with REViewer (short-read data, Figures S2-S13) or TRVZ (long-read data, Figures S14 and S15; https://doi.org/10.1101/2023.05.12.540470). For each individual, the normal allele is shown first, above the expanded allele. Sequence is depicted along the positive reference strand, so that, for example, GGC is shown as CCG, and *ZFHX3* transcription runs from right to left. In the REViewer images, flanking and AGT interruption sequence is colored blue, GGY sequence downstream of the AGT interruption is colored orange, GGY sequence upstream of the AGT interruption is colored green. In the TRVZ images, flanking sequence is colored green, while GGC sequence is colored blue, and any mismatches owing to interruptions are colored grey.

Short-read data with presupposed AGT interruption (Figures S2-S9)

Genotyping with a presupposed AGT interruption was successful for normal alleles, where the AGT unit is present. However, the expanded alleles showed no sign of interruptions – the reads were thus forced to map to either side of the reference AGT interruption, causing a failure of the total length estimation.

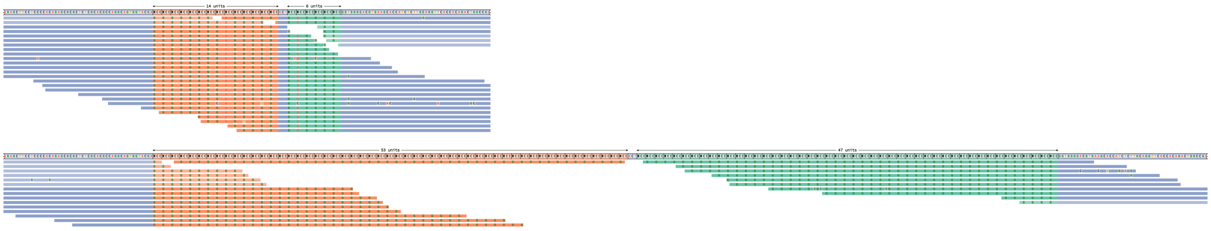

Figure S2: Family 1 III:1.

A canonical non-expanded allele, and an expanded allele. The true length of the expanded allele was determined with long-read sequencing to 57 GGC units, for comparison.

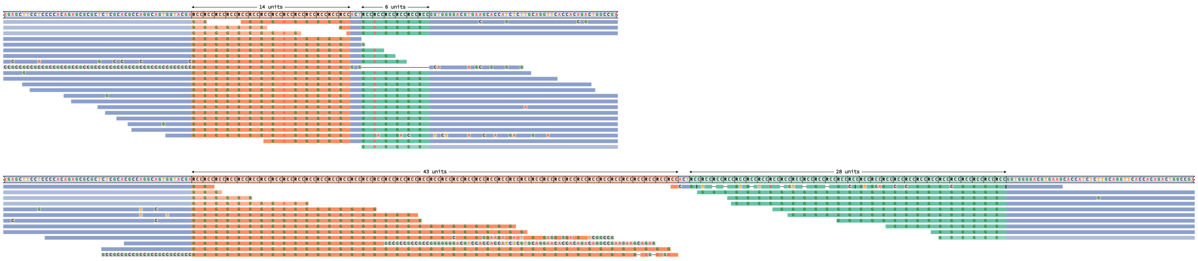

Figure S3: Family 1 III:2.

A canonical non-expanded allele, and an expanded allele.

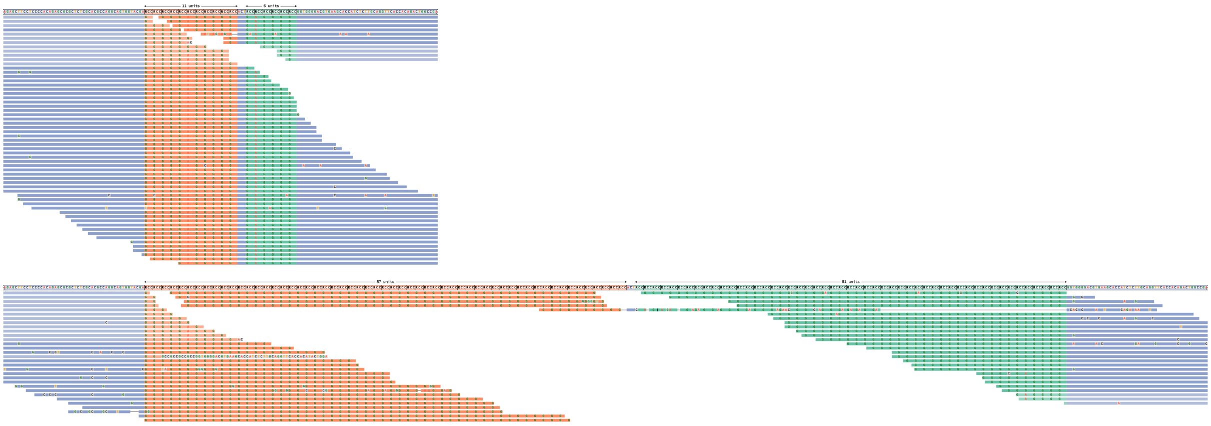

Figure S4: Family 1 IV:1

A non-expanded allele three units shorter than usual, and an expanded allele. Read depth is remarkably better than in other individuals. The length of this expanded allele was determined to 74 with long-read sequencing, for comparison.

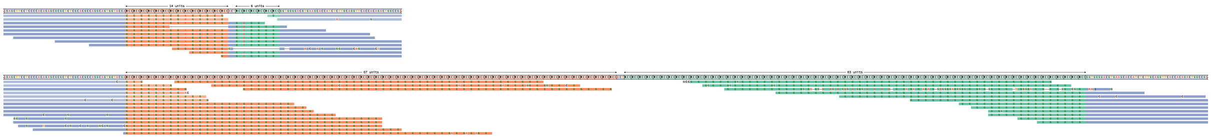

Figure S5: Family 2 IV:1

A canonical non-expanded allele, and an expanded allele.

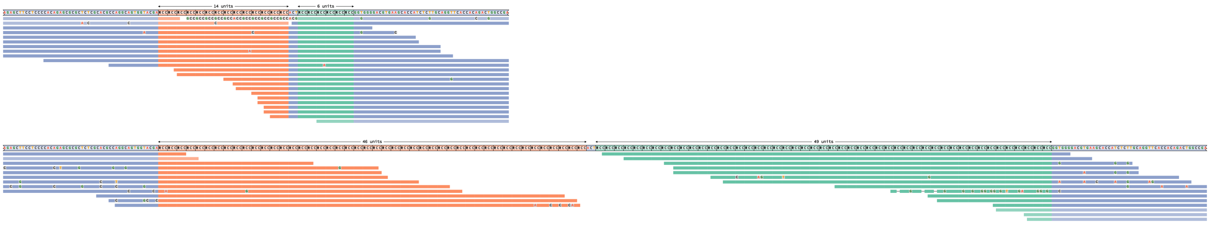

Figure S6: Family 3 III:1

A canonical non-expanded allele, and an expanded allele.

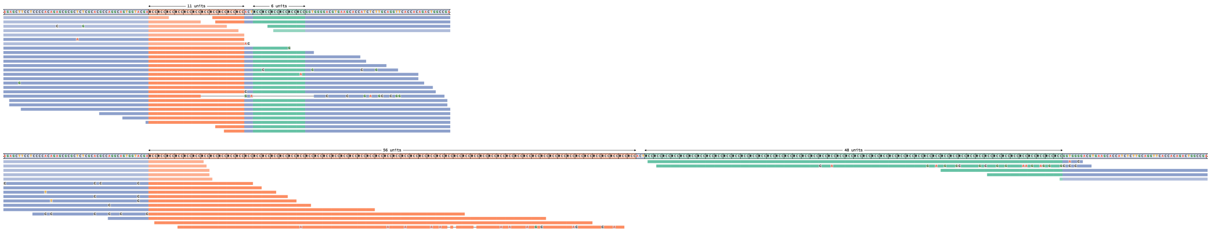

Figure S7: Family 3 IV:1

A non-expanded allele three units shorter than usual, and an expanded allele.

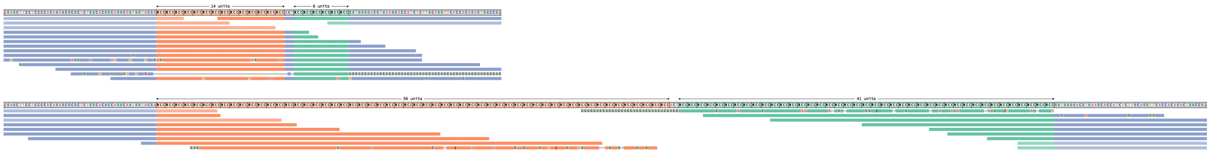

Figure S8: Family 4 IV:1

A canonical non-expanded allele, and an expanded allele

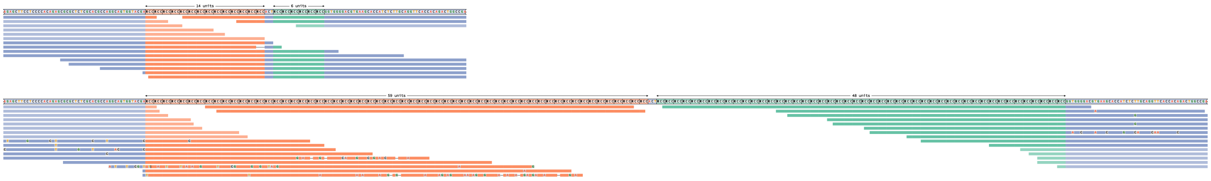

Figure S9: Family 5 III

A canonical non-expanded allele, and an expanded allele.

Short-read data without presupposed AGT interruption (Figures S10-S13)

Genotyping without presupposing the common AGT interruption results in better length estimates for alleles that do not have the AGT interruption. For expanded alleles, the genotyped length is an estimate, or minimum length. For non-expanded alleles, the genotyped length is exact regardless of whether there are AGT units present or not. If present, the AGT interruption shows up as sequence mismatch.

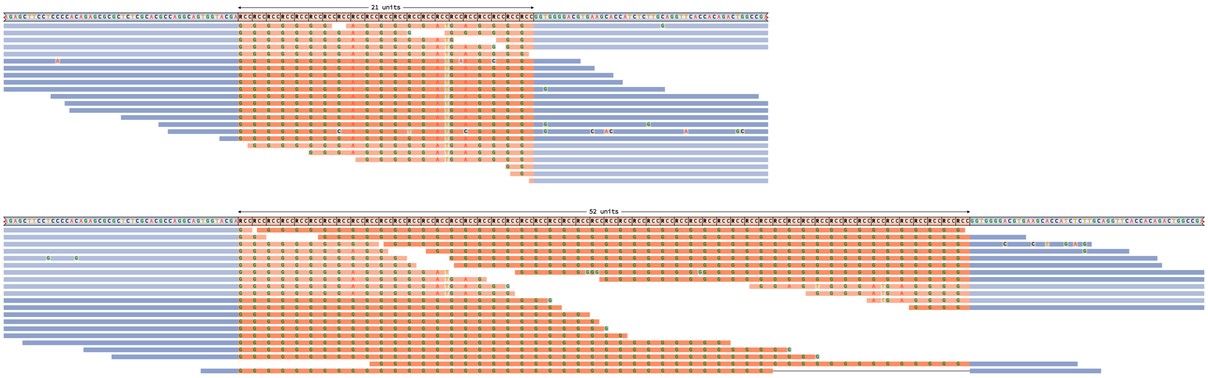

Figure S10: Family 1 III:1

A canonical non-expanded allele, and an expanded allele. The length estimate of the expanded allele of 52 units was close to the 54 units detected by long-read sequencing of this individual.

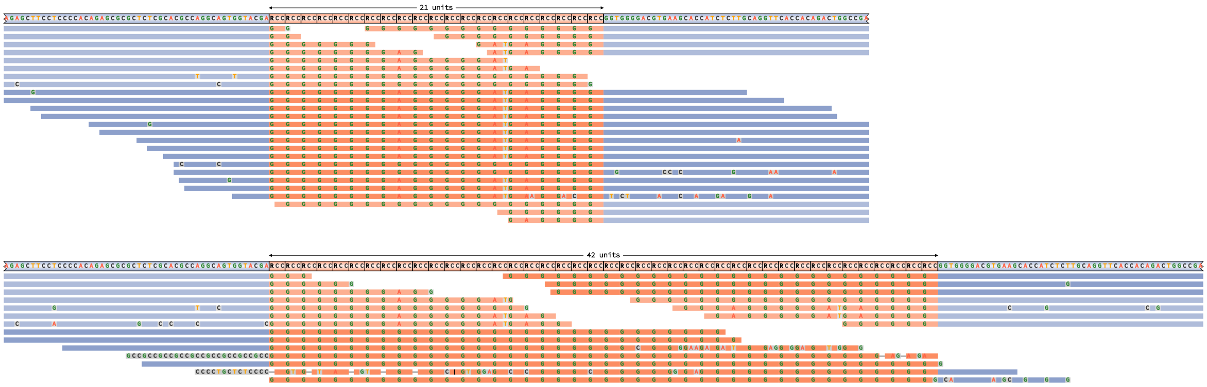

Figure S11: Family 1 III:2

A canonical non-expanded allele, and an expanded allele.

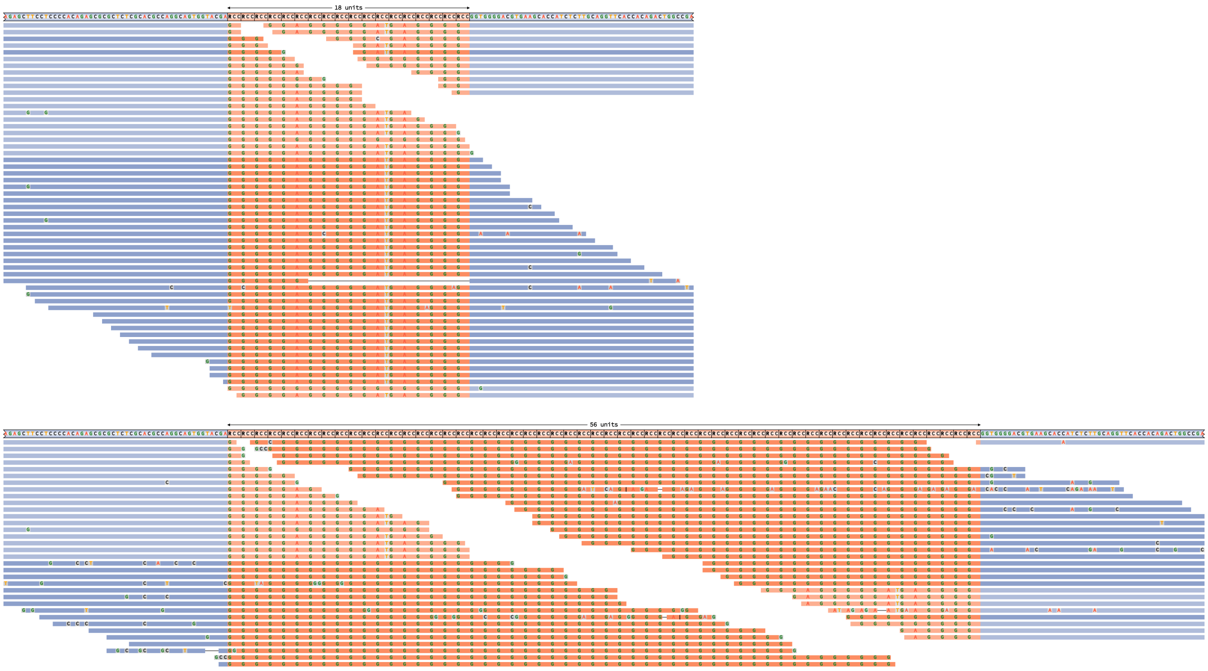

Figure S12: Family 1 IV:1

A non-expanded allele three units shorter than usual, and an expanded allele. The length estimate using short-read sequencing was 56 units, underestimating the length of 74 units as disclosed by long-read sequencing of this individual.

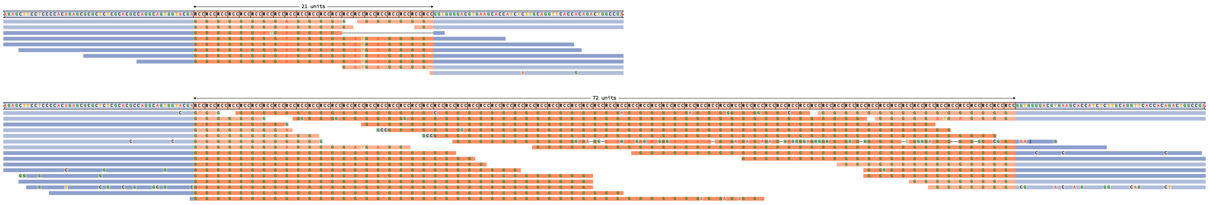

Figure S13: Family 2 IV:1

A canonical non-expanded allele, and an expanded allele.

Long-read data without presupposed AGT interruption (Figures S14-S15)

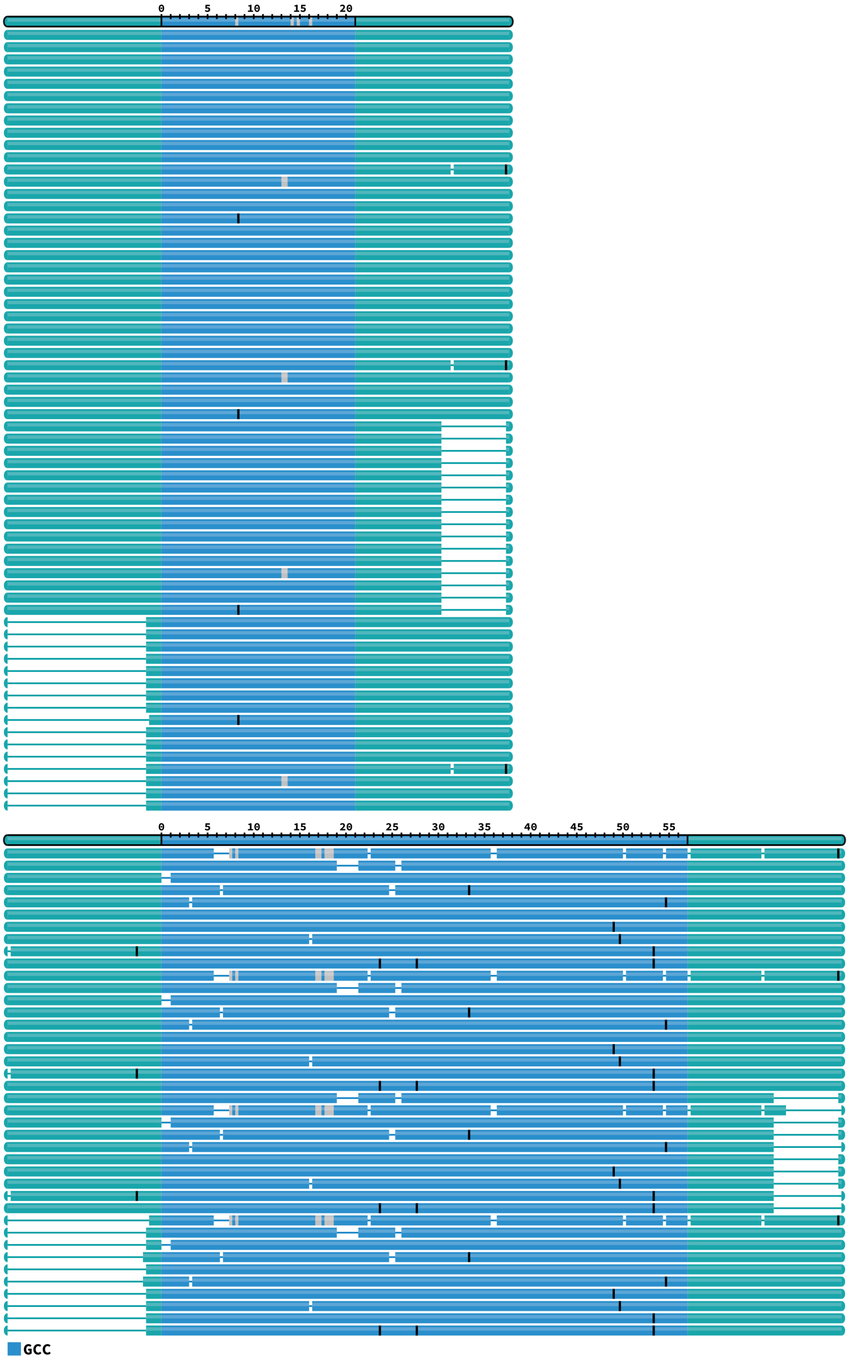

Figure S14: Family 1 III:1

This individual carried one canonical non-expanded allele (top) and one expanded allele (bottom). Black bars indicate insertions, horizontal lines indicate deletions. There are reads that perfectly span the repeat region. Estimated length is 57 trinucleotide units.

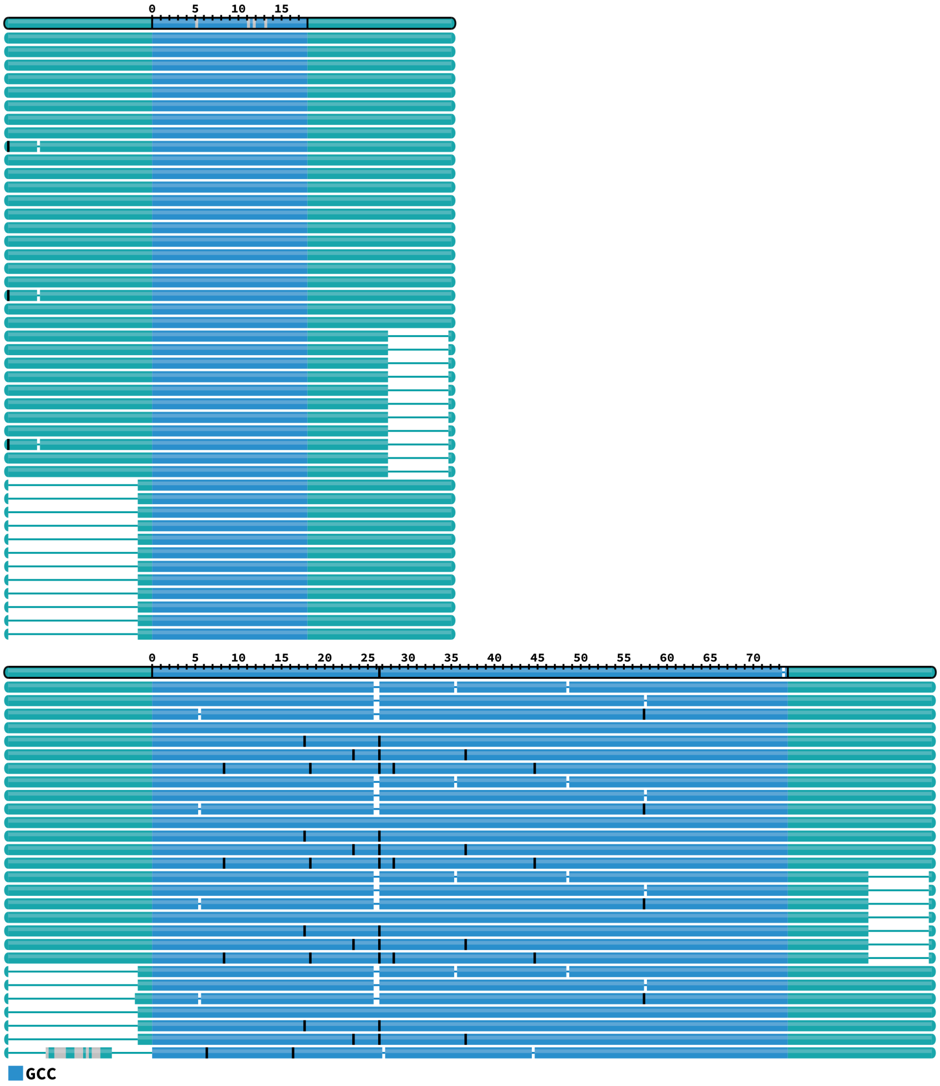

Figure S15: Family 1 IV:1
A non-expanded allele three units shorter than usual, and an expanded allele. Black bars indicate insertions, horizontal lines indicate deletions. There are reads that perfectly span the repeat region. Estimated length is 74 units.

Supplemental Tables

families	chr	start	end	length [kbp]	min-seed	min-extend	min-output	min-mac	min-markers
1	16	72,676,268	74,358,156	1,682	1	0.5	1	6	80
1, 2	16	72,676,268	73,070,083	394	0.5	0.3	0.5	6	60
1, 2, 3	16	72,676,268	73,070,083	394	0.3	0.15	0.3	6	45
1, 2, 3, 4	16	72,676,268	72,987,637	311	0.3	0.15	0.3	6	45
1, 2, 3, 4, 5	16	72,725,757	72,836,593	111	0.05	0.02	0.05	6	20

Table S1: Genomic segments shared by affected individuals in Families 1-5
Coordinates and lengths of the genomic segments shared by affected individuals from Families 1–5 as indicated in the first column, as identified by hap-ibd. The segment shared by all five families was 111 kpb in length (bottom row). Families 2, 4, and 5 are in this analysis represented by their respective probands. Family 1 is represented by individuals III:1, III:2, and IV:1, family 3 is represented by only individual III:1. The last five columns are input parameters to the hap-ibd program. Analyses from Family 1 and 2 were used prior to the identification of the *ZFHX3* repeat expansions, analyses from Families 3–5 were added later to better define an underlying shared haplotype. Reference genome hg19.

chr	start	end	motif	gene	region	p-value	Bonf. p-value
16	72,821,250	72,822,088	CCG	ZFHX3	exonic	0.07864	1.0

Table S2: *ZFHX3* as found by ExpansionHunter Denovo
P-values both before and after Bonferroni correction are given. The coordinates are to be considered approximate.

	Marker	Coordinates on chr 16
**Utah SCA4 family of Swedish origin[4]**		
Tight linkage reported	D16S397	66,738,337
Haplotype block with D16S397 in affected individuals	D16S398 – D16S421	66,129,139 – 67,295,807
**German SCA4 family[5]**		
Linked interval	D16S3019 – D16S512	66,129,331 – 74,067,749
**Two Swedish SCA4 families reported from Stockholm[6]**		
Linked interval	Same as above in German SCA4 family[5]	
**This report**		
Shared genomic segment* within in Family 1		72,676,268 – 74,358,156
Shared genomic segment* between families 1 and 2		72,676,268 – 73,070,083
Shared genomic segment* between families 1, 2, and 3		72,676,268 – 73,070,083
Shared genomic segment* between families 1, 2, 3, and 4		72,676,268 – 72,987,637
Shared genomic segment* between families 1, 2, 3, 4, and 5		72,725,757 – 72,836,593
***ZFHX3* GGY repeat**		
ZFHX3 NM_006885.4 exon 10 GGY repeat		72,821,593 – 72,821,656

Table S3: Comparison with previously reported SCA4 loci
The shared genomic segments were identified by hap-ibd. See Table S2 legend and main text for details. Reference numbers refer to the reference list in the main article.

hg19	hg38	ref	alt	rsID	GT	1KG p3	gnomADg	SweFreq
72,729,354	72,695,455	C	T	rs1041588471	0/1	n/a	0.00001546	n/a
72,768,356	72,734,457	A	G	rs183642752	0/1	0.001	0.001146	n/a
72,771,602	72,737,703	T	C	n/a	0/1	n/a	n/a	n/a

Table S4: Very rare SNVs within the IBD-ZFHX3 locus
These SNVs with very low frequencies were found in the genomic segment shared by all individuals with the repeat expansion, but not found in individuals without repeat expansion. The 1000 Genomes (1KG) frequencies are from the European population. The gnomAD genome frequencies are from the non-Finnish European population. SweFreq is based on the SweGen control data used in the manuscript.

Type of variation	Count	Frequency	Length	no. GGT	no. GAC	no. AGT	no. CGC
Normal	1935	84.13%	21	2	0	1	0
GGC > GAC	78	3.39%	21	2	1	1	0
+1 GGC 3'	64	2.78%	22	2	0	1	0
-3 GGC 3'	59	2.57%	18	2	0	1	0
+2 GGC 3'	36	1.57%	23	2	0	1	0
GGT > GGC 3'	24	1.04%	21	1	0	1	0
GGC > CGC 5'	22	0.96%	21	2	0	1	1
-1 GGT 5'	21	0.91%	20	1	0	1	0
-1 GGT, -1 GGC 3'	11	0.48%	19	1	0	1	0
-1 GGC 3'i	10	0.43%	20	2	0	1	0
-1 GGT, -2 GGC 3'	6	0.26%	18	1	0	1	0
-2 GGC 3'i, -1 GGT 5'	5	0.22%	18	1	0	1	0
7GGC,GGT,8GGC	5	0.22%	16	1	0	0	0
+1 GGT, +1 AGT 5'i	3	0.13%	23	2	0	2	0
+5 GGT 3'	3	0.13%	26	2	0	1	0
AGT > GGT	3	0.13%	21	3	0	0	0
-1 GGT, -6 GGC 3'	2	0.09%	14	1	0	1	0
-1 GGC 3'	2	0.09%	20	2	0	1	0
-5 GGC 5', -1 GGT 5', -1 AGT	2	0.09%	14	1	0	0	0
GGC > GGT 5'	1	0.04%	21	3	0	1	0
+2 GGC 3', GGC > CGC 5'	1	0.04%	23	2	0	1	1
+2 GGC 5'	1	0.04%	23	2	0	1	0
-1 GGC 5'	1	0.04%	20	2	0	1	0
GGC > GAC, -1 GGC 3'	1	0.04%	20	2	1	1	0
-2 GGC 3'	1	0.04%	19	2	0	1	0
6GGC,GGT,16GGC	1	0.04%	23	1	0	0	0
6GGC,GGT,15GGC	1	0.04%	22	1	0	0	0

Table S5: Variations in non-expanded alleles

The structure of 2,300 non-expanded alleles was analysed in detail. There were four types of deviations from the GGC repeat unit. These are, in order of prevalence, GGT, AGT, GAC, CGC. The CGC unit was found to always be located at the 5’ end of the repeat region, and can thus not be considered a true interruption. The GAC unit was found to always be located three units downstream of the AGT unit. The GGT units were observed in various locations, although never adjacent to each other, nor terminal (at any ends of the GGC repeats). In the vast majority of alleles, with two GGT units, the first unit is located two units upstream of the AGT unit, while the second unit is located six units downstream of the AGT unit (cf. Figure 2 for positions). The descriptions in the first column are either relative (to the most frequent normal allele as shown in Figure 2A), or absolute (row 13 and the two last rows). The latter type of description is used when the structure of the repeat deviates significantly from the normal. The 3’ and 5’ tags mean that the change from normal structure occurs at or near the downstream and upstream ends, respectively. An added “i” means the change occurred between the normal allele’s AGT and GGT units.

Expansion Hunter catalogs


```
[
    {
        "LocusId": "ZFHX3",
        "LocusStructure": "(RCC)*",
        "ReferenceRegion": "16:72821593-72821656",
        "VariantId": "ZFHX3",
        "VariantType": "Repeat"
    }
]
```


The *ZFHX3* STR in json format for use with ExpansionHunter, coordinates in hg19.


```
[
    {
        "LocusId": "ZFHX3",
        "LocusStructure": "(RCC)*",
        "ReferenceRegion": "16:72787694-72787757",
        "VariantId": "ZFHX3",
        "VariantType": "Repeat"
    }
]
```


The *ZFHX3* STR in json format for use with ExpansionHunter, coordinates in hg38.

Supplemental References

1. Wictorin, K., Brådvik, B., Nilsson, K., Soller, M., van Westen, D., Bynke, G., Bauer, P., Schols, L., and Puschmann, A. (2014). Autosomal dominant cerebellar ataxia with slow ocular saccades, neuropathy and orthostatism: a novel entity? Parkinsonism Relat Disord *20*, 748-754. 10.1016/j.parkreldis.2014.03.029.

2. Ramos, E.M., Carecchio, M., Lemos, R., Ferreira, J., Legati, A., Sears, R.L., Hsu, S.C., Panteghini, C., Magistrelli, L., Salsano, E., et al. (2018). Primary brain calcification: an international study reporting novel variants and associated phenotypes. Eur J Hum Genet *26*, 1462-1477. 10.1038/s41431-018-0185-4.

3. Batla, A., Tai, X.Y., Schottlaender, L., Erro, R., Balint, B., and Bhatia, K.P. (2017). Deconstructing Fahr's disease/syndrome of brain calcification in the era of new genes. Parkinsonism Relat Disord *37*, 1-10. 10.1016/j.parkreldis.2016.12.024.

4. Flanigan, K., Gardner, K., Alderson, K., Galster, B., Otterud, B., Leppert, M.F., Kaplan, C., and Ptacek, L.J. (1996). Autosomal dominant spinocerebellar ataxia with sensory axonal neuropathy (SCA4): clinical description and genetic localization to chromosome 16q22.1. Am J Hum Genet *59*, 392-399.

5. Hellenbroich, Y., Bubel, S., Pawlack, H., Opitz, S., Vieregge, P., Schwinger, E., and Zuhlke, C. (2003). Refinement of the spinocerebellar ataxia type 4 locus in a large German family and exclusion of CAG repeat expansions in this region. J Neurol *250*, 668-671. 10.1007/s00415-003-1052-x.

6. Engvall, M. (2020). Identification of disease genes in rare neurological conditions (Doctoral thesis. Dept. of Molecular Medicine and Surgery, Karolinska Institutet).
