# Supplementary material for: Exonic trinucleotide repeat expansions in ZFHX3 cause spinocerebellar ataxia type 4: A poly-glycine disease
Source: Am J Hum Genet. 2023 Nov 29;111(1):82–95. doi: 10.1016/j.ajhg.2023.11.008 (PMC10806739; doi:10.1016/j.ajhg.2023.11.008)
Supplement: Document S2. Article plus supplemental information [file mmc2.pdf]

Exonic trinucleotide repeat expansions in *ZFHX3* cause spinocerebellar ataxia type 4: A poly-glycine disease

Graphical abstract

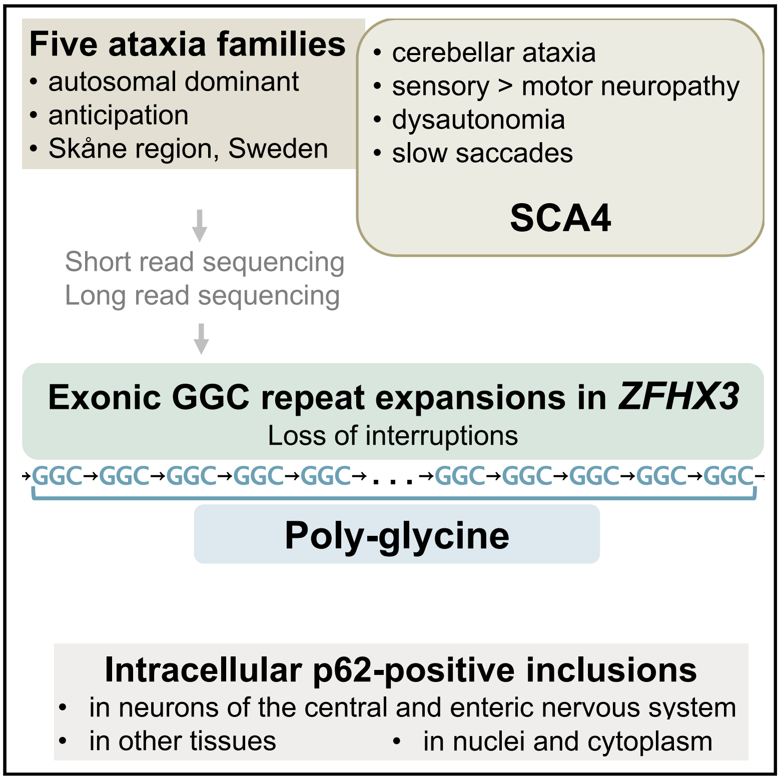

Authors

Joel Wallenius, Efthymia Kafantari, Emma Jhaveri, ..., Elisabet Englund, Hans Ehrencrona, Andreas Puschmann

Correspondence

andreas.puschmann@med.lu.se

In five families from Skåne region in Sweden, expanded repeat expansions in *ZFHX3* were identified as the cause for spinocerebellar ataxia type 4, SCA4. SCA4 is an autosomal-dominant disorder with progressive gait and balance disturbance and dyscoordination of movements. SCA4 might compromise nerves controlling automatic bodily functions.

Wallenius et al., 2024, The American Journal of Human Genetics *111*, 82–95
January 4, 2024 © 2023 The Authors.
https://doi.org/10.1016/j.ajhg.2023.11.008

ARTICLE

Exonic trinucleotide repeat expansions in *ZFHX3* cause spinocerebellar ataxia type 4: A poly-glycine disease

Joel Wallenius,[1] Efthymia Kafantari,[1] Emma Jhaveri,[1] Sorina Gorcenco,[1] Adam Ameur,[2] Christin Karremo,[1] Sigurd Dobloug,[1,3] Kristina Karrman,[4,5] Tom de Koning,[6] Andreea Ilinca,[1] Maria Landqvist Waldö,[7] Andreas Arvidsson,[1] Staffan Persson,[1] Elisabet Englund,[5,8] Hans Ehrencrona,[4,5] and Andreas Puschmann[1,9,*]

Summary

Autosomal-dominant ataxia with sensory and autonomic neuropathy is a highly specific combined phenotype that we described in two Swedish kindreds in 2014; its genetic cause had remained unknown. Here, we report the discovery of exonic GGC trinucleotide repeat expansions, encoding poly-glycine, in zinc finger homeobox 3 (*ZFHX3*) in these families. The expansions were identified in whole-genome datasets within genomic segments that all affected family members shared. Non-expanded alleles carried one or more interruptions within the repeat. We also found *ZFHX3* repeat expansions in three additional families, all from the region of Skåne in southern Sweden. Individuals with expanded repeats developed balance and gait disturbances at 15 to 60 years of age and had sensory neuropathy and slow saccades. Anticipation was observed in all families and correlated with different repeat lengths determined through long-read sequencing in two family members. The most severely affected individuals had marked autonomic dysfunction, with severe orthostatism as the most disabling clinical feature. Neuropathology revealed p62-positive intracytoplasmic and intranuclear inclusions in neurons of the central and enteric nervous system, as well as alpha-synuclein positivity. *ZFHX3* is located within the 16q22 locus, to which spinocerebellar ataxia type 4 (SCA4) repeatedly had been mapped; the clinical phenotype in our families corresponded well with the unique phenotype described in SCA4, and the original SCA4 kindred originated from Sweden. ZFHX3 has known functions in neuronal development and differentiation n both the central and peripheral nervous system. Our findings demonstrate that SCA4 is caused by repeat expansions in *ZFHX3*.

Introduction

An increasing number of genes associated with hereditary ataxias are being discovered, as a result of the increasing availability and constant improvements in next-generation sequencing (NGS) technology.[1,2] Hereditary ataxias form a broad spectrum of different entities, and cerebellar ataxia with neuropathy defines a certain subgroup within this spectrum.[3] In 2014, we described two kindreds with autosomal-dominant cerebellar ataxia and sensory and autonomic neuropathy.[4] The affected family members also had a characteristic slowness of horizontal saccades, and their ancestors could genealogically be traced to the same village in southern Sweden. We now report the discovery of exonic trinucleotide repeats in *ZFHX3* (MIM: 104155) and find that these repeats co-segregate with the disease phenotype in these two families. We have expanded the previously reported family's pedigree, provide clinical follow-up data 8–9 years after our first description, and report on the neuropathology of one affected individual who died at the age of 28 years. We identified four additional persons with ataxia from three independent Swedish families with the same disease phenotype and *ZFHX3* repeat expansions from our ataxia series, but the repeat expansions were absent from large in-house and national datasets from individuals with other diseases and from population controls. The gene's location within the previously described locus for spinocerebellar ataxia type 4 (SCA4 [MIM: 600223]) and the clinical overlap with SCA4 suggest that *ZFHX3* repeat expansions cause SCA4. This disorder might be a relatively common cause of autosomal-dominant ataxias, at least in the southern Swedish population.

Subjects and methods

Genealogical data and clinical examination

Family 1 and family 2 were described in 2014 by some of the authors,[4] and the authors had since remained in contact with the families and continuously cared for some of their affected members at their clinics. Family 2 had been described in 1978.[5] Additional affected members were identified via the families and/or from among persons with ataxia in the authors' clinics. Updated pedigree drawings are shown in Figure 1. All surviving affected family members

[1]Neurology, Department of Clinical Sciences Lund, Lund University, Skåne University Hospital, 222 42 Lund, Sweden; [2]Department of Immunology, Genetics and Pathology, Science for Life Laboratory, Uppsala University, 751 23 Uppsala, Sweden; [3]Department of Neurology, Helsingborg General Hospital, 252 23 Helsingborg, Sweden; [4]Division of Clinical Genetics, Department of Laboratory Medicine, Lund University, 222 42 Lund, Sweden; [5]Department of Clinical Genetics, Pathology and Molecular Diagnostics, Office for Medical Services, Region Skåne, 221 85 Lund, Sweden; [6]Pediatrics, Department of Clinical Sciences Lund, Lund University, 221 84 Lund, Sweden; [7]Division of Clinical Sciences Helsingborg, Department of Clinical Sciences Lund, Lund University, 221 84 Lund, Sweden; [8]Pathology, Department of Clinical Sciences Lund, Lund University, Skåne University Hospital, 222 42 Lund, Sweden; [9]SciLifeLab National Research Infrastructure, Lund University, 221 84 Lund, Sweden
*Correspondence: andreas.puschmann@med.lu.se
https://doi.org/10.1016/j.ajhg.2023.11.008.
© 2023 The Authors. This is an open access article under the CC BY license (http://creativecommons.org/licenses/by/4.0/).

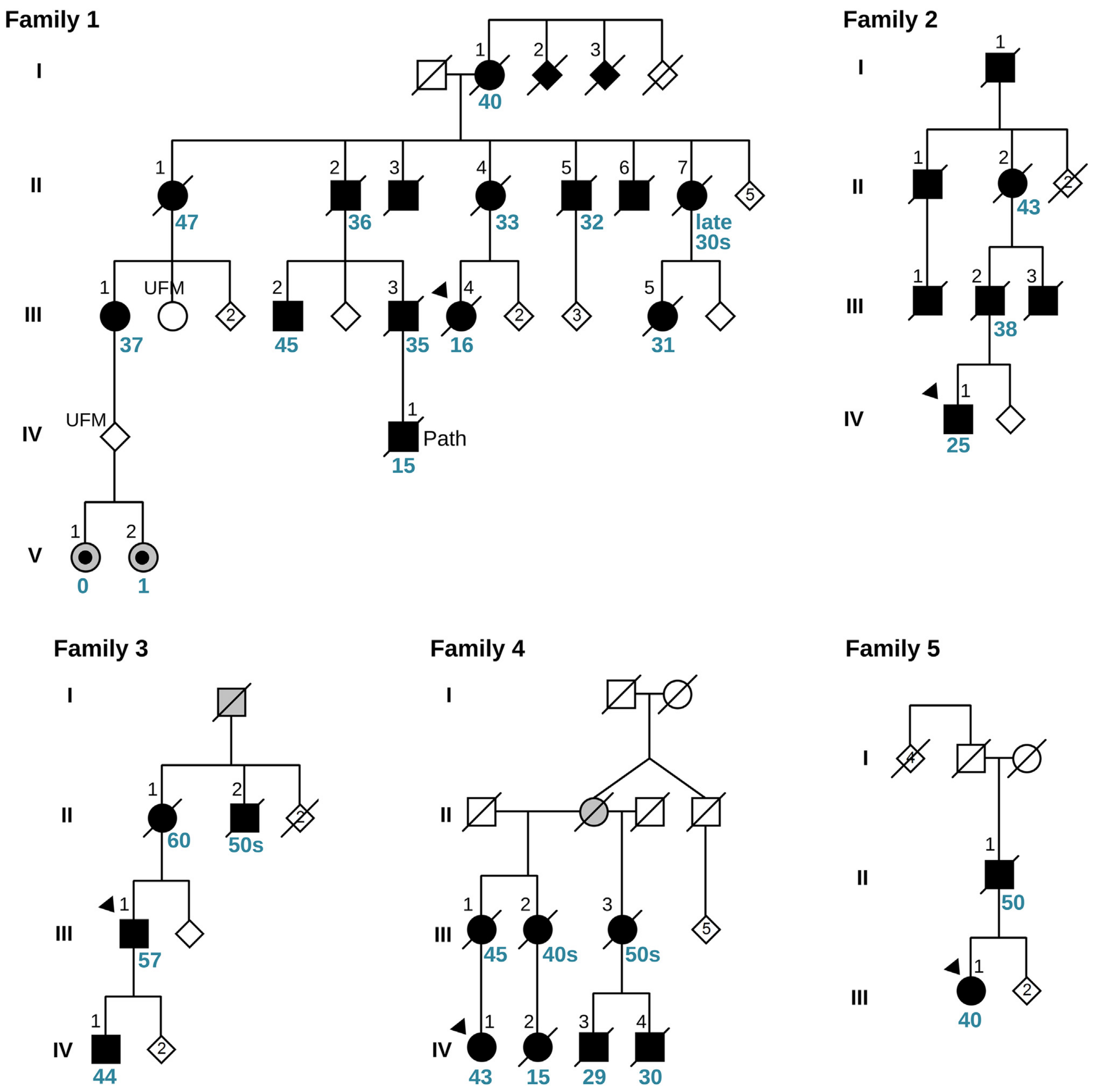

Figure 1. Family pedigrees
Standard symbols were used. Black symbols represent individuals with cerebellar ataxia with sensory and autonomic neuropathy. Probands are indicated with black triangles. Gray symbols show family members who had gait and balance problems according to family history. Gray symbols with a central black dot represent two siblings who were evaluated within this study but were considered to have a neurodevelopmental disorder that is different from cerebellar ataxia with sensory and autonomic neuropathy, and who do not have *ZFHX3* repeat expansions. The black numbers above the symbols are individual identifiers. For those family members for whom age at symptom onset was available, it is shown below the symbols in blue. Sex and gender of non-affected individuals was disguised, and sibling order partly altered to protect confidentiality. For the same reason, a few unaffected family members are not shown. UFM, unaffected family member who was analyzed genetically within this study.

were re-contacted in 2022, and all were interviewed and re-examined in a standardized manner in the context of the present study. As for the examinations for our 2014 publication, the international cooperative ataxia rating scale (ICARS) was used for examining and comparing specific ataxia symptoms[6]; affected members from the newly identified SCA4-affected families had been examined according to the scale for the assessment and rating of ataxia (SARA).[7] After the identification of three additional families carrying *ZFHX3* repeat expansions from our ongoing ataxia research study,[8] their members were also re-approached for this study. Medical records of deceased and alive affected members were reviewed. Our study was approved by the Regional Ethics Review Board in Lund and the Swedish Ethical Review Authority. All adult participants provided informed consent. Two children were examined by a pediatrician in the context of their clinical evaluation; written informed consent was provided by their parents.

Genetic analysis

Blood was drawn from affected and unaffected individuals. Short-read whole-genome sequencing was performed at the Department of Clinical Genetics, Region Skåne, the Center for Translational Genomics, Lund University or at Centogene, Rostock, Germany. Clinical reports from whole-genome examinations filtered for ataxia and neuropathy genes that include known clinically relevant repeat expansions were obtained but failed to elucidate the cause of the disease. We continued to analyze WGS datasets from families 1 and 2 to search for variants in genes not previously related to neurological diseases or ataxias. We explored different approaches depending on whom we assumed to be affected by the same disease; we analyzed only family 1 or both families together, and we either included or excluded family 1's two young children (V:1 and V:2), who had a different clinical phenotype that included infantile onset and cognitive and behavioral symptoms, albeit with ataxia. Family 1 III:UFM and other in-house genomes from unrelated individuals without ataxia were used as references where needed.

Genomic segments shared by affected but not by unaffected family members were detected by identity-by-descent (IBD) analysis. We predicted IBD segments by first phasing variant calling format (VCF) files from families 1 and 2 with Beagle v5.4[9] and using the 1000 Genomes reference panel, then running hap-ibd.[10] Non-default settings for hap-ibd are provided in Table S1, and a full description of each setting is available in the hap-ibd documentation (https://github.com/browning-lab/hap-ibd).

Single-nucleotide variants (SNVs) were either supplied in VCF files by the sequencing cores or produced from supplied binary alignment map (BAM) files via the Genome Analysis Toolkit v.4.2.6.1.[11] VCF files were annotated and filtered with VEP v.109[12] so that variants with minor-allele frequencies above 0.01 were excluded. Copy-number variants (CNVs) were called with the Genome Analysis ToolKitv.4.2.6.1 according to the Broad Institute's CNV calling tutorial (https://gatk.broadinstitute.org/hc/en-us/articles/360035531152), with default settings. The input-intervals file spanned the entire GRCh37 (hg19) genome in 1,000 bp intervals. SNV and CNV calls were cross-correlated with the genomic segments shared by affected members and curated manually with the Integrative Genomics Viewer v.2.12.2 for CNVs.[13] CNVs that fit the co-segregation patterns were manually screened with DECIPHER[14] so that common CNVs would be excluded.

Known short tandem repeat (STR) expansion loci were examined with ExpansionHunter v.5.0.0.[15] The bundled variant catalog was extended to include the more recently described *FGF14*[2,16] and *NOTCH2NLC*[17] pathogenic repeats that cause similar clinical or pathological phenotypes. No expansions were found in either of these loci. STR expansion loci not previously associated with disease were assessed with ExpansionHunter Denovo v.0.9.0 from 2020,[18] which is still the most recent version. Results were cross-correlated with the shared genomic segments and manually curated. From this emerged a trinucleotide repeat expansion in *ZFHX3* as a single finding. This expansion was confirmed by its addition to the ExpansionHunter variant catalog, the re-running of ExpansionHunter, and finally visual examination of the locus with graphics produced by REViewer v.0.2.7.[19]

Screening of larger datasets

After the identification of expanded *ZFHX3* repeats associated with the disease phenotype in families 1 and 2, we screened additional available datasets for this repeat expansion. We queried 25 WES and 64 WGS datasets from 89 persons with ataxia of unresolved cause from our ataxia series, including those previously published.[8] We also analyzed *ZFHX3* repeats in 90 WES and 60 WGS datasets from individuals with other neurological diagnoses in our research database and queried the SweGen dataset with Illumina short-read WGS data from 1,000 unrelated Swedish individuals, most of whom (942) were probands selected from more than 85,000 twins in the Swedish Twin Registry, as previously described.[20] We analyzed the architecture of normal repeats in our 90 WES and 60 WGS in-house datasets (300 alleles) and in all 2,000 alleles from SweGen with ExpansionHunter and REViewer.

Analysis of shared haplotype of the five families

We performed three additional IBD predictions, for which we added WGS data from members of families 3, 4, and 5, to confirm a shared haplotype and to investigate a possible relation between all five families. We searched for SNVs with the lowest frequency in population databases (gnomAD NFE and SweGen) within the IBD genomic area that included *ZFHX3* and compared their occurrence among affected and unaffected members of all five families.

Long-read sequencing

Long-read genome sequencing was performed on DNA from individuals III:1 and IV:1 from family 1. These individuals were selected because they displayed a large difference in disease severity and age at onset (37 and 15 years). The samples were run on the PacBio Revio instrument, generating 77.2 Gb and 56.5 Gb of high-quality (>QV20) long-read data for individuals III:1 and IV:1, respectively. PacBio long-read data from 27 individuals from the Human PanGenome Consortium[21] were used for comparison. We analyzed the data manually to determine the exact number and architecture of repeats in *ZFHX3* and to exclude structural changes within the 16q22 SCA4 locus.

Neuropathology

Individual IV:1 from family 1 died unexpectedly at the age of 28 years, and a clinical postmortem examination was performed to elucidate the cause of his death. An extensive neuropathological examination of the central and peripheral nervous system was performed according to established procedures at the Department of Pathology, Region Skåne. It included conventional and immunohistochemical stains of brain regions and of autonomous nerves in the skin, gastrointestinal plexus, and epicardium. Antibodies against hyperphosphorylated tau, alpha-synuclein, and TDP-43 were used as described previously.[22] For evaluation of p62 protein pathology, anti-nucleoporin/p62 LCK ligand manufactured by BD Transduction Laboratories (clone 3, catalog no. 610832) was used in 1:100 dilution.

Results

Genetic analysis of families 1 and 2

No deleterious single-nucleotide variant, indel, copy-number variant, or previously described short tandem repeat co-segregating with the disease was identified in families 1 or 2. We did not identify a common genetic cause when including the two individuals with a severe neurodevelopmental phenotype (family 1, V:1 and V:2) and excluded these from subsequent analyses. Our IBD analyses revealed 199 genomic segments shared by all analyzed affected individuals in family 1, except V:1 and V:2. These segments ranged in length

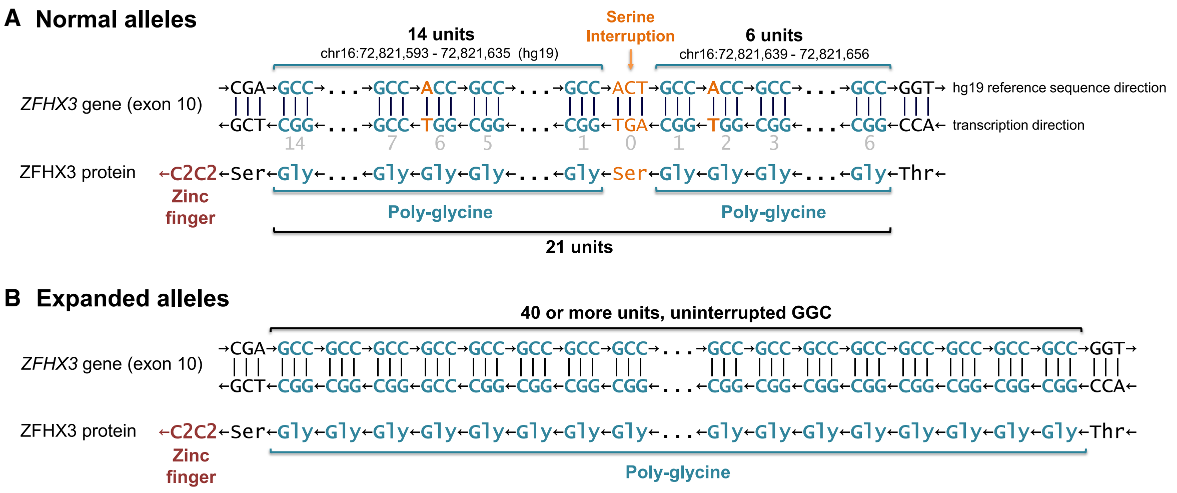

Figure 2. ***ZFHX3*** **locus harboring the repeat expansion encoding poly-glycine**
ZFHX3 is encoded on the negative strand in the hg19 reference genome. For clarity, the negative strand sequence is included in the figure. Unless otherwise specified, we discuss the repeat region from the perspective of the negative strand.
(A) In the vast majority of non-expanded alleles, the repeat region consists of exactly 18 GGC units, two GGT units, and a single AGT interruption; we report the total repeat length as 21 for these alleles. The locations of the GGT units (which also code for glycine but interrupt the repetitive GGC pattern on the DNA level) are given relative to the AGT interruption (light gray numbers). Such a normal allele results in a protein with 20 glycine residues interrupted by a single serine at position 7. This poly-glycine is followed by a single serine residue and a C2C2-type zinc finger motif. Generally, zinc finger motifs are considered elements that can bind to DNA in a sequence-specific manner. See main text for the exact composition of this locus in the minority of non-expanded alleles.
(B) There were no detectable interruptions in any of the expanded alleles from the affected members of the five families described here—the repeat region was entirely composed of GGC units.

from 1 kbp to 2.7 Mbp, with a median of 108 kbp; one of the longest included a 1.6 Mbp segment on chromosome 16. When the proband of family 2 (IV:1) was included, the shared segment on chromosome 16 was narrowed down to 394 kbp (Table S1). Within this genomic segment, a GGC (poly-glycine) repeat expansion in the final exon of *ZFHX3* (MANE Select transcript, GenBank: NM_006885.4) co-segregated perfectly with disease status in both families (Figure 2; Tables 1 and S2). The two children (family 1, V:1 and 2) and their unaffected parent had normal *ZFHX3* repeat lengths, as did the unaffected family members in family 1 (III:UFM and IV:UFM). Long-read sequencing revealed the exact length of the repeat expansion to be 57 uninterrupted GCC repeats in family 1 III:1 (age of onset 37 years) and 74 uninterrupted GCC repeats in IV:1 (age of onset 15 years).

Screening of larger ataxia datasets and identification of additional families

Re-analyzing WES and WGS data from our ataxia series identified four additional individuals with ataxia from three additional families with expanded *ZFHX3* repeats, the probands of families 3–5, and individual family 3 IV:1 (Tables 1 and 2; Figure 1). When including the additional families, the genomic segment shared by all affected individuals shortened to 111 kbp; within this segment three very rare intronic SNVs were found exclusively in repeat expansion carriers, further defining a shared haplotype (Table S4). We did not find expanded alleles among persons with ataxia or controls in our in-house research database with individuals with other neurological diagnoses from southern Sweden, nor among 2,000 alleles in the SweGen database.

Normal repeat length and locus architecture

We used 300 non-expanded alleles from our in-house WES and WGS datasets, as well as 2,000 non-expanded alleles from the SweGen WGS dataset, to determine the normal *ZFHX3* repeat structure. All non-expanded alleles had interruptions within the GGC repeat; these interruptions were predominantly synonymous GGT and a non-synonymous AGT encoding serine (Figure 2; Table S5). The vast majority of alleles had the exact structure depicted in Figure 2. The remaining alleles deviated in multiple ways, as detailed in Table S5. The distribution of total repeat lengths is presented in Figure 3, both for the control datasets mentioned above as well as for 27 additional controls with long-read sequencing. In expanded alleles, there were no visible interruptions of any kind in short-read WGS data, but short reads might not suffice to entirely exclude interruptions because the repeat region is longer than the short read length of 150 bp. Long-read sequencing of two affected individuals confirmed a complete lack of interruptions: GGC was the only repeat unit.

Genealogical data and clinical examination

We have collected clinical information on forty members affected by neurological disease in the five families (Figure 1). All five families originate from Skåne (approximately 1.4 million inhabitants), the southernmost region of Sweden, where our center is located. Fifteen affected members were

examined by the authors of the previous[4] and/or the present study. Tables 1 and 2 summarize the family members' clinical phenotypes. Clinical descriptions are provided below and as a Supplemental Note. We also refer to our previous work[4] for additional detailed clinical descriptions of families 1 and 2; these descriptions include videos of several affected family members.

Our genetic studies showed that family 1's individuals V:1 and V2 did not share the repeat expansion that we found in all other genetically examined affected individuals from families 1 and 2. These two siblings, V:1 and V2, had signs and symptoms (see Supplemental Note) of a more severe neurodevelopmental disorder of hitherto undetermined genetic cause.

The remaining 38 affected members of families 1 to 5 shared clinical features; all had autosomal-dominant cerebellar ataxia with sensory and autonomic neuropathy. They developed gait or balance disturbance at a mean age at disease onset of 37.6 years, but with a range from 60 to 15 years in a pattern compatible with anticipation (Tables 1 and 2; Figure 1). Electroneurography showed signs of sensory or sensory-motor neuropathy; frequently, the sensory neuropathy signs were pronounced but motor findings relatively moderate. In all but two instances in our families, the affected child of an affected individual manifested disease symptoms earlier than the parent (Figure 1). The initial symptom was balance disturbance, commonly perceived as a tendency to stumble while walking. Symptoms progressed slowly but relentlessly, and all affected individuals had both gait ataxia and limb ataxia. Most of the examined adult family members with ataxia had lost the ability to write their name legibly with a pen or to stand without support. Slow horizontal saccades were seen in all individuals with *ZFHX3* repeat expansions, there was no nystagmus, and smooth pursuit eye movements were frequently unimpaired. When directing their gaze sideways, some of the affected individuals involuntarily turned their head in this direction and/or showed simultaneous involuntary perioral muscle activation. Mirror movements had been noted in one individual.[4] Individuals who were younger at the onset of ataxia developed more severe additional symptomatology. Dysautonomia was a common sign. It mostly manifested with symptomatic orthostatic hypotension, which became very severe in members of the younger generations, and as difficulties with bowel or bladder control. Seven affected individuals with earlier onset had involuntary weight loss and/or muscle wasting accompanied by documented underweight body mass index (Tables 1 and 2) that in three of them became severe and was considered to have contributed to their deaths at ages 28–47 years. These individuals had not received feeding tubes and had dysphagia, and two of them died in the hospital after cardiac arrest. Other affected individuals had less severe swallowing problems and were able to maintain a higher BMI. Family members with severe symptomatic orthostatism received blood-pressure-elevating medication (fludrocortisone, etilefrine, droxidopa, midodrine) in combinations and dosages that changed over the years; one person was treated with erythropoietin with the aim of increasing blood volume and pressure; the effect of these measures varied. Individuals with diarrhea reported a benefit from loperamide tablets.

Postmortem examination and neuropathology

Individual IV:1 from family 1 had been treated with cefotaxime for urinary-tract infection for 11 days until 5 days prior to his death. He developed peritonitis after surgical PEG insertion, had a cardiac arrest, and was resuscitated but died the following day. Postmortem examination showed bilateral pneumonia with pulmonary edema and acute peritonitis. There was widespread invasive mycosis in most examined tissues, including the myocardium, lungs, gastrointestinal walls, skin, skeletal muscles, and brain. *Candida dublinensis* was identified in blood cultures after his death. Microscopic examination revealed intracellular inclusions in hepatocytes. The cerebrum exhibited mild atrophy, and there was mild cerebellar atrophy (see supplemental note). The substantia nigra appeared pale. Microscopic examination showed widespread foci of selective eosinophilic neuronal death attributed to the cardiac arrest and subsequent resuscitation.[22] Signs of chronic disease included neuronal loss and gliosis in the cerebellum, brain stem, and spinal cord. A marked loss of pigmented cells was seen in the substantia nigra, and a moderate cell loss was seen in the locus ceruleus. The number of Purkinje cells was markedly reduced in the cerebellar hemispheres and moderately reduced in the vermis, and there was Bergmann gliosis. The dentate nucleus showed gliosis and a reduced number of cells; the remaining cells were atrophic and had perinuclear halos. There were abundant intranuclear and less abundant intracytoplasmic inclusions in brainstem neurons (Figures 4A–4C) and singular inclusions of the same types in the cerebral cortex. The inclusions were sharply delineated, and their aspect and distribution differed from the pathology of viral infections (CMV, HSV, rabies) that are known to be associated with intracellular inclusions; immunostaining against HSV was negative. There were no inclusions in cerebellar sections. Peripheral nerve fibers in the lower extremities showed marked thinning but with relatively preserved myelin, as in axonal death. Epicardial nerve fibers appeared normal in appearance. Skeletal muscle sections had normal appearance. Nerve cells of the myenteric plexus in the esophagus contained inclusions (Figures 4E and 4F). The inclusions stained for p62 (Figures 4D–4F). Alpha-synuclein immunoreactivity was seen in brainstem and medulla oblongata neurons (Figures 4G and 4H), in the hippocampus, and in gastrointestinal-tract myenteric ganglion cells, which also were atrophic. Mild alpha-synuclein immunoreactivity was seen in thin intradermal nerve fibers of the abdominal wall. Alpha synuclein immunopositivity was seen in neurites and in fine granular depositions within the cytoplasm of neurons, in different patterns than the p62-positive inclusions. There were no Lewy bodies.

Table 1. Clinical phenotype and genetic results of affected members of family 1

	Family 1											
Individual	II:1	II:2	II:4	II:5	III:1	III:2	III:3	III:4	III:5	IV:1	V:1	V:2
Age at onset (years)	47	36	33	32	37	45	35	16	31	15	0.5	1.5
Age at most recent evaluation or age at death	78	70	75	60	66	57	47	42	44	28	8	4
Initial symptom(s)	BD, numbness in feet	BD, intention tremor	BD	BD	BD	BD	gait and limb ataxia	BD	BD	BD	delayed psycho-motor development	abnormal gait, limb ataxia
Walking aid (most recent documented)	wheelchair	wheelchair	wheelchair	wheelchair	wheelchair	wheelchair	wheelchair	wheelchair	wheelchair	walker at home, wheelchair outside	walker	none
Dysarthria	++	++	yes	+++	+++	+	+++	+++	+++	yes	+	+
Dysphagia	NA	NA	yes	yes	++	no	+++	+	+	+++	+	no
Slow ocular saccades	NA	NA	yes	NA	yes	yes	NA	yes	yes	yes	no	no
Paresthesia	NA	NA	no	NA	no	tingling sensation in feet	no	NA	burning sensation in hands and feet	NA	Pain in legs	tingling sensation in feet and pain in legs
Sensory impairment in extremities	yes	yes	yes	yes	yes	yes	NA	yes	yes	yes	no	no
Deep tendon reflexes	areflexia	areflexia	areflexia	NA	areflexia	areflexia	hyporeflexia	hyporeflexia	areflexia	areflexia	normal	hyporeflexia
Other symptoms/diagnoses	head tremor; dysphagia	transient fasciculations	painful cramps in left thigh	pronounced muscle wasting, p araparesis, reduced muscle strength and increased tone in arms	involuntary leg jerks and facial twitching	fasciculations	torticollis, hallucinations, absence-like episodes	mirror movements in hands[4]	inverted foot posture, i nvoluntary facial twitching	profuse s weating, cough, excessive airway mucus production, involuntary facial twitching, atypical autism	hypotonic infant, hyperlaxity, myoclonic jerks, behavioral problems, everted foot posture	hyperlaxity, myoclonic jerks, attention deficit
Most recent ICARS score, max 100 (age)	NA	NA	43 (67)	NA	62.2 (66)	56.5 (57)	NA	37 (38)	57.5 (43)	21 (20)	NA	NA

(*Continued on next page*)

Table 1. *Continued*

	Family 1											
Individual	II:1	II:2	II:4	II:5	III:1	III:2	III:3	III:4	III:5	IV:1	V:1	V:2
Cerebellar atrophy (imaging modality, age)	+++ (CT, 76)	++ (CT, 72)	NA	+++ (MR, 60)	+++ (MR, 57)	++ (MR, 43)	++ (MR, 39)	+++ (MR, 32)	+ (MR, 33)	0 (MR, 20)	+++ (MR, 2)	++ (MR, 2)
Electroneurography	NA	NA	SN	NA	SN	S-MN	NA	SN	S-MN	S-MN	normal	NA
Orthostatic hypotension	NA	NA	NA	NA	fluctuating blood pressure	no	yes	yes	yes	yes	no	no
Bowel symptoms	no	no	NA	NA	constipation	bowel urgency	no	postprandial diarrhea	constipation	constipation	incontinence and constipation	constipation
Urinary symptoms, sexual dysfunction	urge incontinence		NA	erectile dysfunction	recurring UTI's	no	UR	no	UR	UR	incontinence	incontinence
Unintended weight loss and BMI <18	NA	NA	NA	yes	yes	no	yes	yes	yes	yes	no	no
DNA-analysis	NA	NA	NA	NA	WGS	WGS	NA	NA	NA	WGS	WGS	WGS
Nr of repeat units Short-read, allele 1	NA	NA	NA	NA	52	42	NA	NA	NA	56	21	21
Nr of repeat units Short-read, allele 2	NA	NA	NA	NA	21	21	NA	NA	NA	18	21	21
Nr of repeat units Long-read, allele 1	NA	NA	NA	NA	57	NA	NA	NA	NA	74	–	–
Result: *ZFHX3* repeats	NA	NA	NA	NA	expanded	expanded	NA	NA	NA	expanded	normal	normal

More detailed clinical descriptions and information on additional family members are found in the supplemental note. Repeat lengths are provided as outlined in Figure 2. In addition, the following members of family 1 were analyzed genetically but are not shown in the Table: III:UFM carried 21 and 21 repeats. IV:UFM carried 18 and 21 repeats. The second unaffected (married-in) parent of V:I and V:II had 21 and 22 repeats. BD = balance disturbance; BMI = body mass index; CT = computed tomography; FBGC = familial basal ganglia calcification (see supplemental note); ICARS = international cooperative ataxia rating scale; ID = intellectual dysfunction; MRI = magnetic resonance imaging; NA = not assessed; SARA = scale for the assessment and rating of ataxia; SN = sensory neuropathy; S-MN = sensorimotor neuropathy; UR = urinary retention; UTI = urinary-tract infection; WES = whole exome sequencing; WGS = whole genome sequencing; '+' = mild; '++' = moderate; '+++' = severe. Portions of the information in Tables 1 and 2 are reprinted, in modified and updated form, from [4] with permission from Elsevier.

Table 2. Clinical phenotype and genetic results of affected members from families 2–5

	Family 2			Family 3		Family 4		Family 5	
Individual	II:2	III:2	IV:1	III:1	IV:1	III:1	IV:1	II:1	III:1
Age of onset (years)	43	38	25	57	44	45	43	50	40
Age at most recent evaluation/age at death	75	53	46	80	50	52	51	79	50
Initial symptom(s)	BD	BD, worsened fine motor skills	BD	BD	BD	BD	Gait and limb ataxia	BD	BD
Walking aid (most recent documented)	requires support	wheelchair	wheelchair	wheelchair	none	wheelchair	requires support	wheelchair	walking sticks
Dysarthria	yes	yes	+++	yes	yes	yes	++	yes	no
Dysphagia	no	no	no	yes	no	yes	++	yes	yes
Slow ocular saccades	NA	pathological saccades	yes	yes	yes	NA	yes	NA	NA
Paresthesia	no	no	NA	no	no	NA	no	no	yes
Sensory impairment in extremities	yes	yes	yes	yes	no	NA	yes	yes	yes
Deep tendon reflexes	areflexia	areflexia	areflexia	areflexia	areflexia	NA	hyporeflexia	areflexia	areflexia
Other symptoms/ diagnoses	upgaze palsy, lower-back pain	increased muscle tone in legs at night	involuntary facial twitching, FBGC	restless legs	neuralgia, subjective cranial sensation	painful leg cramps	anxiety, painful leg cramps	–	flushes, restless legs
Most recent ICARS score, max 100 (age)	NA	NA	54,5 (46)	NA	23 (50)	NA	30 (50)	NA	NA
Most recent SARA score (age)	NA	NA	NA	NA	12 (50)	NA	NA	NA	NA

(*Continued on next page*)

Table 2. *Continued*

	Family 2			Family 3		Family 4		Family 5	
Individual	II:2	III:2	IV:1	III:1	IV:1	III:1	IV:1	II:1	III:1
Cerebellar atrophy (imaging modality, age)	NA	++ (CT, 44)	++[a] (MR, 37)	NA	++ (MRI, 49)	NA	++ (MRI, 43)	++ (CT, 51)	NA
Electroneurography	S N	NA	NA	S-MN	NA	NA	NA	S-M N	S N
Orthostatic hypotension	NA	no	yes	NA	no	NA	yes	yes	yes
Bowel symptoms	NA	bowel urgency and diarrhea	bowel urgency and diarrhea	constipation	no	NA	alternating constipation and diarrhea	–	constipation
Urinary symptoms	NA	UR and enuresis nocturna	no	yes	no	NA	urgency	UR	urgency
Unintended weight loss and BMI <18	NA	NA	yes	no	no	NA	yes	–	no
DNA-analysis	NA	NA	WGS	WGS	WGS	NA	WGS	NA	WGS
No. of repeat units Short-read sequencing allele 1	NA	NA	72	46	56	NA	56	NA	59
No. of repeat units Short-read sequencing allele 2	NA	NA	21	21	18	NA	21	NA	21
Result: *ZFHX3* repeats	NA	NA	expanded	expanded	expanded	NA	expanded	NA	expanded

[a]= CT showed calcifications in internal and external globus pallidus and in subcortical white matter; Please see also the legend to Table 1.

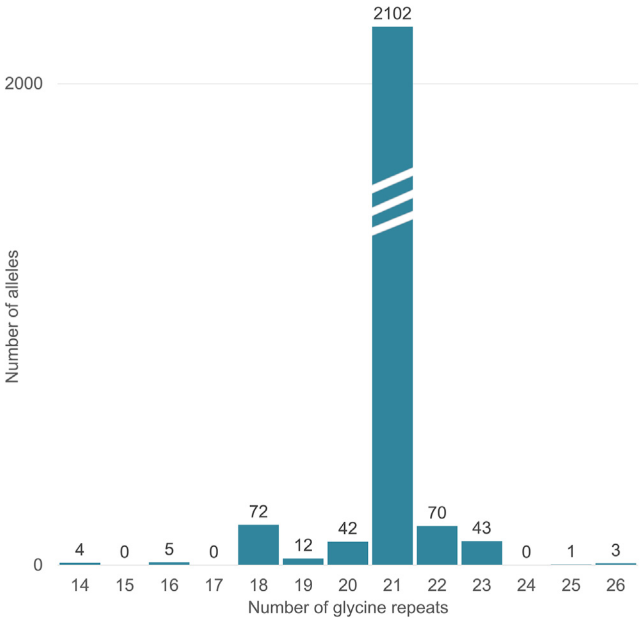

Figure 3. *ZFHX3* repeat lengths in non-expanded alleles
Distribution of the total number of STR repeat units, including potential interruptions, as determined from the DNA sequence of 1,000 unaffected individuals from the Swedish SweGen WGS database,[20] 150 in-house WES or WGS datasets without the *ZFHX3* repeat expansion, and 27 PacBio datasets from the Human PanGenome Reference Consortium.[21] Numbers above the bars indicate the number of alleles. By contrast, expanded alleles in affected individuals (not shown above) were 42–72 GGC repeats in length, as determined from short-read WGS data, and 57 or 74 GGC repeats as determined by long-read sequencing of two affected individuals (see Table 1 and text), offering clear delineation between non-expanded and expanded alleles.

Discussion

We report the association of exonic GGC trinucleotide repeat expansions, encoding poly-glycine, in *ZFHX3* with autosomal-dominant cerebellar ataxia with sensory and autonomic neuropathy in five Swedish families, including two whose clinical phenotype we previously described in detail.[4] Affected individuals developed gait and balance disturbances at variable ages, and the families displayed clear evidence for anticipation, which is compatible with a repeat expansion as its genetic cause. Key clinical symptoms include ataxia, neuropathy with predominantly sensory findings, slow saccades, and cerebellar atrophy on MRI and neuropathology. Orthostatic hypotension, loss of bladder or bowel control, or other signs and symptoms of dysautonomia occurred and severely impaired some of the affected individuals, especially those with earlier onset and more severe disease.

In data from large national and in-house cohorts, we have been able to define the normal length of the identified repeat to 14–26 trinucleotides encoding poly-glycine; the most common length by far was 21 repeat units, including interruptions. In short-read data, expanded repeats were estimated to be between 42 and 72 units of pure GGC. Short-read data are less accurate when a repeat expansion approaches the read length (approximately 150 base pairs or 50 trinucleotide repeat units), but non-expanded alleles could clearly be delineated in short-read data. A clear separation between the distribution of normal alleles (14–26 repeats) and pathogenic expansions (42–74 repeats) was seen. Two individuals were analyzed by both short-read and long-read sequencing; their repeat length was estimated to be 52 and 56 by short-read technology, but long-read sequencing revealed 57 and 74, respectively; this suggests that estimations based on short-read data do not accurately represent the true lengths of the expanded repeats in these two individuals. Although the anticipation observed in our families correlates well with the lengths observed in the long-read data, further research is needed to firmly establish a correlation between *ZFHX3* repeat length and age of onset or the severity of clinical symptoms. Long-read sequencing confirmed pure GCC repeats in these two expanded alleles. By contrast, all 2,300 non-expanded alleles that we assessed had one or more interruptions. Repeat interruptions are known to have a stabilizing effect on repeat length.[23] We hypothesize that the interruptions stabilize the repeat length in unaffected individuals and that disappearance of interruptions might have been the first step toward gradual expansion of the repeat from generation to generation.[24]

All five families originated from the limited geographic area of Skåne, the southernmost region of Sweden, but the family trees could not be linked on the basis of existing genealogical information. Genetic data revealed that in all examined affected members of the five families, the *ZFHX3* repeat expansion was located within a shared genomic segment of at least 111 kbp in length, indicating a common founder event many generations ago. The oldest members of family 4 and 5 were not known to have neurological symptoms. This is compatible with the possibility that the actual founder event was spontaneous mutations that eliminated or reduced the number of repeat interruptions to an allele that then was prone to expand in length from generation to generation. However, we have not been able to identify any alleles without repeat interruptions but with normal repeat length.

Recent additions to the list of genetically defined hereditary ataxias include two repeat-expansion disorders that were detected through NGS: intronic biallelic complex pentanucleotide *RFC1* expansions causing cerebellar ataxia, neuropathy, and vestibular areflexia syndrome (CANVAS [MIM: 614575])[1] and deep intronic monoallelic trinucleotide *FGF14* expansions causing late-onset spinocerebellar ataxia 27B (MIM: 620174).[2] Both disorders were soon shown to be relatively common causes for previously undiagnosed hereditary ataxias.[1,25] The fact that we identified five families with ZFHX3 repeat expansions from our center suggests a relatively high prevalence of this disorder, at least among persons with ataxia from southern Sweden. Future studies will, we hope, describe the prevalence of this disorder in other populations.[26]

Neuropathology revealed p62-positive intraneuronal inclusions, most of which were seen in the cells' nuclei. A

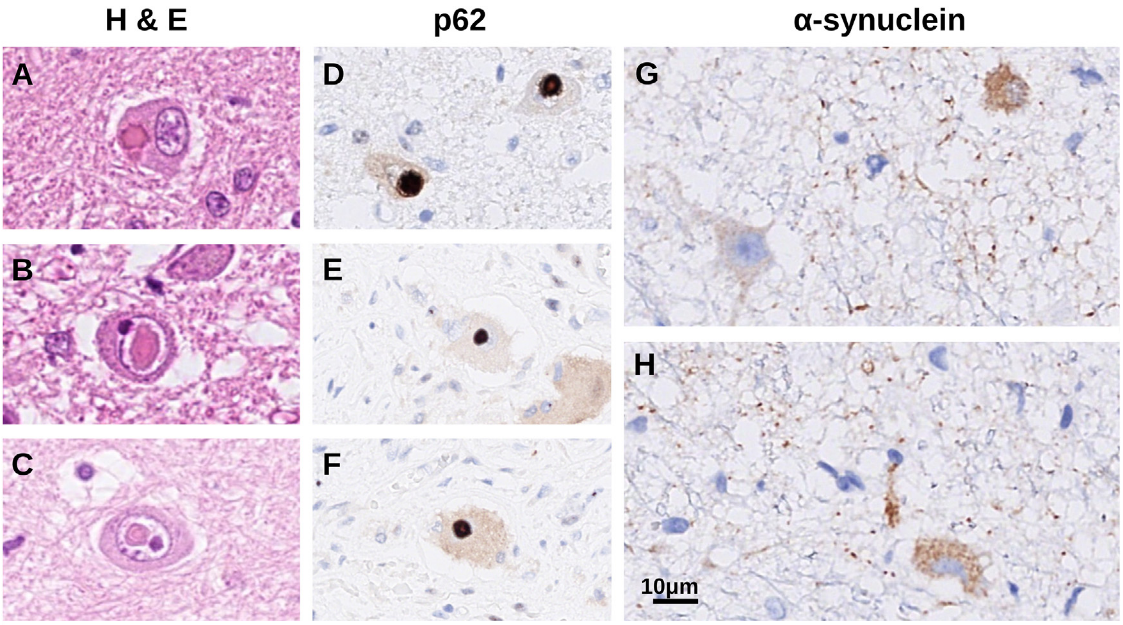

Figure 4. Neuropathology
(A–C) Hematoxylin and eosin (H & E)-stained tissue from the medulla oblongata with eosinophilic, intracytoplasmic (A), and intranuclear (B and C) inclusions in neurons.
(D) These inclusions stained positively with antibodies against p62.
(E and F) Similar immunoreactivity against p62 was seen in inclusions in neurons of the esophageal myenteric plexus.
(G and H) Alpha-synuclein-positive neurites and fine granular intracytoplasmic immunoreactivity in the medulla oblongata.

minority of nerve cells showed similar inclusions in the cytoplasm. Inclusions were seen in neurons of the central and peripheral nervous system, and in extraneuronal tissue. Pathological proteinaceous intranuclear or cytoplasmic depositions characterize many forms of spinocerebellar ataxia with poly-glutamine expansions; such forms include SCA1, 2, 3, 6, and 7.[27,28] Exonic repeat expansions that encode poly-glycine have so far not been associated with human disease. However, expansions of CGG trinucleotide repeats in the 5′-untranslated regions of several genes have been postulated to cause disease through specific mechanisms that result in the translation of poly-glycine.[29] These include fragile X tremor/ataxia syndrome (MIM: 300623),[30] an adult-onset disorder involving ataxia, and neuronal intranuclear inclusion disease (MIM: 603472).[31,32] In a large series, 39% of individuals with the latter disease had ataxia, and 92% had autonomic dysfunction.[33]

ZFHX3 is located between the markers D16S3019 and D16S512 on the long arm of chromosome 16, to which spinocerebellar ataxia type 4 (SCA4) had been mapped by conventional linkage analyses in a German and in two Swedish kindreds reported from Stockholm, Eastern Sweden.[34,35] The gene is in relative proximity to the locus reported in the original SCA4 kindred from Utah.[36] The relatively unique clinical phenotype in the families in the present study corresponds well with the phenotype described in SCA4, although we assume that examination and reporting methods have changed since the earlier descriptions of SCA4. Furthermore, the original Utah SCA4 kindred originated from Sweden.[36,37] We therefore suggest *ZFHX3* expansions to be the cause for SCA4.

There is one previous neuropathological report of a member of the large German SCA4 kindred. This man had disease signs since age 55, developed sensory loss, areflexia, and neurophysiological signs of axonal neuropathy; became wheelchair dependent; and died at age 70 years. No intracellular inclusions were reported, but staining for poly-glutamine aggregates was performed and was negative, and there was cell loss in the brain stem nuclei, cerebellum, and spinal cord.[38] The individual who was autopsied within our study had much younger onset and clinically more severe disease, and we hypothesize that poly-glycine inclusions might only be formed, or be formed more abundantly, in individuals who have early onset and who may have longer repeat expansions in *ZFHX3*.

Involving data from two large kindreds and three additional families, our study provides strong evidence on the genetic association of repeat expansions in *ZFHX3* with this specific clinical phenotype. Analyses of more than 2,300 non-expanded alleles revealed that normal alleles are considerably shorter than the read length of short-read NGS sequencing methods, and we have been able to correctly identify four additional individuals from three families through analyses of short-read data. Our study remains limited by the fact that we so far have been unable to determine the repeat expansions' exact lengths in all affected family members or to screen additional unaffected members to confirm that they

have normal repeat lengths, for example by an orthogonal method such as repeat-primed PCR. Such analyses from all family members with this disorder would also be necessary to validate the presumed inverse association of repeat length with age at symptom onset, as we have observed in long-read sequencing data from only two individuals.

We also have not been able to proceed to experiments that elucidate the functional effect of the expanded *ZFHX3* trinucleotide repeat on transcription or translation, or the effect of an expanded poly-glycine repeat on protein function. It is possible that the eosinophilic inclusions seen in the affected individual we report on here are composed of mutant ZFHX3 with longer-than-normal stretches of poly-glycine and without a serine interruption and that such mutant ZFHX3 proteins exert toxic effects. Other possible pathomechanisms include RNA toxicity, whereby transcripts with expanded uninterrupted repeats sequester RNA-binding proteins and form RNA foci.[29] Previous work has shown that ZFHX3 plays a role in neuronal differentiation[39–42] and that ZFHX3 is implied in cerebellar neurons' responses to oxidative stress,[43] plausibly linking variation in this gene to a cerebellar disorder. Furthermore, *ZFHX3* was identified as one of a few candidate genes for sporadic Hirschsprung disease,[44] which might connect *ZFHX3* to the pathology observed in the enteric nervous system and the clinical symptoms of diarrhea and fecal incontinence.

Unveiling the genetic cause of more and more cerebellar ataxias has made it possible to start developing targeted treatments.[45] We hope that our clinical and genetic observations might lay the ground for research on pathogenesis and pathomechanisms and ultimately help to develop treatment for individuals with this severely disabling disorder.

Data and code availability

Catalogs used for querying ExpansionHunter for the pathogenic *ZFHX3* repeat expansions, generated during this study, are provided in the supplement. There are restrictions to the availability of individual NGS datasets because of human subjects' confidentiality, national laws, regulations, and institutional practices; access to individual NGS datasets usually requires approval from the Swedish Ethical Review Authority.

Supplemental information

Supplemental information can be found online at https://doi.org/10.1016/j.ajhg.2023.11.008.

Acknowledgments

We thank the affected family members for their continued willingness to participate in our study over many years despite their worsening symptomatology, and we thank their relatives for supporting and participating in this study. We also thank all our neurologist colleagues who through years and decades examined and provided clinical care for the generations of affected individuals, and here we include those colleagues who contributed to the initial manuscript on the clinical observations of family 1 and 2;[4] their careful medical records were invaluable for us to summarize the clinical phenotype of this disorder. Long-read sequencing was performed at the SciLifeLab National Genomics Infrastructure in Uppsala. Computational analyses were performed on resources provided by SNIC through Uppsala Multidisciplinary Center for Advanced Computational Science (UPPMAX) under the SNIC project sens2016003. Open access publication fees were partly covered by Lund University. This study was funded by the Swedish government (through the ALF agreement), Region Skåne, Skåne University Hospital research funds and donations, MultiPark (a strategic research area at Lund University), The Swedish Parkinson Academy, Bundy Academy, Hans-Gabriel och Alice Trolle-Wachtmeisters stiftelse, Greta och Johan Kocks stiftelse, and SCA network, all in Sweden.

Author contributions

J.W.: conceptualization, methodology, software, validation, formal analysis, investigation (short read: repeat expansion and CNV analyses), data curation, writing—original draft (genetics sections), writing—review & editing.

E.K.: conceptualization, software, investigation (short read SNV data), formal analysis, data curation, writing—original draft (genetics sections), writing—review & editing.

E.J.: investigation (acquisition of clinical data), writing—original draft (case vignettes), writing—review & editing.

S.G.: investigation (acquisition of clinical data), writing—original draft (case vignettes), writing—review & editing.

A.Am.: methodology, formal analysis, investigation, resources, data curation (long-read sequencing), writing—original draft (long read sequencing sections), writing—review & editing.

C.K.: investigation (acquisition of clinical data), data curation.

S.D.: investigation (acquisition of clinical data), writing—review & editing.

K.K.: data curation, supervision, writing—review & editing.

T.K.: investigation (acquisition of clinical data: pediatric patients)

A.I.: resources (WGS datasets), writing—review & editing.

M.L.W.: resources (WGS datasets)

A.Ar.: investigation (acquisition of clinical data), writing—original draft (case vignettes)

S.P.: investigation (acquisition of clinical data)

E.E.: investigation (pathology), visualization, writing—review & editing.

H.E.: conceptualization, investigation (genetic data), resources, writing—original draft (genetics), writing—review & editing.

A.P.: conceptualization, methodology, investigation, resources, formal analysis, visualization, supervision, project administration, funding acquisition, writing—original draft (overall manuscript), writing—review & editing.

Declaration of interests

The authors declare no competing interests.

Received: October 23, 2023
Accepted: November 19, 2023
Published: November 29, 2023

References

1. Cortese, A., Simone, R., Sullivan, R., Vandrovcova, J., Tariq, H., Yau, W.Y., Humphrey, J., Jaunmuktane, Z., Sivakumar, P., Polke, J., et al. (2019). Biallelic expansion of an intronic repeat in RFC1 is a common cause of late-onset ataxia. Nat. Genet. *51*, 649–658.
2. Pellerin, D., Danzi, M.C., Wilke, C., Renaud, M., Fazal, S., Dicaire, M.J., Scriba, C.K., Ashton, C., Yanick, C., Beijer, D., et al. (2023). Deep Intronic FGF14 GAA Repeat Expansion in Late-Onset Cerebellar Ataxia. N. Engl. J. Med. *388*, 128–141.
3. Roberts, L.J., McVeigh, M., Seiderer, L., Harding, I.H., Corben, L.A., Delatycki, M., and Szmulewicz, D.J. (2022). Overview of the Clinical Approach to Individuals With Cerebellar Ataxia and Neuropathy. Neurol. Genet. *8*, e200021.
4. Wictorin, K., Brådvik, B., Nilsson, K., Soller, M., van Westen, D., Bynke, G., Bauer, P., Schöls, L., and Puschmann, A. (2014). Autosomal dominant cerebellar ataxia with slow ocular saccades, neuropathy and orthostatism: a novel entity? Parkinsonism Relat. Disorders *20*, 748–754.
5. Möller, E., Hindfelt, B., and Olsson, J.E. (1978). HLA–determination in families with hereditary ataxia. Tissue Antigens *12*, 357–366.
6. Trouillas, P., Takayanagi, T., Hallett, M., Currier, R.D., Subramony, S.H., Wessel, K., Bryer, A., Diener, H.C., Massaquoi, S., Gomez, C.M., et al. (1997). International Cooperative Ataxia Rating Scale for pharmacological assessment of the cerebellar syndrome. The Ataxia Neuropharmacology Committee of the World Federation of Neurology. J. Neurol. Sci. *145*, 205–211.
7. Schmitz-Hübsch, T., du Montcel, S.T., Baliko, L., Berciano, J., Boesch, S., Depondt, C., Giunti, P., Globas, C., Infante, J., Kang, J.S., et al. (2006). Scale for the assessment and rating of ataxia: development of a new clinical scale. Neurology *66*, 1717–1720.
8. Gorcenco, S., Kafantari, E., Wallenius, J., Karremo, C., Alinder, E., Dobloug, S., Landqvist Waldö, M., Englund, E., Ehrencrona, H., Wictorin, K., et al. (2023). Clinical and genetic analyses of a Swedish patient series diagnosed with ataxia. J. Neurol.
9. Browning, B.L., Tian, X., Zhou, Y., and Browning, S.R. (2021). Fast two-stage phasing of large-scale sequence data. Am. J. Hum. Genet. *108*, 1880–1890.
10. Zhou, Y., Browning, S.R., and Browning, B.L. (2020). A Fast and Simple Method for Detecting Identity-by-Descent Segments in Large-Scale Data. Am. J. Hum. Genet. *106*, 426–437.
11. McKenna, A., Hanna, M., Banks, E., Sivachenko, A., Cibulskis, K., Kernytsky, A., Garimella, K., Altshuler, D., Gabriel, S., Daly, M., and DePristo, M.A. (2010). The Genome Analysis Toolkit: a MapReduce framework for analyzing next-generation DNA sequencing data. Genome Res. *20*, 1297–1303.
12. McLaren, W., Gil, L., Hunt, S.E., Riat, H.S., Ritchie, G.R.S., Thormann, A., Flicek, P., and Cunningham, F. (2016). The Ensembl Variant Effect Predictor. Genome Biol. *17*, 122.
13. Robinson, J.T., Thorvaldsdóttir, H., Wenger, A.M., Zehir, A., and Mesirov, J.P. (2017). Variant Review with the Integrative Genomics Viewer. Cancer Res. *77*, e31–e34.
14. Firth, H.V., Richards, S.M., Bevan, A.P., Clayton, S., Corpas, M., Rajan, D., Van Vooren, S., Moreau, Y., Pettett, R.M., and Carter, N.P. (2009). DECIPHER: Database of Chromosomal Imbalance and Phenotype in Humans Using Ensembl Resources. Am. J. Hum. Genet. *84*, 524–533.
15. Dolzhenko, E., Deshpande, V., Schlesinger, F., Krusche, P., Petrovski, R., Chen, S., Emig-Agius, D., Gross, A., Narzisi, G., Bowman, B., et al. (2019). ExpansionHunter: a sequence-graph-based tool to analyze variation in short tandem repeat regions. Bioinformatics *35*, 4754–4756.
16. Rafehi, H., Read, J., Szmulewicz, D.J., Davies, K.C., Snell, P., Fearnley, L.G., Scott, L., Thomsen, M., Gillies, G., Pope, K., et al. (2023). An intronic GAA repeat expansion in FGF14 causes the autosomal-dominant adult-onset ataxia SCA50/ATX-FGF14. Am. J. Hum. Genet. *110*, 105–119.
17. Sone, J., Mitsuhashi, S., Fujita, A., Mizuguchi, T., Hamanaka, K., Mori, K., Koike, H., Hashiguchi, A., Takashima, H., Sugiyama, H., et al. (2019). Long-read sequencing identifies GGC repeat expansions in NOTCH2NLC associated with neuronal intranuclear inclusion disease. Nat. Genet. *51*, 1215–1221.
18. Dolzhenko, E., Bennett, M.F., Richmond, P.A., Trost, B., Chen, S., van Vugt, J.J.F.A., Nguyen, C., Narzisi, G., Gainullin, V.G., Gross, A.M., et al. (2020). ExpansionHunter Denovo: a computational method for locating known and novel repeat expansions in short-read sequencing data. Genome Biol. *21*, 102.
19. Dolzhenko, E., Weisburd, B., Ibañez, K., Rajan-Babu, I.S., Anyansi, C., Bennett, M.F., Billingsley, K., Carroll, A., Clamons, S., Danzi, M.C., et al. (2022). REViewer: haplotype-resolved visualization of read alignments in and around tandem repeats. Genome Med. *14*, 84.
20. Ameur, A., Dahlberg, J., Olason, P., Vezzi, F., Karlsson, R., Martin, M., Viklund, J., Kähäri, A.K., Lundin, P., Che, H., et al. (2017). SweGen: a whole-genome data resource of genetic variability in a cross-section of the Swedish population. Eur. J. Hum. Genet. *25*, 1253–1260.
21. Liao, W.W., Asri, M., Ebler, J., Doerr, D., Haukness, M., Hickey, G., Lu, S., Lucas, J.K., Monlong, J., Abel, H.J., et al. (2023). A draft human pangenome reference. Nature *617*, 312–324.
22. Björklund, E., Lindberg, E., Rundgren, M., Cronberg, T., Friberg, H., and Englund, E. (2014). Ischaemic brain damage after cardiac arrest and induced hypothermia–a systematic description of selective eosinophilic neuronal death. A neuropathologic study of 23 patients. Resuscitation *85*, 527–532.
23. Depienne, C., and Mandel, J.L. (2021). 30 years of repeat expansion disorders: What have we learned and what are the remaining challenges? Am. J. Hum. Genet. *108*, 764–785.
24. Chintalaphani, S.R., Pineda, S.S., Deveson, I.W., and Kumar, K.R. (2021). An update on the neurological short tandem repeat expansion disorders and the emergence of long-read sequencing diagnostics. Acta Neuropathol. Commun. *9*, 98.
25. Pellerin, D., Wilke, C., Traschütz, A., Nagy, S., Currò, R., Dicaire, M.J., Garcia-Moreno, H., Anheim, M., Wirth, T., Faber, J., et al. (2023). Intronic FGF14 GAA repeat expansions are a common cause of ataxia syndromes with neuropathy and bilateral vestibulopathy. J. Neurol. Neurosurg. Psychiatry, 2023–331490.
26. De Mattei, F., Ferrandes, F., Gallone, S., Canosa, A., Calvo, A., Chio, A., and Vasta, R. (2023). Epidemiology of Spinocerebellar Ataxias in Europe. Cerebellum.
27. Seidel, K., Siswanto, S., Brunt, E.R.P., den Dunnen, W., Korf, H.W., and Rüb, U. (2012). Brain pathology of spinocerebellar ataxias. Acta Neuropathol. *124*, 1–21.
28. Kumar, M., Tyagi, N., and Faruq, M. (2023). The molecular mechanisms of spinocerebellar ataxias for DNA repeat expansion in disease. Emerg. Top. Life Sci.
29. Liufu, T., Zheng, Y., Yu, J., Yuan, Y., Wang, Z., Deng, J., and Hong, D. (2022). The polyG diseases: a new disease entity. Acta Neuropathol. Commun. *10*, 79.
30. Sellier, C., Buijsen, R.A.M., He, F., Natla, S., Jung, L., Tropel, P., Gaucherot, A., Jacobs, H., Meziane, H., Vincent, A., et al.

(2017). Translation of Expanded CGG Repeats into FMRpolyG Is Pathogenic and May Contribute to Fragile X Tremor Ataxia Syndrome. Neuron *93*, 331–347.

31. Zhong, S., Lian, Y., Luo, W., Luo, R., Wu, X., Ji, J., Ji, Y., Ding, J., and Wang, X. (2021). Upstream open reading frame with NOTCH2NLC GGC expansion generates polyglycine aggregates and disrupts nucleocytoplasmic transport: implications for polyglycine diseases. Acta Neuropathol. *142*, 1003–1023.
32. Boivin, M., Deng, J., Pfister, V., Grandgirard, E., Oulad-Abdelghani, M., Morlet, B., Ruffenach, F., Negroni, L., Koebel, P., Jacob, H., et al. (2021). Translation of GGC repeat expansions into a toxic polyglycine protein in NIID defines a novel class of human genetic disorders: The polyG diseases. Neuron *109*, 1825–1835.e5.
33. Liu, M., Gao, Y., Yuan, Y., Liu, X., Wang, Y., Li, L., Zhang, X., Jiang, C., Wang, Q., Wang, Y., et al. (2023). A comprehensive study of clinicopathological and genetic features of neuronal intranuclear inclusion disease. Neurol. Sci. *44*, 3545–3556.
34. Hellenbroich, Y., Bubel, S., Pawlack, H., Opitz, S., Vieregge, P., Schwinger, E., and Zühlke, C. (2003). Refinement of the spinocerebellar ataxia type 4 locus in a large German family and exclusion of CAG repeat expansions in this region. J. Neurol. *250*, 668–671.
35. Engvall, M. (2020). Identification of Disease Genes in Rare Neurological Conditions (Karolinska Institutet: Doctoral thesis. Dept of Molecular Medicine and Surgery).
36. Flanigan, K., Gardner, K., Alderson, K., Galster, B., Otterud, B., Leppert, M.F., Kaplan, C., and Ptácek, L.J. (1996). Autosomal dominant spinocerebellar ataxia with sensory axonal neuropathy (SCA4): clinical description and genetic localization to chromosome 16q22.1. Am. J. Hum. Genet. *59*, 392–399.
37. Cloud, L.J., and Wilmot, G. (2012). Other spinocerebellar ataxias. Handb. Clin. Neurol. *103*, 581–586.
38. Hellenbroich, Y., Gierga, K., Reusche, E., Schwinger, E., Deller, T., de Vos, R.A.I., Zühlke, C., and Rüb, U. (2006). Spinocerebellar ataxia type 4 (SCA4): Initial pathoanatomical study reveals widespread cerebellar and brainstem degeneration. J. Neural. Transm. *113*, 829–843.
39. Ido, A., Miura, Y., and Tamaoki, T. (1994). Activation of ATBF1, a multiple-homeodomain zinc-finger gene, during neuronal differentiation of murine embryonal carcinoma cells. Dev. Biol. *163*, 184–187.
40. Ishii, Y., Kawaguchi, M., Takagawa, K., Oya, T., Nogami, S., Tamura, A., Miura, Y., Ido, A., Sakata, N., Hashimoto-Tamaoki, T., et al. (2003). ATBF1-A protein, but not ATBF1-B, is preferentially expressed in developing rat brain. J. Comp. Neurol. *465*, 57–71.
41. Jung, C.G., Kim, H.J., Kawaguchi, M., Khanna, K.K., Hida, H., Asai, K., Nishino, H., and Miura, Y. (2005). Homeotic factor ATBF1 induces the cell cycle arrest associated with neuronal differentiation. Development *132*, 5137–5145.
42. Fuller, T.D., Westfall, T.A., Das, T., Dawson, D.V., and Slusarski, D.C. (2018). High-throughput behavioral assay to investigate seizure sensitivity in zebrafish implicates ZFHX3 in epilepsy. J. Neurogenet. *32*, 92–105.
43. Kim, T.S., Kawaguchi, M., Suzuki, M., Jung, C.G., Asai, K., Shibamoto, Y., Lavin, M.F., Khanna, K.K., and Miura, Y. (2010). The ZFHX3 (ATBF1) transcription factor induces PDGFRB, which activates ATM in the cytoplasm to protect cerebellar neurons from oxidative stress. Dis. Model. Mech. *3*, 752–762.
44. Zhang, Z., Li, Q., Diao, M., Liu, N., Cheng, W., Xiao, P., Zou, J., Su, L., Yu, K., Wu, J., et al. (2017). Sporadic Hirschsprung Disease: Mutational Spectrum and Novel Candidate Genes Revealed by Next-generation Sequencing. Sci. Rep. *7*, 14796.
45. Coarelli, G., Coutelier, M., and Durr, A. (2023). Autosomal dominant cerebellar ataxias: new genes and progress towards treatments. Lancet Neurol. *22*, 735–749.

The American Journal of Human Genetics, Volume *111*

Supplemental information

Exonic trinucleotide repeat expansions in *ZFHX3* cause spinocerebellar ataxia type 4: A poly-glycine disease

Joel Wallenius, Efthymia Kafantari, Emma Jhaveri, Sorina Gorcenco, Adam Ameur, Christin Karremo, Sigurd Dobloug, Kristina Karrman, Tom de Koning, Andreea Ilinca, Maria Landqvist Waldö, Andreas Arvidsson, Staffan Persson, Elisabet Englund, Hans Ehrencrona, and Andreas Puschmann

Supplemental Note: Case Reports

Our previous publication[1] contained detailed information on these and additional members of both families as they were available at the time of that work, that were presented in the main text table and in the supplement esupp2.[1] The below pedigree illustrates which family members were described or examined in which of our two publications and indicates changes in numbering:

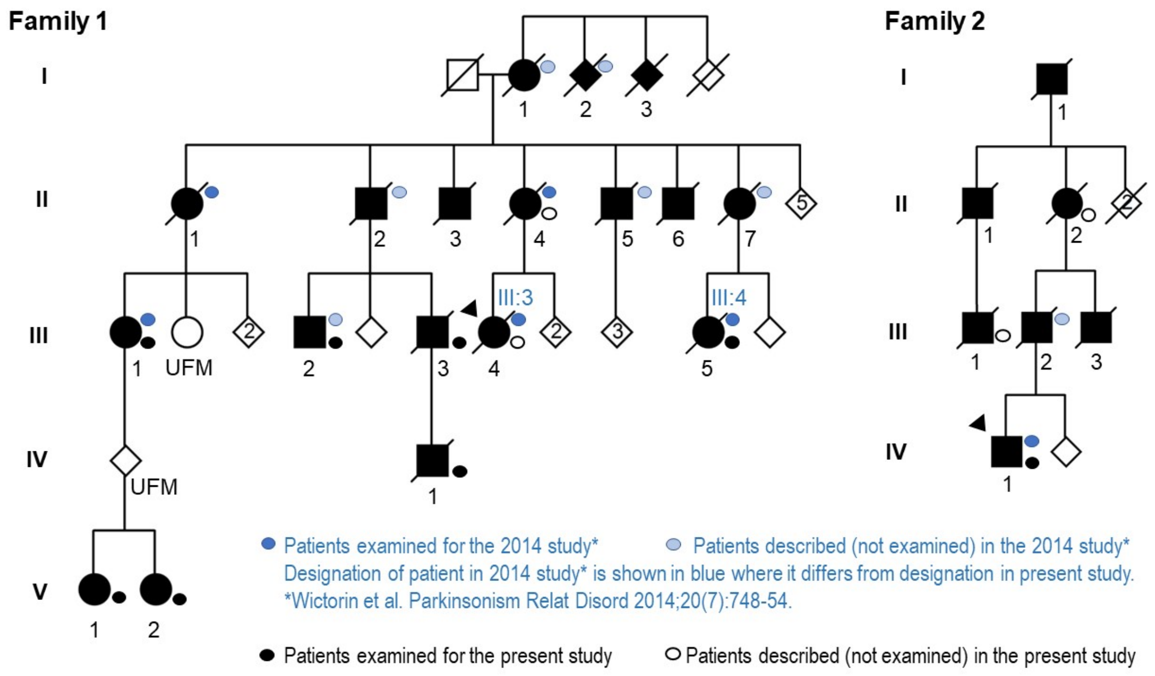

Case reports on the following pages summarize the clinical picture of affected individuals not previously published, those who were followed up in 2022-2023 and/or those for whom relevant new information had emerged since the original publication.

Family 1

II:4 Since the previous study, this individual had developed dysphagia and painful cramps in her left thigh. She also had several hospital admissions due to infections. She died 75 years old.

III:1 This family member was examined at the age of 66 years. Since the previous study this individual had started using a wheelchair and required turning devices to move between bed and wheelchair. BMI had fluctuated between 13.9 and 18.8 during the previous years. During the past year the individual had begun to choke while drinking and was referred to a speech therapist who confirmed dysphagia. She medicated for chronic constipation and hypertension and required a urinary catheter. The individual described episodes of involuntary jerky movements in the legs which had begun at age 63.

III:2 The existence of this affected family member was known to the research group in 2014, but he had previously not been available for physical examination. When examined for the first time in this study, he was 57 years old and had been using a wheelchair for five years. He had sensory impairment of all modalities in mainly the lower extremities with distal to proximal gradient, areflexia, intention tremor and fasciculations. Fasciculations were especially noticeable above his left knee. He experienced tingling sensations in his feet at night. The individual reported problems with bowel urgency but denied urinary symptoms and dizziness. The individual had medically treated hypertension.

III:3 This family member was not described in the previous study. He was had evaluated and followed at our neurology clinic but we had been unaware of his relationship to this kindred until after 2014. He was diagnosed with torticollis at the age of 25 which improved by age 45. At the age of 35, he was described as having an atactic gait and atactic limb movements along with diminished reflexes and positive Romberg's test. Reportedly he also had urinary retention. From the onset of ataxic symptoms, BMI varied between 12.2 to 17.4. At age 45, he refrained from using a walking aid despite incidents of falling each week. The following year he had hallucinations and several absence-like episodes with unclear etiology. He also started using a wheelchair and had developed severe ataxia, dysphagia, dysarthria and orthostatic hypotension. He had several hospital admissions due to repeated infections and died from sepsis and the age of 47 years.

III:4 ***(designated III:3 in previous publication[1])*** Since the previous study, this affected family member had received intensive therapy for her severe symptomatic hypotension/syncope, including midodrine, droxidopa and intermittent erythropoietin infusions. Despite these medications, she first used an office chair on rolls to move around in her home, as she would frequently faint when trying to stand up or walk. Later she resorted to a life near the floors, moving on all fours as her blood pressure also had become too low when sitting on a chair. She lost weight but declined a feeding tube. She died 42 years old.

III:5 ***(designated III:4 in previous publication[1])*** When this family member was examined at the age of 43, she could no longer stand without strong support of both arms and required support from another person when moving from wheelchair to bed. This was due to the progression of both ataxia and muscle wasting. A slightly inverted foot posture was noted at rest. She had developed mild dysphagia and a lack of appetite. BMI had varied between 11.7 and 14.4 during the previous year. She described a burning sensation in both hands and feet

both day and night, relieved slightly by gabapentin, an analgesic for neuropathic pain. There was sensory impairment of all tested modalities not only in her legs but also arms, with distal to proximal gradient. Family members described she had begun having episodes of sleep terror each night. She had also developed an inability to close her eyes properly for which she had received titanium weight implants in her eyelids. She lost more and more weight, declined a feeding tube, and died at age 44 years, weighing only 30 kg (BMI 11,7 kg/m^2), from marasmus and cardiac arrest.

IV:1 This individual was not described in the previous study because he had only just become 18 years old and since we were unaware of his connection to the family. He was diagnosed with atypical autism and reportedly had temper tantrums, panic attacks, and mild difficulties with verbal communication, concentration, and learning since childhood. Attention deficit hyperactivity disorder was mentioned in several medical records, but it was unclear whether this had been diagnosed on the basis of formal criteria. Balance disturbance was first noticed at the age of 15 years. By the age of 20, he had developed areflexia and problems with fine motor skills. Ataxia symptoms progressed and at the age of 23 he had mild dysarthria, wide-based and slowed gait, and reduced hand-foot coordination, but was still able to ride a bicycle. At 24, he had orthostatic hypotension and began sweating profusely during low-intensity physical activity, for example shorter walks and moving from bed to chair. The subsequent years he developed severe dysphagia and involuntary facial twitching, and had excessive airway mucus production and difficulty to expectorate. At 26, he required a walker at home and a wheelchair for longer distances. He had sensory impairment of all modalities in upper and lower extremities. He required clean intermittent urinary catheterization for several years and had recurring constipation. BMI during his last two years had decreased gradually from 20.4 to 15. He had percutaneous endoscopic gastrostomy performed at age 28 years but died the day after the gastrostomy from cardiac arrest and signs of severe infection. Postmortem examination revealed invasive mycosis with miliary spread to all examined inner organs, CNS, skin, and musculature. There was necrosis of gut, spleen, and liver.

V:1 (does not have the *ZFHX3* repeat expansion) This child was born after the data collection for the previous study. As an infant she was hypotonic and showed delayed psychomotor development, especially of her ability to walk. Her parents noticed uncoordinated movements when she was around 6 months old. According to her parents she was unable to stand on her own at 6 years of age. When examined at the age of 8 years, she was able to stand but still required the support of one hand. She used a walker, had a wide-based gait and everted feet. She preferred to move around by pushing herself forward on her knees but reportedly fell several times a day even from this position. She reportedly had a high pain threshold but occasionally experienced a painful sensation in her legs. According to her parents, she often choked while drinking. Her balance and coordination problems reportedly fluctuated over time. Myoclonic jerks were noticed in the hands during rest. Both somatic sensation and reflexes appeared normal. A general lack of subcutaneous fat, prominent superficial veins and joint hypermobility were observed. Several cognitive symptoms were described in her medical records and by her parents. The girl had an underdeveloped use of language, a mild intellectual disability, a strong need for daily routines, and was very selective about food, only eating certain food items. Her parents also reported severe behavioral problems with an inability to control emotion. She had intermittent constipation and had required a diaper since birth due to fecal and urinary incontinence. It was unclear whether the incontinence was of organic origin or due to cognitive impairment. She received antidepressants and sleep medication. When the child was examined, neither of the

parents showed any signs of the disease; the parent belonging to Family 1 (**IV:UFM** in Figure 1) was 37 years old at the time and was shown to have two non-expanded *ZFHX3* alleles.

V:2 (does not carry a *ZFHX3* repeat expansions) This child was born after 2014. No abnormality of normal muscle tone was noted in her infancy and early psychomotor development was normal, except for delayed gait. Her parents noticed unusual gait pattern and motor dyscoordination at 1.5 years of age. Six months prior to our examination the girl had begun to report intermittent tingling in her feet and occasional pain in her legs. The parents described that the girl's symptoms fluctuated over time; on bad days she was unable to climb stairs and was "shakier". When examined at the age of 4 years, she was able to walk without support, but preferred to run to keep a better balance. She was able to stand with both feet together and eyes open but could not stand in tandem. She was able to walk on her toes but not on her heels. Myoclonic jerks in the hands were noticed during rest. Somatic sensation appeared normal, but reflexes were diminished. The parents described that the daughter had once had an hour-long episode of cramping while remaining conscious, and an episode of fever preceded by turning blue around the mouth, both lacking obvious explanations. Toilet training was successful for a year, but at age 4 the girl had gone back to using a diaper because of urinary incontinence. She had constipation problems since birth. According to the parents, the girl had fallen behind in preschool and showed an apparent attention deficit. Neuropsychiatric investigation was pending.

Family 2

II:2 This individual was not described in the previous study because medical records had not been available. She developed a balance disturbance at the age of 43. The same year, neurologist's examination showed atactic gait, areflexia, and positive Babinski's sign bilaterally. Eye examination showed up-gaze palsy. Ten years later, she occasionally needed to support herself against a wall while walking, and fine motor skills had worsened, but she remained able to carry out regular housework chores. She had a sensory impairment of all modalities and mild dysmetria. She was troubled by pain in the lower back. She was described as dissimulating and anxious. She died 75 years old. According to notes in relatives' medical records, II:2's father I:1 had had balance disturbance for at least 10 years prior to his death at the age of 70.

III:1 This family member was briefly described in the previous study, but more detailed information has become available from medical records. At the age of 38, he noticed a difficulty to walk straight, especially in the dark, along with worsened fine motor skills. At 44 years, he had developed wide-based gait, dysarthria, dysmetria, and areflexia except for a weak triceps reflex response on both sides. Three years later, documentation described pathological saccadic ocular movement but without further specification. At 49, he had started using a walker. He fractured his femur at age 52, and thereafter used a wheelchair, but he was still able to move between bed and chair without support. He developed sensory impairment of all modalities in upper and lower extremities, complete areflexia, and positive Babinski's sign bilaterally. He was prescribed diazepam because of increased muscle tone in the legs at night. He also had medically treated hypertension. He had frequent bowel problems, both constipation and diarrhea. During his last years he obtained a long-term catheter due to urinary retention and nocturnal enuresis. He died 53 years old.

III:2 This individual has been described in our previous work. According to family members, this person had a brother (**III:3**) with similar neurological symptoms which began at the age of 20; **III:3** died at the age of 40 years. Medical records could not be retrieved due to missing personal data.

IV:1 Examination of this person at the age of 46 showed that ataxia and dysarthria had progressed since our previous study. He had been using a wheelchair for the past 7 years, and sensory impairment was present more proximally in the lower extremities than before. As in the previous study, he experienced no problems with dizziness but still struggled from bowel urgency and chronic diarrhea assessed as having a neurologic cause. Two years prior to this study, he had been hospitalized due to poor health status with BMI 16.6. At the time of our most recent examination, he had BMI 22.7 and muscle mass was well preserved in the upper body. He described a good appetite and no dysphagia. He was prescribed buspirone at his own request as he had heard the medication could mitigate ataxia. Despite lack of formal evidence for this indication, he felt the medication had a positive effect on overall wellbeing.
Clinical genetic testing revealed a novel truncating variant *SLC20A2* (NM_001257181.1) c.1240G>T, p.(Glu414*), interpreted as ”likely pathogenic” because of the known association of other truncating variants in this gene with primary basal ganglia calcification type 1 (MIM 213600).[2] On CT, there were mild calcifications in basal ganglia and cortical areas (below), in typical distribution for this genetic type of basal ganglia calcification *SLC20A2* gene, sparing the cerebellum. However, in a large case series, only 8% of patients with *SLC20A2*-related disease had ataxia, and penetrance regarding clinical symptoms is known to be incomplete

even in carriers of other *SLC20A2* variants who show intracerebral calcifications on CT scans.[3] No other examined member of Family 1 or 2 carried this variant. We thus considered the variant a cause of the calcifications but of minor or no relevance for this individual's clinical phenotype.

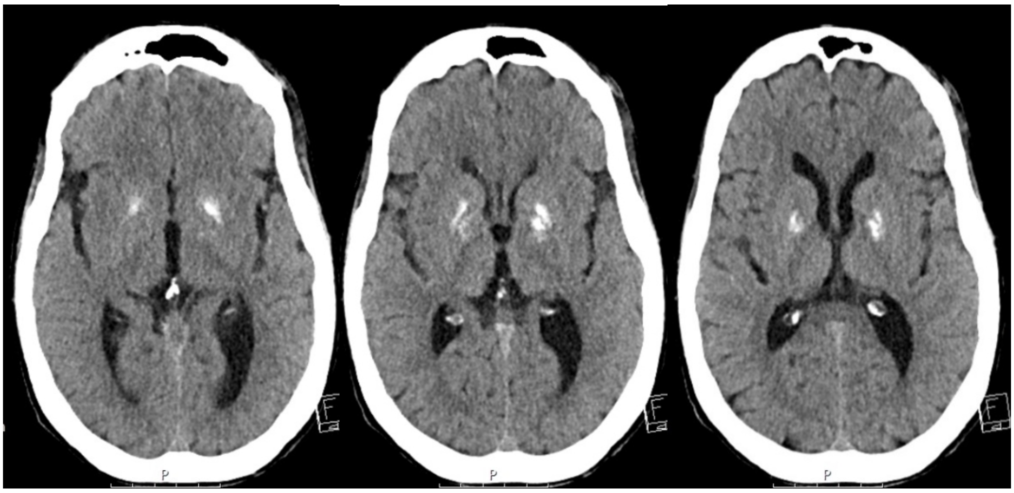

Family 3

According to information from the family, **I:1** had become unable to walk at higher age. **II:1** had balance problems starting in her 50s and died at age 68 years. **II:2** had very similar problems starting in his 50s. **III:2** had similar symptoms.

III:1 This male individual developed balance problems at age 57 years that had been progressive. He used a walker since age 63. On examination he had hearing loss, had slow and hypometric horizontal saccades, and rather turned his head than moved his eyes to look sidewards. He had mild cerebellar dysarthria. Sensibility for pain, vibration, proprioception, cold and light touch was decreased distally in legs and arms and no tendon reflexes could be elicited. Heel-knee-slide showed marked dysmetria and decomposition of movement, and there was mild to moderate ataxia in finger-nose test and dysdiadochokinesis in fast alternating hand movements. Nerve conduction studies at age 70 showed complete lack of sensory responses in median, ulnar and sural nerves bilaterally, and moderately decreased amplitudes in lower extremity motor nerves. An MRI of the brain at age 78 showed cerebellar atrophy:

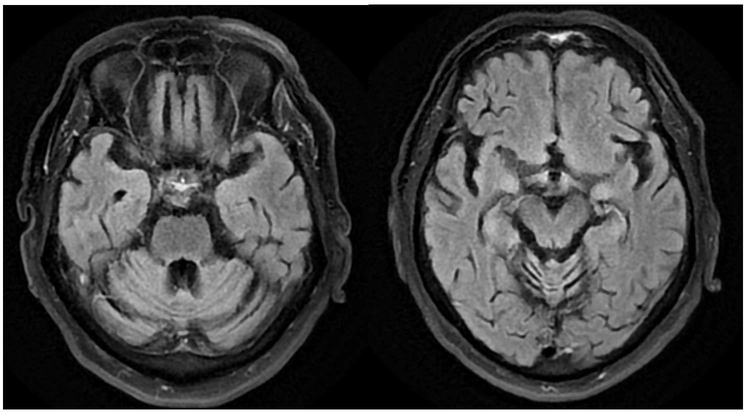

IV:1 **III:1**’s son had started to notice balance problems from age 44 years. When first seen at our department at age 47, there was ataxia of gait and in extremities and mild dystonic head movements. He was able to walk and perform physical work at 50 years of age, but by then had broad-based gait, nasal dysarthria. MRI showed mild cerebellar atrophy, especially of the anterior cerebellar lobe:

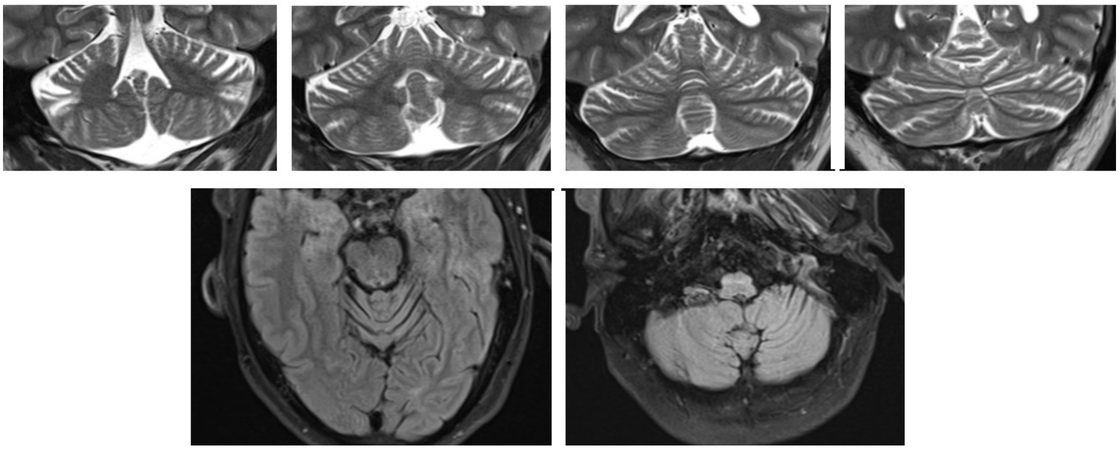

Family 4

IV:1 This female proband developed a balance disturbance at age 43. When examined within our research study, she had nasal speech, slightly impaired balance with signs of ataxia in upper and lower limbs and slight weakness distally in the lower limbs, difficulty walking on heels and elevating the front foot from the ground while standing. Reflexes were weak and vibration sense reduced in lower limbs. She reported nocturnal muscle cramps in the legs, in abdominal muscles, and bruxism. Tilt table test showed both immediate and delayed falls in blood pressure.

Seven relatives with similar symptoms were reported in the family (Figure 1).

The proband's grandmother (**II**, grey symbol in Figure 1) had gait problems, but details have been difficult to reconstruct; she died at age 64.

III:1 developed balance impairment at approximately 45 years of age. Subsequently, she had difficulties walking, at age 48 she developed severe leg pain and muscle cramps. During her last years of life, she had been unable to walk and needed much help with daily life activities such as eating, dressing and personal hygiene. She died at age 52.

Two maternal aunts of the proband, **III:2** and **III:3** had similar symptoms starting in their 40s and 50s, both eventually required a wheelchair. They died at 58 and 67 years of age.

Their three children, cousins to the proband, were all affected, with symptom onset at an earlier age:

IV:2 developed symptoms of impaired gait in adolescence and needed to use a walker early in the disease progression; she died at age 50.

IV:3 and **IV:4** developed symptoms of balance impairment at 29 and 30 years of age and died in their thirties after approximately 5 years disease duration. One of them had difficulties sitting or walking that were ascribed to severe back pain. According to information from the proband, the other male cousin preferably sat on the floor and moved around on the floor, without trying to stand up or use a wheelchair, as his disease had progressed. (This reminded us of what we saw in Family 1, **III:4**, where this was due to severe orthostatism.)

Family 5

According to family history, neither parent of **II:1** had gait or balance problems nor any other signs of neurological disease when they died at 79 and 86 years of age.

II:1 This male individual was followed at our department for over ten years; abundant medical records at our department were reviewed within this study, and additional information was received from his daughter. At about 50 years of age, he developed gait and balance disturbance that had progressed. A CT scan of the brain at 51 years of age showed moderate cerebellar atrophy, which was ascribed to a neurotoxic effect of solvents at the his workplace after evaluation at an occupational health department. He stopped working because of his disability at age 56. He needed to use a wheelchair since age 66. Neurologic examination at 67 years of age showed ataxic gait and limb ataxia. There was predominantly distal weakness in upper and lower extremities, muscle atrophy and some myokymia in these muscles. Sensation for the tested modalities pain, light touch and vibration was lost distally in upper and lower extremities. No tendon reflexes could be elicited. Nerve conduction studies at age 68 showed an axonal sensorimotor polyneuropathy, with predominant sensory findings and absence of all sensory responses. With increasing age, gait disturbance progressed, he lost ambulation, and developed dysarthria and dysphagia. Symptoms and signs of orthostatic hypotension were recorded but this was not assessed. Fasciculations in extremities and tongue were noted. A clinical diagnosis of hereditary sensorimotor neuropathy type 2 was entertained but no genetic testing had been performed. He died in the hospital at 79 years of age from respiratory failure after aspiration pneumonia that was aggravated by chronic obstructive pulmonary disease. This individual had no siblings.

III:1 This female family member had progressive gait disturbance with balance problems since about 40 years of age, which has remained the dominating symptom. She also experienced dysesthesias in lower extremities, mild subjective dysphagia and restless legs symptoms. Urinary urgency, constipation and dizziness when standing up also occurred. Nerve conduction studies showed absence of sensory responses in all nerves examined. Orthostatic test verified orthostatic hypotension on several occasions. At the time of referral, she used walking sticks. Neurological examination at our center at 50 years of age showed ataxic gait and immediate loss of balance on Romberg's test. She had ataxia during heel-knee-shin-slide that improved when she was asked to look at her movements. These findings were interpreted as sensory ataxia. However, her MRI at age 51 years showed moderate cerebellar atrophy. She had severe loss of vibration sensation in upper and lower extremities, milder distal loss of touch and pain sensation in the lower extremities and areflexia. No dysarthria, eye movement disturbance, muscle atrophy, fasciculations, weakness or upper motor neuron signs were found.

Supplemental Figures and Legends

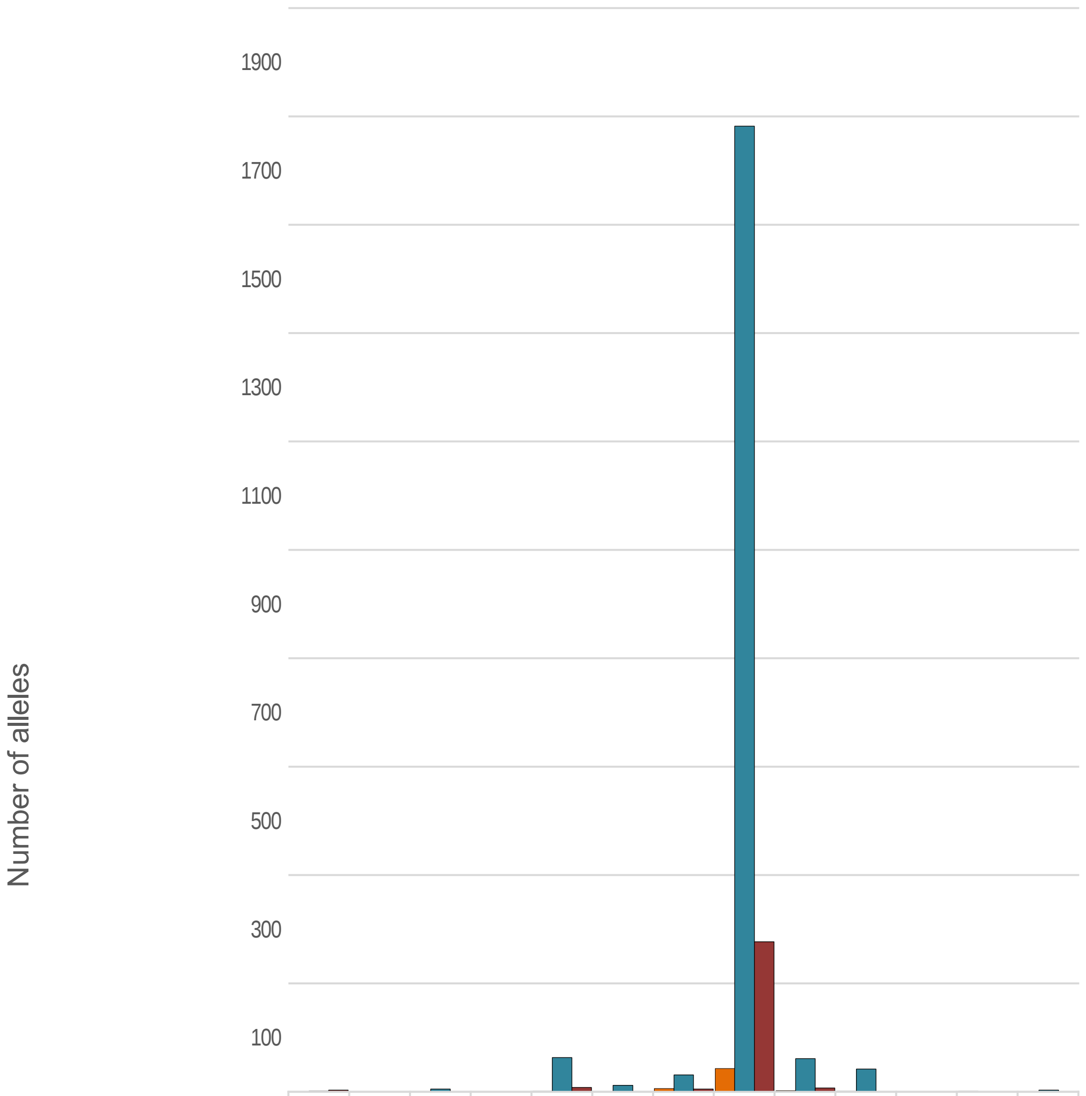

	14	15	16	17	18	19	20	21	22	23	24	25	26
27 Long Read controls	0	0	0	0	1	0	6	43	2	1	0	1	0
SweGene	1	0	5	0	63	12	31	1782	61	42	0	0	3
In-house dataset	3	0	0	0	8	0	5	277	7	0	0	0	0

Figure S1: Distribution of non-expanded alleles (details)
The distribution of total repeat length, including interruptions, in 2,000 non-expanded alleles from the SweGen database (blue), 300 alleles of our in-house NGS datasets (red), and 52 long-read alleles from the Human Pangenome Reference Consortium (orange). Below the diagram are the exact counts of allele lengths. It is based on short-read WGS and WES data, as well as long-read WGS data.

Figures S2-S15: Sequencing images

The images in this section were all produced with REViewer (short-read data, Figures S2-S13) or TRVZ (long-read data, Figures S14 and S15; https://doi.org/10.1101/2023.05.12.540470). For each individual, the normal allele is shown first, above the expanded allele. Sequence is depicted along the positive reference strand, so that, for example, GGC is shown as CCG, and *ZFHX3* transcription runs from right to left. In the REViewer images, flanking and AGT interruption sequence is colored blue, GGY sequence downstream of the AGT interruption is colored orange, GGY sequence upstream of the AGT interruption is colored green. In the TRVZ images, flanking sequence is colored green, while GGC sequence is colored blue, and any mismatches owing to interruptions are colored grey.

Short-read data with presupposed AGT interruption (Figures S2-S9)

Genotyping with a presupposed AGT interruption was successful for normal alleles, where the AGT unit is present. However, the expanded alleles showed no sign of interruptions – the reads were thus forced to map to either side of the reference AGT interruption, causing a failure of the total length estimation.

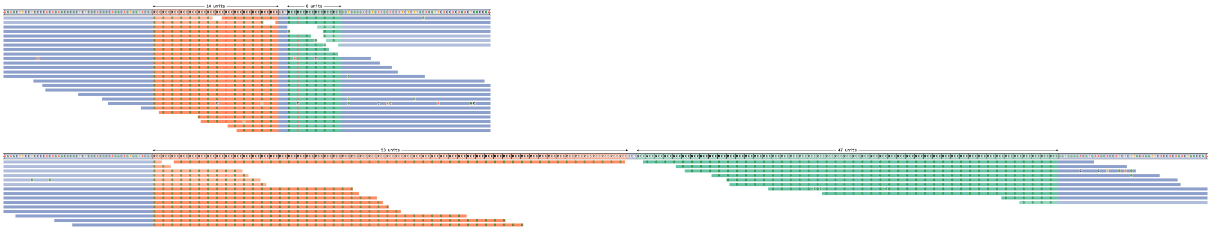

Figure S2: Family 1 III:1.

A canonical non-expanded allele, and an expanded allele. The true length of the expanded allele was determined with long-read sequencing to 57 GGC units, for comparison.

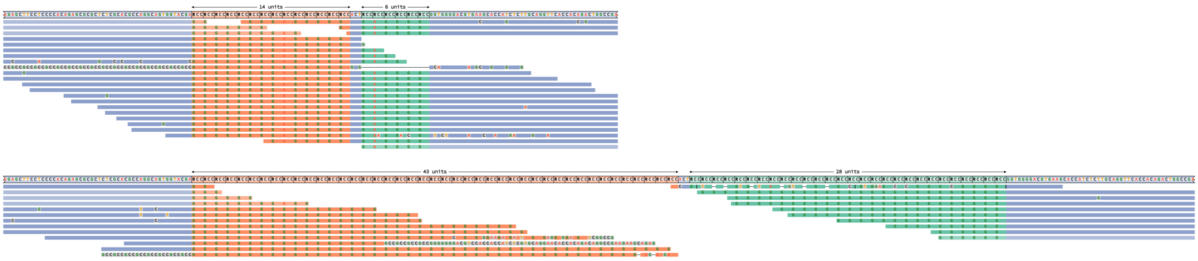

Figure S3: Family 1 III:2.

A canonical non-expanded allele, and an expanded allele.

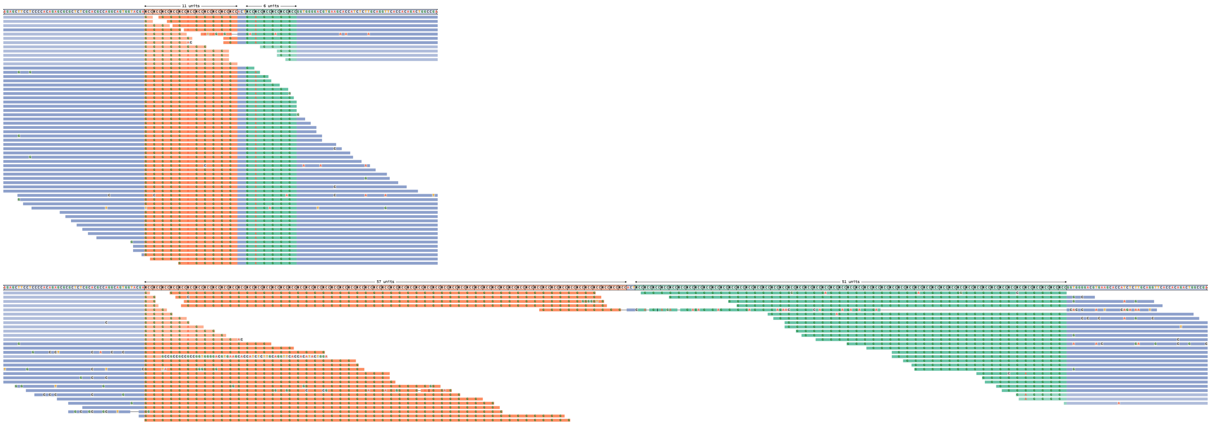

Figure S4: Family 1 IV:1

A non-expanded allele three units shorter than usual, and an expanded allele. Read depth is remarkably better than in other individuals. The length of this expanded allele was determined to 74 with long-read sequencing, for comparison.

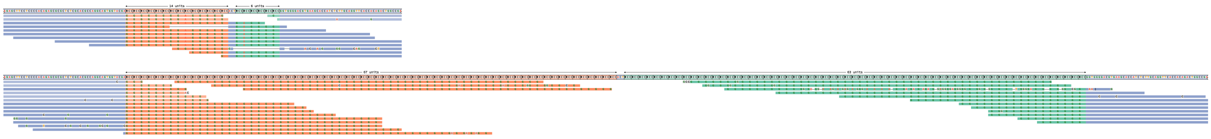

Figure S5: Family 2 IV:1

A canonical non-expanded allele, and an expanded allele.

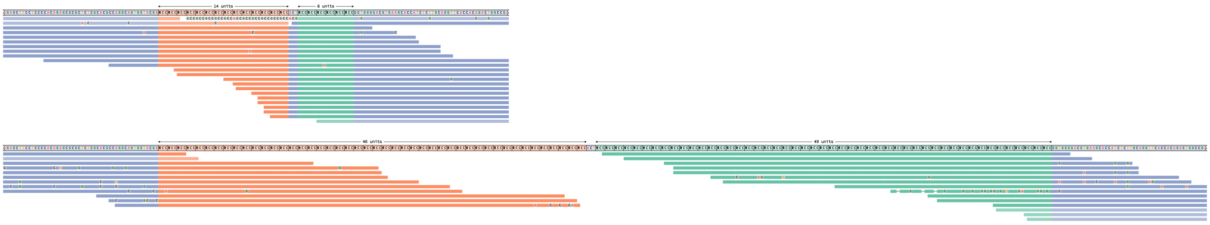

Figure S6: Family 3 III:1

A canonical non-expanded allele, and an expanded allele.

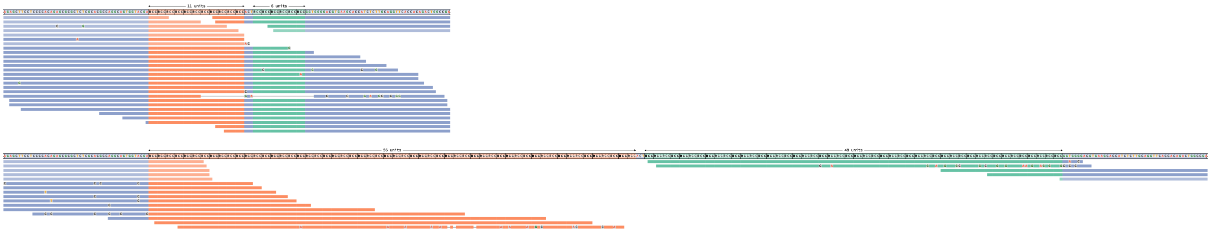

Figure S7: Family 3 IV:1

A non-expanded allele three units shorter than usual, and an expanded allele.

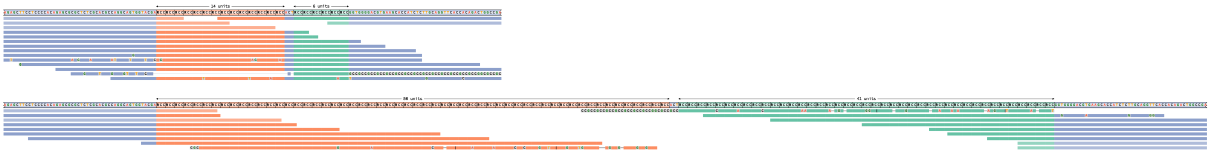

Figure S8: Family 4 IV:1

A canonical non-expanded allele, and an expanded allele

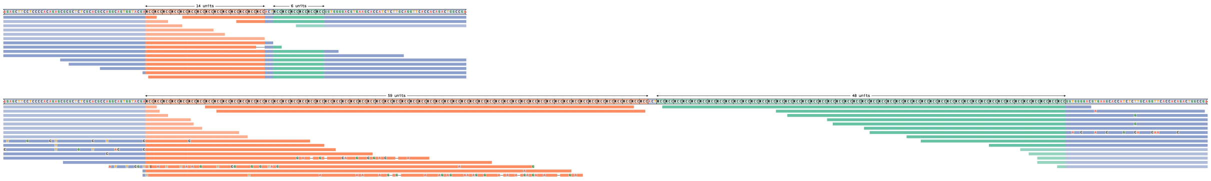

Figure S9: Family 5 III

A canonical non-expanded allele, and an expanded allele.

Short-read data without presupposed AGT interruption (Figures S10-S13)

Genotyping without presupposing the common AGT interruption results in better length estimates for alleles that do not have the AGT interruption. For expanded alleles, the genotyped length is an estimate, or minimum length. For non-expanded alleles, the genotyped length is exact regardless of whether there are AGT units present or not. If present, the AGT interruption shows up as sequence mismatch.

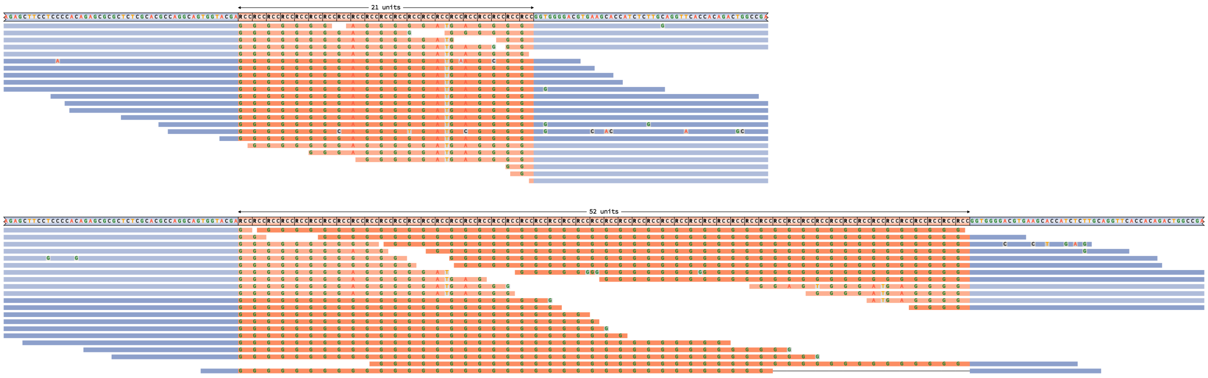

Figure S10: Family 1 III:1

A canonical non-expanded allele, and an expanded allele. The length estimate of the expanded allele of 52 units was close to the 54 units detected by long-read sequencing of this individual.

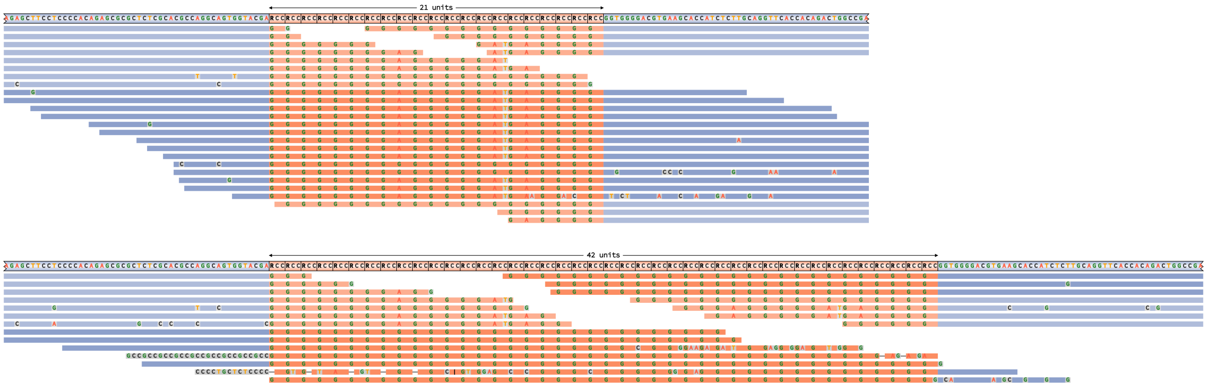

Figure S11: Family 1 III:2

A canonical non-expanded allele, and an expanded allele.

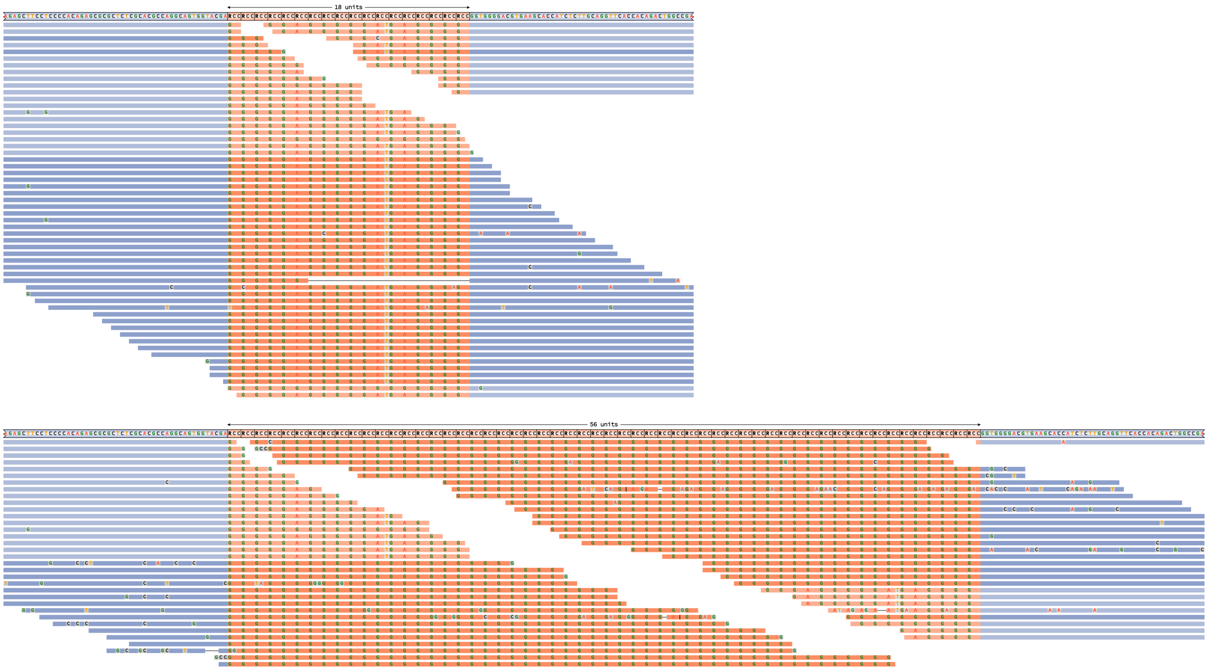

Figure S12: Family 1 IV:1

A non-expanded allele three units shorter than usual, and an expanded allele. The length estimate using short-read sequencing was 56 units, underestimating the length of 74 units as disclosed by long-read sequencing of this individual.

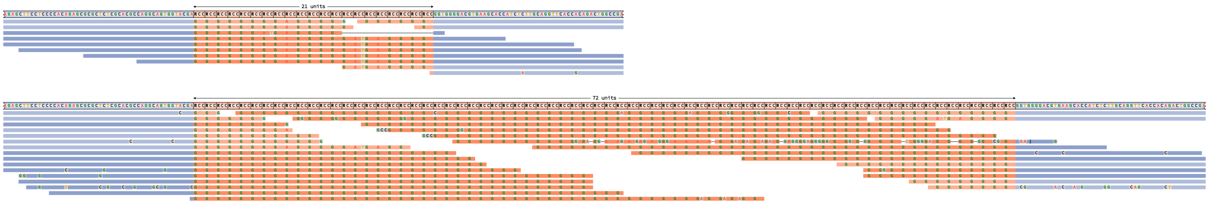

Figure S13: Family 2 IV:1

A canonical non-expanded allele, and an expanded allele.

Long-read data without presupposed AGT interruption (Figures S14-S15)

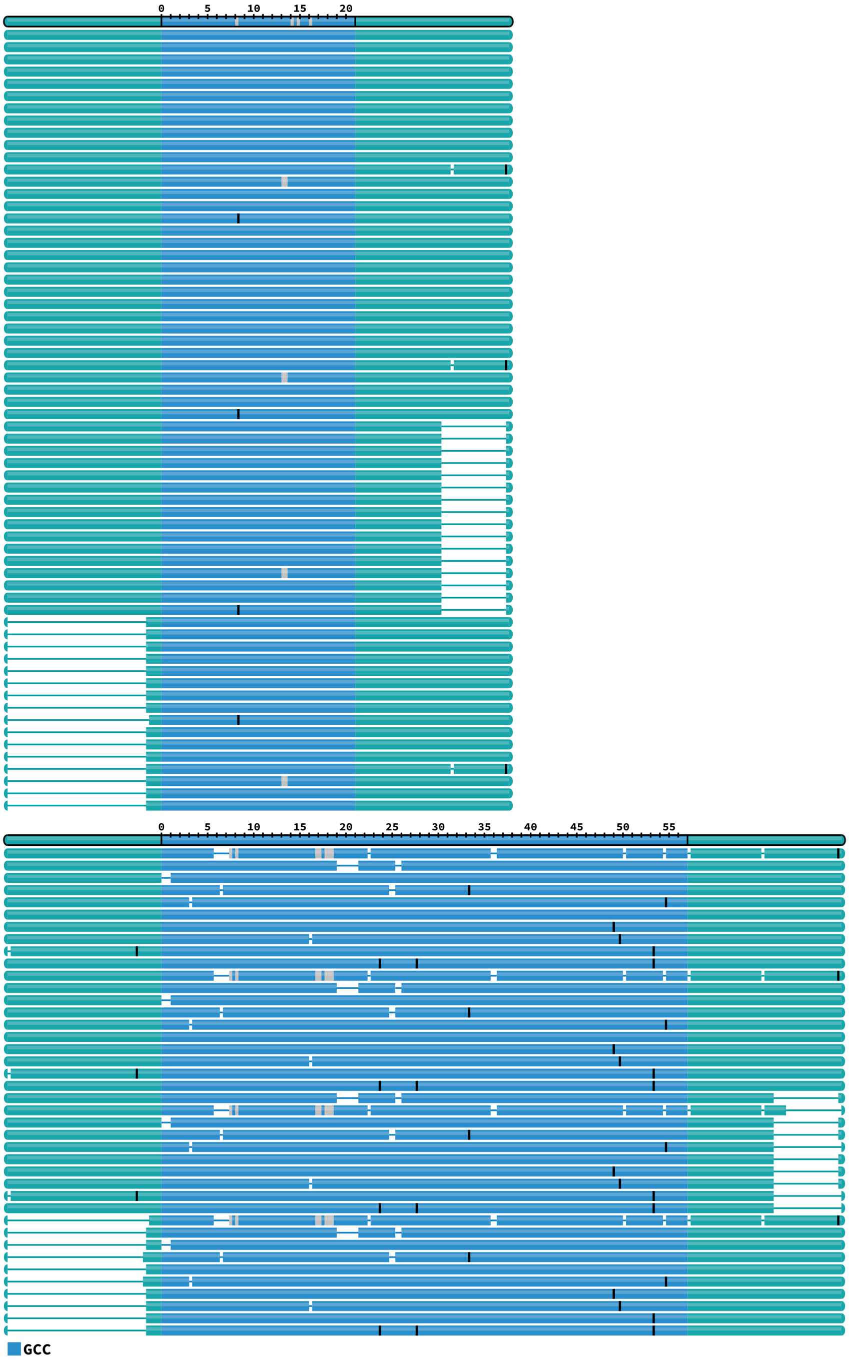

Figure S14: Family 1 III:1

This individual carried one canonical non-expanded allele (top) and one expanded allele (bottom). Black bars indicate insertions, horizontal lines indicate deletions. There are reads that perfectly span the repeat region. Estimated length is 57 trinucleotide units.

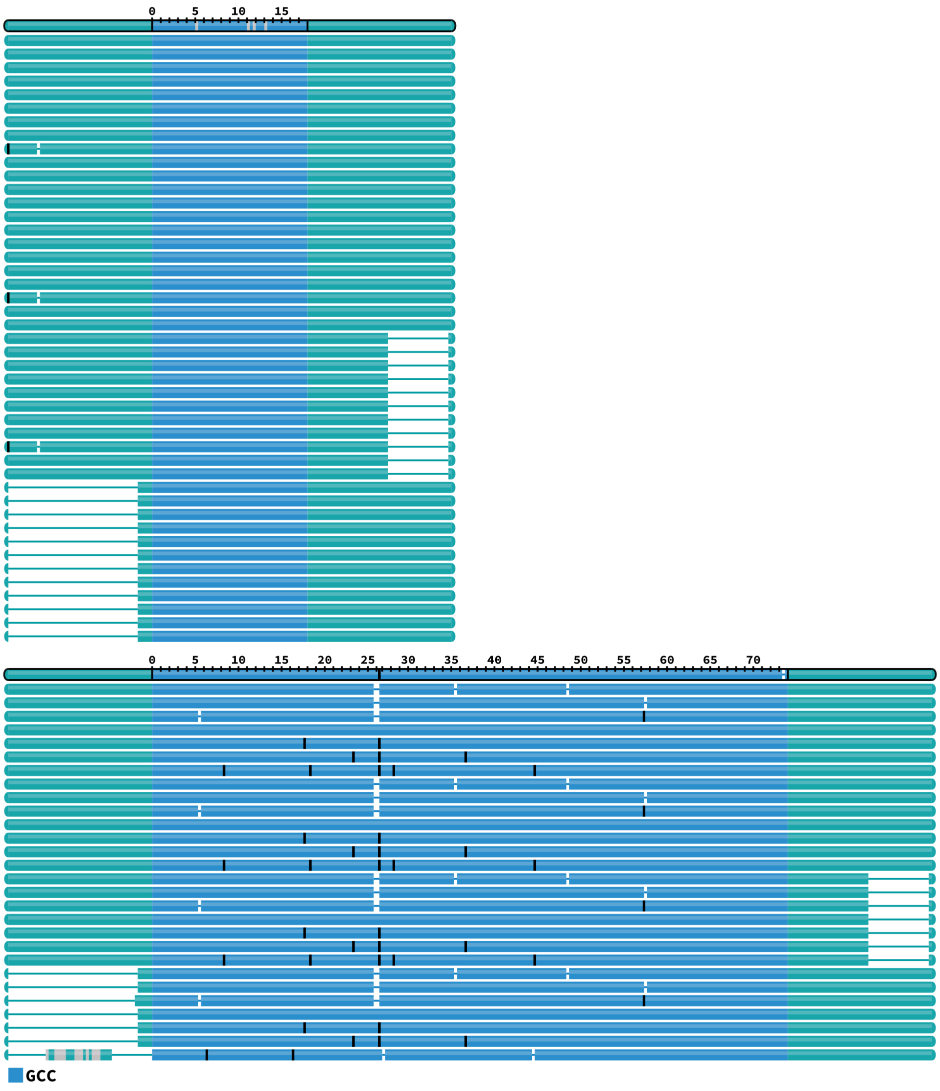

Figure S15: Family 1 IV:1

A non-expanded allele three units shorter than usual, and an expanded allele. Black bars indicate insertions, horizontal lines indicate deletions. There are reads that perfectly span the repeat region. Estimated length is 74 units.

Supplemental Tables

families	chr	start	end	length [kbp]	min-seed	min-extend	min-output	min-mac	min-markers
1	16	72,676,268	74,358,156	1,682	1	0.5	1	6	80
1, 2	16	72,676,268	73,070,083	394	0.5	0.3	0.5	6	60
1, 2, 3	16	72,676,268	73,070,083	394	0.3	0.15	0.3	6	45
1, 2, 3, 4	16	72,676,268	72,987,637	311	0.3	0.15	0.3	6	45
1, 2, 3, 4, 5	16	72,725,757	72,836,593	111	0.05	0.02	0.05	6	20

Table S1: Genomic segments shared by affected individuals in Families 1-5
Coordinates and lengths of the genomic segments shared by affected individuals from Families 1–5 as indicated in the first column, as identified by hap-ibd. The segment shared by all five families was 111 kpb in length (bottom row). Families 2, 4, and 5 are in this analysis represented by their respective probands. Family 1 is represented by individuals III:1, III:2, and IV:1, family 3 is represented by only individual III:1. The last five columns are input parameters to the hap-ibd program. Analyses from Family 1 and 2 were used prior to the identification of the *ZFHX3* repeat expansions, analyses from Families 3–5 were added later to better define an underlying shared haplotype. Reference genome hg19.

chr	start	end	motif	gene	region	p-value	Bonf. p-value
16	72,821,250	72,822,088	CCG	ZFHX3	exonic	0.07864	1.0

Table S2: *ZFHX3* as found by ExpansionHunter Denovo
P-values both before and after Bonferroni correction are given. The coordinates are to be considered approximate.

	Marker	Coordinates on chr 16
**Utah SCA4 family of Swedish origin**[4]		
Tight linkage reported	D16S397	66,738,337
Haplotype block with D16S397 in affected individuals	D16S398 – D16S421	66,129,139 – 67,295,807
**German SCA4 family**[5]		
Linked interval	D16S3019 – D16S512	66,129,331 – 74,067,749
**Two Swedish SCA4 families reported from Stockholm**[6]		
Linked interval	Same as above in German SCA4 family[5]	
**This report**		
Shared genomic segment* within in Family 1		72,676,268 – 74,358,156
Shared genomic segment* between families 1 and 2		72,676,268 – 73,070,083
Shared genomic segment* between families 1, 2, and 3		72,676,268 – 73,070,083
Shared genomic segment* between families 1, 2, 3, and 4		72,676,268 – 72,987,637
Shared genomic segment* between families 1, 2, 3, 4, and 5		72,725,757 – 72,836,593
***ZFHX3* GGY repeat**		
ZFHX3 NM_006885.4 exon 10 GGY repeat		72,821,593 – 72,821,656

Table S3: Comparison with previously reported SCA4 loci
The shared genomic segments were identified by hap-ibd. See Table S2 legend and main text for details. Reference numbers refer to the reference list in the main article.

hg19	hg38	ref	alt	rsID	GT	1KG p3	gnomADg	SweFreq
72,729,354	72,695,455	C	T	rs1041588471	0/1	n/a	0.00001546	n/a
72,768,356	72,734,457	A	G	rs183642752	0/1	0.001	0.001146	n/a
72,771,602	72,737,703	T	C	n/a	0/1	n/a	n/a	n/a

Table S4: Very rare SNVs within the IBD-ZFHX3 locus
These SNVs with very low frequencies were found in the genomic segment shared by all individuals with the repeat expansion, but not found in individuals without repeat expansion. The 1000 Genomes (1KG) frequencies are from the European population. The gnomAD genome frequencies are from the non-Finnish European population. SweFreq is based on the SweGen control data used in the manuscript.

Type of variation	Count	Frequency	Length	no. GGT	no. GAC	no. AGT	no. CGC
Normal	1935	84.13%	21	2	0	1	0
GGC > GAC	78	3.39%	21	2	1	1	0
+1 GGC 3'	64	2.78%	22	2	0	1	0
-3 GGC 3'	59	2.57%	18	2	0	1	0
+2 GGC 3'	36	1.57%	23	2	0	1	0
GGT > GGC 3'	24	1.04%	21	1	0	1	0
GGC > CGC 5'	22	0.96%	21	2	0	1	1
-1 GGT 5'	21	0.91%	20	1	0	1	0
-1 GGT, -1 GGC 3'	11	0.48%	19	1	0	1	0
-1 GGC 3'i	10	0.43%	20	2	0	1	0
-1 GGT, -2 GGC 3'	6	0.26%	18	1	0	1	0
-2 GGC 3'i, -1 GGT 5'	5	0.22%	18	1	0	1	0
7GGC,GGT,8GGC	5	0.22%	16	1	0	0	0
+1 GGT, +1 AGT 5'i	3	0.13%	23	2	0	2	0
+5 GGT 3'	3	0.13%	26	2	0	1	0
AGT > GGT	3	0.13%	21	3	0	0	0
-1 GGT, -6 GGC 3'	2	0.09%	14	1	0	1	0
-1 GGC 3'	2	0.09%	20	2	0	1	0
-5 GGC 5', -1 GGT 5', -1 AGT	2	0.09%	14	1	0	0	0
GGC > GGT 5'	1	0.04%	21	3	0	1	0
+2 GGC 3', GGC > CGC 5'	1	0.04%	23	2	0	1	1
+2 GGC 5'	1	0.04%	23	2	0	1	0
-1 GGC 5'	1	0.04%	20	2	0	1	0
GGC > GAC, -1 GGC 3'	1	0.04%	20	2	1	1	0
-2 GGC 3'	1	0.04%	19	2	0	1	0
6GGC,GGT,16GGC	1	0.04%	23	1	0	0	0
6GGC,GGT,15GGC	1	0.04%	22	1	0	0	0

Table S5: Variations in non-expanded alleles

The structure of 2,300 non-expanded alleles was analysed in detail. There were four types of deviations from the GGC repeat unit. These are, in order of prevalence, GGT, AGT, GAC, CGC. The CGC unit was found to always be located at the 5’ end of the repeat region, and can thus not be considered a true interruption. The GAC unit was found to always be located three units downstream of the AGT unit. The GGT units were observed in various locations, although never adjacent to each other, nor terminal (at any ends of the GGC repeats). In the vast majority of alleles, with two GGT units, the first unit is located two units upstream of the AGT unit, while the second unit is located six units downstream of the AGT unit (cf. Figure 2 for positions). The descriptions in the first column are either relative (to the most frequent normal allele as shown in Figure 2A), or absolute (row 13 and the two last rows). The latter type of description is used when the structure of the repeat deviates significantly from the normal. The 3’ and 5’ tags mean that the change from normal structure occurs at or near the downstream and upstream ends, respectively. An added “i” means the change occurred between the normal allele’s AGT and GGT units.

Expansion Hunter catalogs

```
[
    {
        "LocusId": "ZFHX3",
        "LocusStructure": "(RCC)*",
        "ReferenceRegion": "16:72821593-72821656",
        "VariantId": "ZFHX3",
        "VariantType": "Repeat"
    }
]
```

The *ZFHX3* STR in json format for use with ExpansionHunter, coordinates in hg19.

```
[
    {
        "LocusId": "ZFHX3",
        "LocusStructure": "(RCC)*",
        "ReferenceRegion": "16:72787694-72787757",
        "VariantId": "ZFHX3",
        "VariantType": "Repeat"
    }
]
```

The *ZFHX3* STR in json format for use with ExpansionHunter, coordinates in hg38.

Supplemental References

1. Wictorin, K., Brådvik, B., Nilsson, K., Soller, M., van Westen, D., Bynke, G., Bauer, P., Schols, L., and Puschmann, A. (2014). Autosomal dominant cerebellar ataxia with slow ocular saccades, neuropathy and orthostatism: a novel entity? Parkinsonism Relat Disord *20*, 748-754. 10.1016/j.parkreldis.2014.03.029.

2. Ramos, E.M., Carecchio, M., Lemos, R., Ferreira, J., Legati, A., Sears, R.L., Hsu, S.C., Panteghini, C., Magistrelli, L., Salsano, E., et al. (2018). Primary brain calcification: an international study reporting novel variants and associated phenotypes. Eur J Hum Genet *26*, 1462-1477. 10.1038/s41431-018-0185-4.

3. Batla, A., Tai, X.Y., Schottlaender, L., Erro, R., Balint, B., and Bhatia, K.P. (2017). Deconstructing Fahr's disease/syndrome of brain calcification in the era of new genes. Parkinsonism Relat Disord *37*, 1-10. 10.1016/j.parkreldis.2016.12.024.

4. Flanigan, K., Gardner, K., Alderson, K., Galster, B., Otterud, B., Leppert, M.F., Kaplan, C., and Ptacek, L.J. (1996). Autosomal dominant spinocerebellar ataxia with sensory axonal neuropathy (SCA4): clinical description and genetic localization to chromosome 16q22.1. Am J Hum Genet *59*, 392-399.

5. Hellenbroich, Y., Bubel, S., Pawlack, H., Opitz, S., Vieregge, P., Schwinger, E., and Zuhlke, C. (2003). Refinement of the spinocerebellar ataxia type 4 locus in a large German family and exclusion of CAG repeat expansions in this region. J Neurol *250*, 668-671. 10.1007/s00415-003-1052-x.

6. Engvall, M. (2020). Identification of disease genes in rare neurological conditions (Doctoral thesis. Dept. of Molecular Medicine and Surgery, Karolinska Institutet).
